# High-fidelity wheat plant reconstruction using 3D Gaussian splatting and neural radiance fields

**DOI:** 10.1093/gigascience/giaf022

**Published:** 2025-03-26

**Authors:** Lewis A G Stuart, Darren M Wells, Jonathan A Atkinson, Simon Castle-Green, Jack Walker, Michael P Pound

**Affiliations:** School of Computer Science, University of Nottingham, Nottingham, NG8 1BB, UK; School of Biosciences, University of Nottingham, Nottingham, LE12 5RD, UK; School of Biosciences, University of Nottingham, Nottingham, LE12 5RD, UK; School of Computer Science, University of Nottingham, Nottingham, NG8 1BB, UK; School of Biosciences, University of Nottingham, Nottingham, LE12 5RD, UK; School of Computer Science, University of Nottingham, Nottingham, NG8 1BB, UK

**Keywords:** 3D Gaussian splatting, 3DGS, neural radiance fields, NeRF, view synthesis, machine learning, 3D reconstruction, digital twin, robotics, phenotyping, imaging

## Abstract

**Background:**

The reconstruction of 3-dimensional (3D) plant models can offer advantages over traditional 2-dimensional approaches by more accurately capturing the complex structure and characteristics of different crops. Conventional 3D reconstruction techniques often produce sparse or noisy representations of plants using software or are expensive to capture in hardware. Recently, view synthesis models have been developed that can generate detailed 3D scenes, and even 3D models, from only RGB images and camera poses. These models offer unparalleled accuracy but are currently data hungry, requiring large numbers of views with very accurate camera calibration.

**Results:**

In this study, we present a view synthesis dataset comprising 20 individual wheat plants captured across 6 different time frames over a 15-week growth period. We develop a camera capture system using 2 robotic arms combined with a turntable, controlled by a re-deployable and flexible image capture framework. We trained each plant instance using two recent view synthesis models: 3D Gaussian splatting (3DGS) and neural radiance fields (NeRF). Our results show that both 3DGS and NeRF produce high-fidelity reconstructed images of a plant subject from views not captured in the initial training sets. We also show that these approaches can be used to generate accurate 3D representations of these plants as point clouds, with 0.74-mm and 1.43-mm average accuracy compared with a handheld scanner for 3DGS and NeRF, respectively.

**Conclusion:**

We believe that these new methods will be transformative in the field of 3D plant phenotyping, plant reconstruction, and active vision. To further this cause, we release all robot configuration and control software, alongside our extensive multiview dataset. We also release all scripts necessary to train both 3DGS and NeRF, all trained models data, and final 3D point cloud representations. Our dataset can be accessed via https://plantimages.nottingham.ac.uk/ or https://https://doi.org/10.5524/102661. Our software can be accessed via https://github.com/Lewis-Stuart-11/3D-Plant-View-Synthesis.

## Introduction

In recent years, 3-dimensional (3D) reconstruction of plants has become an important tool in plant phenotyping pipelines. Generating a 3D representation of a plant facilitates effective extraction of key traits and simplifies the analysis of complex plant structure. The ability to accurately capture these traits in 3D provides valuable information for determining a plant’s growth rate, health, and stress factors [1]. Plant leaves (and the canopies they form) are inherently 3D structures, and factors such as leaf curling, rolling, and occlusion lead to inaccuracies when determining parameters from 2-dimensional (2D) images [2]. Determining this information is critical in assessing the overall validity of the crop and identifying potential alterations needed to improve yield.

Reconstruction of plants in 3D has typically been solved through either hardware or software approaches. Hardware systems based on light detection and ranging (LiDAR) use time-of-flight light measurement to accurately measure the distance between the sensor and evenly spaced points within a scene. These devices are capable of highly accurate representations of plants [3]. However, they are often expensive to acquire and require expertise to operate. Lower-cost software-based methods such as structure from motion (SfM) operate by generating a point cloud from a series of 2D images of a plant [4]. Points are triangulated across views to estimate their position in 3D space. Modern SfM approaches are efficient and require little hardware beyond image capture devices. However, these methods often produce sparse representations of a plant and may struggle to capture the fine detail necessary for accurate phenotyping. Both LiDAR and SfM generate point representations of scenes rather than continuous surface representations, which may be required depending on the phenotyping task.

Recent progress in deep learning has led to the development of view synthesis models, which offer exciting new opportunities for 3D plant phenotyping. These models are trained from 2D images of a scene and are commonly used to generate new views of objects not included in the initial training set. However, they can also be used to extract volumetric representations of plants, point clouds, and continuous representations, potentially enabling step change in 3D plant phenotyping.

Neural radiance fields (NeRFs) [5], popularised in 2020, utilise a neural network and volumetric rendering to generate a continuous representation of a scene. Three-dimensional Gaussian splatting (3DGS) [6] projects a series of coloured ellipsoids into a scene and employs gradient descent to optimise their positions, shape, and shading. These methods implicitly generate a 3D representation of a scene, and while most literature focuses on generating unseen views, these techniques can be utilised for 3D reconstruction of plants. There has been limited research on the use of view synthesis models for plant shoot reconstruction; these are emerging technologies, but primarily, there is finite availability of large multiview datasets required to exploit these methods.

In this article, we introduce an extensive multiview dataset of wheat plants and demonstrate the state-of-the-art performance of view synthesis models on both novel view synthesis and 3D plant reconstruction. Our dataset comprises 20 wheat plants captured over 6 time frames. For each plant and at each time point, we train high-quality models using both NeRF and 3DGS approaches, which we use for novel view synthesis and full 3D reconstruction of each plant. Our dataset aims to serve as a baseline for evaluating different view synthesis models on plants and can also be used to develop and test a large number of downstream tasks related to 3D phenotyping, such as extraction of 3D traits, surface reconstruction, canopy light modelling, and next-best-view problems. We provide straightforward scripts and thorough documentation to assist other researchers in executing our trained view synthesis models locally.

We utilise wheat plants in this article as these species are one of the most widely produced crops worldwide, accounting for 20% of human calories as well as providing vital proteins, minerals, and vitamins for a healthy human diet [7]. The global average annual yield increase of wheat is 0.9%, but the predicted increase in demand is 2.4% [8]. Wheat plants offer substantial challenges compared to typical scenes used to evaluate view synthesis models. These include multilayered occlusions and narrow leaf structure, making them an appropriate target for evaluating the capabilities of different 3D reconstruction methods.

Each wheat plant was captured from multiple views using a dual-robot imaging setup, enabling the capture of a wide range of views and good coverage of each plant. Our robot setup also facilitates logging of camera positions in metric units, ensuring that the measurements recorded on the reconstructed plants from either NeRF or 3DGS are equivalent to their real-life counterpart. We use 2 robots to capture the widest possible range of views, but our approaches are compatible with single-robot or other systems.

**Figure 1:**
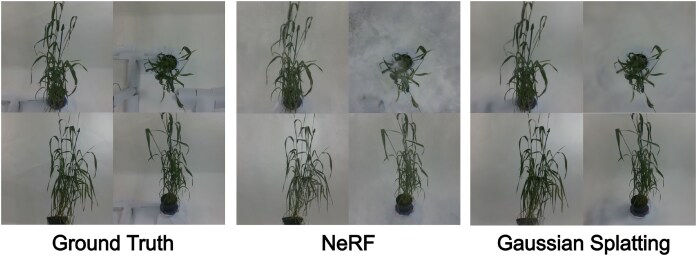
Showcase of some of the rendered images for one of the plants in our view synthesis dataset (bc1_1054: 13-03-24). Left column displays the captured ground-truth images. Middle column contains images rendered after training with standard RGB images, with transforms calculated after bundle adjustment, on the nerfacto NeRF model. Right column displays images rendered after training with undistorted RGB images, with transforms calculated after bundle adjustment, on the splatfacto 3DGS model.

We validate the accuracy of novel view synthesis by comparing rendered images against unseen views of the real plants. We find that both approaches offer excellent render quality, with 3DGS offering the best performance. Figure 1 shows rendered images of a wheat plant that was reconstructed using both of these methods.

To validate the accuracy of the 3D reconstructions produced by both NeRF and 3DGS, we manually capture several of the imaged plants using a handheld structured light scanner (Handheld 3D Scanner; Einstar).We compare our model reconstructions against this ground truth by converting these representations into point clouds and measuring the average distance between model and corresponding ground-truth points. We found that the average error between the reconstruction and ground-truth scan was only 0.74 mm for 3DGS and 1.43 mm for NeRF. In contrast, point clouds generated using multiview stereo (MVS) and SfM had an average error of 2.32 mm and 7.23 mm, respectively.

We conclude by discussing the potential use cases and implications of these new technologies on the field of plant phenotyping. We release the full dataset of 112 plant instances and over 35,000 RDB-D images, all trained models, camera parameters, computed 3D representations, and ground-truth scans. We also release our image capture framework, compatible with any robot that supports the robot operating system (ROS) [9]. This framework can generate new datasets ready for training on any standardised view synthesis model. We also provide our robot configuration files, enabling convenient replication of the setup in any environment. If required, this same setup can be deployed virtually using the Gazebo robotics simulator library [10], enabling the capture of synthetic plant models.

In summary, our main contributions are:

- A new view synthesis dataset of 112 wheat plant instances. This dataset can be used to develop and train new view synthesis and 3D modelling approaches that target complex plant topology or to develop and evaluate new 3D phenotyping approaches. This dataset can be accessed via https://plantimages.nottingham.ac.uk [11] or https://https://doi.org/10.5524/102661 [12].
- A dual-robot image capture setup applicable to a variety of robot manipulators and image capture devices. Our system is designed such that all 3D models exist in a metric coordinate system, and so phenotyping measurements may be directly mapped to the original plants.
- Experiments demonstrating the benefits and drawbacks of view synthesis models compared to standardised methods for 3D plant reconstruction and a detailed comparison of the strengths and weakness of both NeRF and 3DGS approaches for plant phenotyping.
- All of our robot configuration files, view capture pipeline, and 3D Gaussian splatting to point cloud conversion codebase can be found on our GitHub Repository via https://github.com/Lewis-Stuart-11/3D-Plant-View-Synthesis [13].

## Background

### 3D plant representations

Point clouds represent one of the more fundamental forms of 3D representation, wherein an object’s surface is encoded as a set of points with a 3D position and optionally an RGB colour value. This data representation has become popular for downstream phenotyping tasks, such as leaf/stem segmentation [14], or estimating branch angles [15]. Additionally, several software packages have been developed to automatically extract phenotypic traits, such as plant height, projected leaf area, and convex hull volume, from point clouds of various species [16, 17]. Consequently, many 3D plant datasets have been developed that consist of point clouds of plant structures that can be utilised for phenotyping [18]. Despite this, point clouds are often impacted by erroneous outliers, frequently necessitating the application of postprocessing algorithms to denoise the reconstructed data. In addition, point clouds provide no explicit surface representation.

Voxel grids constitute another widely adopted representation method, in which the 3D environment is divided into a grid of voxels, each constituting distinct colour values in a predefined space. This representation has demonstrated its efficacy in various phenotyping tasks, including the assessment of holistic and component characteristics [19], as well as the computation of leaf angles [20]. While voxel grids offer good noise robustness, they often sacrifice fine-grained surface detail when compared to point clouds due to their fixed grid resolution.

Meshes represent an alternative 3D representation approach that involves the reconstruction of plant surfaces through the use of polygons. While meshes have occasionally been utilised for phenotyping [21], their additional complexity often sees their use in physical simulations rather than standardised phenotyping practices.

A drawback common across current 3D representations is that the quality of the reconstruction is reliant on challenging data acquisition and 3D reconstruction methods. Image-based methods often struggle to reconstruct the complex topology of plants, and as such, these 3D structures are often sparse, inadequately capturing the intricacies of their real-life counterparts.

Recent developments in deep learning have led to several new formats for representing 3D structures. One important development has been the adoption of implicit representations, which model plants as a continuous structure, rather than at discrete positions such as points or voxels. Typically, this is achieved using a deep neural network that is trained to represent the plant and sample from any position. These representations circumvent the limitations of traditional 3D structures, as the accuracy of the reconstruction depends solely on the resolution of the input data and the complexity of the reconstructed model. While these models offer potentially unlimited sampling resolution, in practice, they can be challenging to use to extract plant traits. All existing phenotyping pipelines assume a discrete representation in a form above, and further research is required to explore the potential of these exiting new models.

Another recent development has been in 3D Gaussian representations, which are conceptually similar to point clouds. This representation is formed of a series of 3D Gaussian functions projected into 3D space, with their shape and colour being optimised to effectively model the plant. Intuitively, these can be thought of as a coloured or semi-transparent ellipsoids. Many ellipsoids can be positioned and shaped to represent a dense reconstruction of the surfaces in the scene.

Overall, these modern representations circumvent the limitations of traditional 3D structures, as the accuracy of the reconstruction depends more on the resolution of the input data and the complexity of the reconstruction model. We refer interested readers to [22] for a detailed discussion of 3D representations and reconstruction approaches for plants and trees.

### 3D reconstruction methods

Reconstruction methods are typically split into 2 categories: active approaches, in which light emitters are utilised to retrieve information about a 3D scene [23], and passive approaches, in which equipment, typically RGB cameras, are employed to receive light that can be used to extract 3D information of an environment [24]. A common approach to active 3D reconstruction involves the utilisation of 3D laser scanners/LiDAR cameras. These devices determine distances from their optical centres by measuring the time it takes for emitted light to reach a specific point on a surface within an environment. Costly industrial-grade scanners are capable of generating highly detailed 3D point clouds within a defined area [25]. Where cost is prohibitive, low-cost depth cameras have also been utilised for effective plant reconstruction [26]. While these technologies excel in rapid data acquisition, they do have limitations, including restricted coverage and difficulty capturing dense or topologically complex regions. As a result, these scanners are not optimally suited for capturing plants characterised by intricate detail (e.g., thin leaves, small branches, spikes) [27].

Two-view stereo is one of the early forms of passive 3D reconstruction and requires only 2 RGB cameras. Conversion from 2D to 3D involves triangulation of pixel data based on registered camera positions. This process offers rapid and effective retrieval of plant characteristics but typically yields sparse reconstructions of plant models [28].

MVS extends this approach by introducing multiple cameras into the image acquisition process. Consequently, this approach can generate dense 3D point clouds with impressive high point-position accuracy. MVS has been shown to reconstruct plant canopies with high accuracy [29, 30] and has become popular as an initial step in phenotyping pipelines [31, 32]. Nevertheless, this approach can incur a high computational cost compared to active reconstruction methods, and the accuracy of the 3D point cloud is directly reliant on the precision of the registered camera’s position and rotation.

MVS produces dense point clouds, but it does not compute camera poses and so is typically preceded by a camera calibration step such as the use of a SfM algorithm. SfM produces sparse point clouds but can calculate camera poses that are not known prior to image acquisition. SfM incorporates preliminary steps such as point extraction, matching, and triangulation to accurately determine camera positions before proceeding to dense reconstruction. SfM has been shown to work effectively for reconstruction of plant geometry [33] and trees [34]. However, this process requires accurate feature matching, which is challenging on plants where texture is often repetitive, and they exhibit complex shape and self-occlusion. Furthermore, while the process of camera calibration in SfM makes image acquisition more flexible, this commonly results in 3D scenes that do not correspond to real metric or known units. This means that scenes must be manually scaled or otherwise registered later by some additional process. Without such a registration, key phenotyping characteristics such as plant height, leaf area, and convex hull would be inaccurate.

Ultimately, the choice of 3D reconstruction technique depends on the specific plant being captured, the available capture equipment, and the desired processing time [35]. Additional information on various standardised 3D plant reconstruction methods can be found in [36] and [37].

### View synthesis models

View synthesis is the process of generating novel images of an environment from a specific viewpoint not included in the set of prior images. Although view synthesis models have seen limited uptake for plant phenotyping so far, we foresee increased use in the future, better enabling applications such as next best view and extracting phenotypic traits from multiple views. View synthesis models only require a set of 2D images and a series of “transforms,” which define the intrinsic and extrinsic camera parameters, similar to the requirements to generate a point cloud using MVS.

NeRFs [5] are a proposed solution to view synthesis, producing novel views that have been seen to far surpass previous methods, even on complex scenes. NeRF employs volumetric rendering techniques that utilise a neural network to predict density and colour at positions in the environment. Consequently, NeRFs are a promising candidate for 3D reconstruction from images.

Several impressive extensions have been proposed for NeRF, such as improved ray-casting in Mip-NeRF 360 [38, 39] and hash-encoding in Instant-NGP [40]. NeRFStudio, a popular view synthesis framework, introduced NeRFacto, which incorporates successful architectural improvements from various NeRF models [41].

While NeRFs produce extremely impressive reconstruction results, utilising a neural network to encode the entire scene leads to slow rendering times and challenges that arise with handling implicit data.

At the time of writing, there has been limited research utilising NeRFs for 3D plant reconstruction. First, it has been shown that plants can be reconstructed in high accuracy by comparing the NeRF representation to a captured ground-truth scan, yielding an impressive result of only 10-mm error for single indoor maize plant [42]. Other studies have extended this by evaluating NeRF on multiple indoor and outdoor plants [43], confirming similar results, with NeRF representations trained using NeRFacto producing the most precise 3D representations.

It has also been demonstrated that NeRF can reconstruct a variety of different types of fruit with high accuracy [44], including peppers, tomatoes, and pitahaya. This shows that NeRFs are capable of effectively reconstructing plants with complex structures, materials, and occlusions.

Other studies focused more on applying NeRF directly to phenotyping problems. PeanutNeRF [45] accomplished peanut pod detection by creating a 3D implicit representation of the peanut plant using a NeRFacto model and using a segmentation and bounding box estimation pipeline to identify areas in the scene that encapsulate each individual peanut pod. Another study deployed a portable robot with an attached camera and scanner in a greenhouse to reconstruct peppers [46]. A segmentation algorithm was developed to identify these peppers from a trained NeRF model and extract phenotypic traits, such as width and height. These measurements could be accurately calculated since the robot was calibrated in metric units. This study was able to reconstruct the peppers with an high accuracy of 0.881 mm compared to a scanned ground-truth point cloud. While NeRF models are capable of high-quality reconstructions, replicating these results can be challenging, and captured datasets are either limited or have not been made public.

3DGS [6] represents another approach to view synthesis, in which the scene is populated with 3D Gaussian ellipsoids that encode colour and density at different positions within an environment. Gradient descent is used to optimise each of the Gaussians in the scene to fit the environment correctly. Culling algorithms are incorporated to ensure redundant Gaussians are removed from the scene.

**Figure 2:**
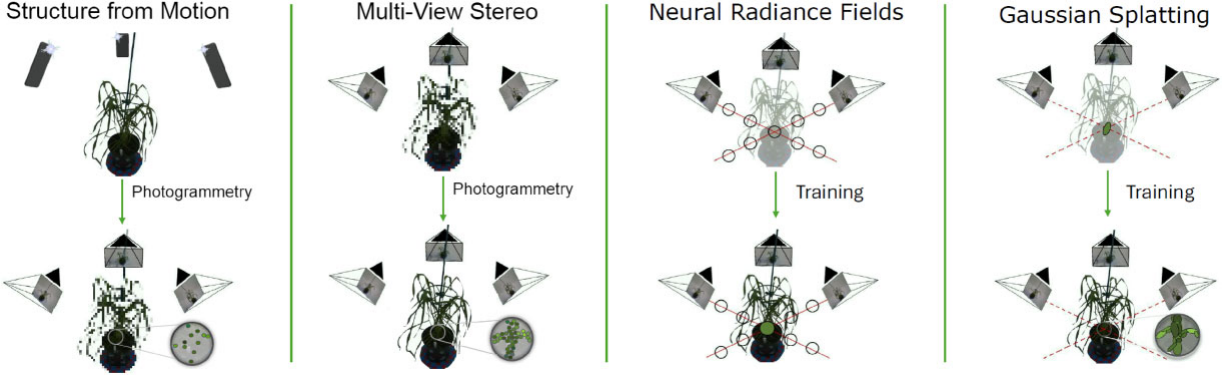
A visual depiction of the basics of SfM, MVS, NeRF, and 3DGS. In SfM, data are captured and a point cloud is generated using photogrammetry. MVS takes the SfM point cloud and camera poses and calculates a much denser point cloud. It is important to note that MVS does not always require an initial sparse point cloud, but it does for COLMAP, which is the framework that was employed for our experiments. Both NeRFs and 3DGS begin with an empty scene and are trained on the captured images with associated camera poses. In NeRFs, ray marching is used to interact with the scene at specific locations, and these queried points are optimised to reconstruct the plant correctly. 3DGS projects a set of initial Gaussian ellipsoids into the scene, and over time, these Gaussians are optimised to better represent the shape and appearance of the plant in 3D space.

There have been several proposed improvements to 3DGS, such as incorporating anchor points [47], improved pruning functions [48], and SfM-free initialisation [49], but so far, the process is still in its infancy. NeRFStudio has released its own 3DGS model known as Splatfacto, which can produce high-quality reconstructions. Unlike NeRF, Gaussians are an explicit representation of the scene, which makes them more flexible to handle, allowing 3DGS applications to perform real-time rendering. The differences between these 2 methods are visualised in Fig. 2.

To our knowledge, there has been no previous application of applying 3DGS to plant shoot reconstruction.

### Plant imaging setups

Numerous plant image capture setups have been proposed for 3D reconstruction, including those noted in the studies above. Some involve gantry systems equipped with robotic arms designed to capture views from various angles around a plant subject [50]. Simpler setups utilise a rotating board to reposition 2 cameras around a plant subject [51], while other systems use a turntable to rotate the plant subject rather than manoeuvring the cameras [32, 52–55]. Many existing installations are challenging to re-deploy into new locations due to a lack of available configuration and software. Others with limited range of movement are incapable of capturing the full range of views required for effective 3D reconstruction using view synthesis models.

Here, we utilise two Universal Robots UR5 robotic arms, along with a turntable, to capture the broad range of necessary views for reconstruction of wheat plants. UR5 robotic arms have found application in various phenotyping contexts, such as leaf scanning [56], plant grasping/pruning [57, 58] and next-best view planning [59], primarily due to ease of use and moderate reach.

## Methods

### Robotic imaging setup

View synthesis models, such as NeRF and 3DGS, benefit from a large number of views of the scene. Ideal imaging setups would capture images at equidistant intervals around an object being imaged, with as much of the object as possible in view within each frame. Our robot capture setup is designed with these features in mind while remaining easily reconfigurable and adaptable to other plant species or installation locations.

We experimented with a single UR5 using an Intel Realsense D435i camera mounted at the tool centre point (TCP). However, we found that a single robot failed to provide adequate reach to obtain the majority of required views, particularly across the full range of 360 degrees around the plant.

To address this limitation, we integrated a Zaber X-RST stepper motor turntable, which offers a full 360° rotation range with 0.16$^{\circ }$ unidirectional accuracy. The turntable’s ability to rotate to any angle allowed us to focus only on viewpoints along the x- and z-axes, with the y-axis being fixed. We set the turntable speed to precisely 3° per second to minimise plant micro-movements during rotation while also maintaining efficient rotation time. The turntable was centred at the origin (0,0,0) of our robot’s coordinate system, allowing straightforward calculations of transform positions relative to the turntable.

**Figure 3:**
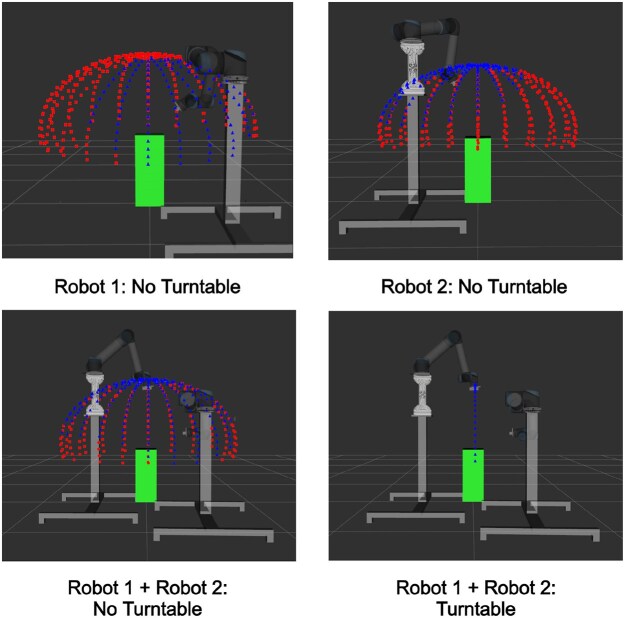
Image showing the effectiveness of the different considered setups. Blue points represent positions around the plant that could be reached, while red points represent positions that were unreachable. These points were generated for a plant with a height of 0.5 m and a capture radius of 0.75 m . The different setups are as follows: (i) A setup consisting of only 1 UR5 arm in position (0.35 m, −0.45 m, 1.3 m). (ii) A setup consisting of only 1 UR5 arm in position (0.85 m, 0.45 m, 0.85 m). (iii) A setup with 2 UR5 arms in positions (0.35 m, −0.45 m, 1.3 m) and (0.85 m, 0.45 m, 0.85 m). (iv) Our final setup with 2 UR5 arms in positions (0.35 m, −0.45 m, 1.3 m) and (0.85 m, 0.45 m, 0.85 m) and an accompanying turntable.

Despite this, we found that some views, particularly those above the plant, remained challenging to reach for a single robot. We therefore employed a second UR5 robotic arm mounted on a separate pedestal, elevated above the base of the other robot, which increased our range of potential views. The base of the first UR5 was positioned at coordinates (0.35 m, −0.45 m, 1.3 m), while the base of the second UR5 was located at coordinates (0.85 m, 0.45 m, 0.85 m). Our coordinate system adhered to the standard ROS convention, where the positive z-axis points upward and values are in metric units. Each UR5 base was mounted on a customised pedestal, strategically positioned to provide access to views ranging from 0.3 to 1.5 m from the turntable origin. Considering that the camera should be roughly 1.5× the distance from the centre of the plant for effective reconstruction, this imaging setup was capable of capturing wheat plants from 0.2 to 1.0 m in height during our experiments. These choices ensured that our setup could capture a wide range of views for a variety of different plant sizes. Each iteration of our setup, along with a showcase of reachable views, is depicted in Fig. 3.

To control the UR5 robotic arms, we installed ROS Noetic Ninjemys and developed a custom dual UR5 MoveIt [60] package, enabling parallel path planning for both arms. To facilitate this, we created a custom Unified Robot Description Format (URDF) file with joints extending from the turntable centre to each camera’s optical centre. Utilising ROS ensured that all generated transforms and robot positions were consistently in metric units. Additionally, we established distinct kinematic chains for each arm to precisely align the plant’s centre with the middle of each captured image, an important factor for accurate 3D reconstruction.

We utilised 2 RealSense D435i cameras for image capture, mounted on the TCP of each UR5 robotic arm. The RealSense cameras were chosen due to their small external dimensions and straightforward integration onto the robot TCPs. These cameras allowed us to acquire precise depth information that could be integrated into the 3D reconstruction process if desired. The depth channel represents an optional addition to any 3D reconstruction pipeline. While higher-quality cameras could have been chosen, producing high-quality reconstructions using standard HD cameras shows the efficacy of view synthesis models for more affordable capture setups. Furthermore, the training time is directly related to the size of the input images, so lower resolutions offer an extra advantage in this regard. We calculate the intrinsic parameters for each camera through a standard calibration process utilising a chessboard pattern and OpenCV’s camera calibration [61] toolkit. These parameters can be combined with the camera pose, provided by the positioning of each robot, to produce a full mapping from 3D world coordinates into each image.

One of the key challenges associated with using a turntable is that, although the plant subject can rotate to any desired angle, the background remains consistent in each view. This is a challenge for view synthesis models, as the discrepancy between the foreground and background introduces significant noise during model training. To address this, we implemented a white background around the robots and turntable, where the lack of notable features increased the quality of the final 3D reconstruction. We also experiment with additional background removal.

Since extracting features from objects in front of a white background can be challenging, a red and blue checkerboard was positioned on the turntable. This assisted in the feature extraction process that was employed in our camera pose refinement process, as well as assisting the point cloud generation for our experiments with both SfM and MVS.

We observe that in some views, the base of the second robot appears in the images captured from the first robot, adding additional noise in the final reconstructions and causing the plant to be rendered incorrectly. We resolved this issue by cropping each image to have a square aspect ratio with a pixel size of 1,080 × 1,080, improving reconstruction quality and reducing training time by half.

Two diffuse light sources were positioned either side of the plant to ensure that lighting would appear uniform, with a minimum of cast shadows and specular reflections, when the plant was 3D reconstructed.

Finally, apart from the turntable, we faithfully replicated our system in a Gazebo simulation environment. This allows the simulation to be run with an associated view capture software package to generate view synthesis datasets on synthetic 3D models. More information about how we calibrated our setup can be found in section 1 of the supplementary material. All robot configuration files and comprehensive documentation can be accessed in our GitHub repository.

### View capturing pipeline

**Figure 4:**
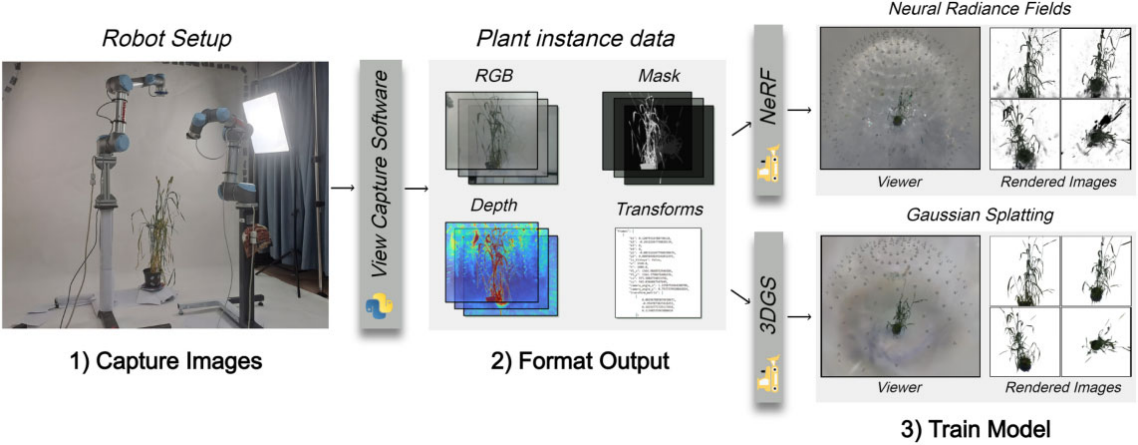
Overview of our entire process covering image capturing to 3D reconstruction. First, a set of images and transforms are captured using the view capture framework. Next, the outputs are formatted. This includes operations such as refining transforms using bundle adjustment and generating masks. Finally, the generated dataset is trained using NeRFStudios’ NeRF and 3DGS models.

We build upon our robotic platform and develop a highly customisable view capture framework capable of generating view synthesis datasets with any ROS-supported robot equipped with a camera and an associated MoveIt package. The framework is designed to capture image datasets with known transforms that can be used to train view synthesis models. This capture pipeline is shown in Fig. 4 .

A full capture run begins using an approximation of the dimensions of the plant, as well as the optimal radius around the object’s centre used for generating the camera positions. Exact dimensions of the plant are not necessary; during our experiments, we typically chose a single value for all plants at the same growth stage. We found that capturing views roughly 1.5× the height of the plant resulted in the best reconstructions, since the majority of the plant is in the camera frame while not being too distant to impact image quality. Using a simple sphere point generation algorithm, potential views are formed around the object, with the turntable rotations being calculated as the angle between the fixed x-axis and the generated points.

We then determined the closest robot to each specified point, adding that point into the respective robot’s movement queue. During execution, each robot attempts to reposition to the next point in the queue in parallel. If this fails, each robot will then attempt to move to each point sequentially. At every point, an RGB-D image is captured alongside the current transform, which accurately maps the camera in 3D space relative to the current angle of the turntable. For each captured image, an associated mask is generated that labels pixels that are part of the plant structure, which can be used for segmenting out the background. A simple pixel intensity threshold algorithm was used to remove pixels in the white background. Erosion and dilation functions are included to remove excess noise. Finally, a connected components process was utilised to identify the largest component in the mask, which we found was the plant in all cases, and other components are removed from the image. This process produced masks that almost completely removed the white background but also occasionally segmented out small stems of the wheat plant. While both depth and segmentation information are useful inclusions to the dataset, these are not essential for training of view synthesis models. We experiment with including these data in the training process.

In typical pipelines, SfM is required to determine the camera poses at each image. Our approach utilising robotics ensures that the camera poses are already known for each image, skipping the SfM stage that could lead to additional errors or inaccuracies. This also provides 3D coordinates in metric units, which is helpful for plant phenotyping problems, and forgoes the need to calibrate camera poses for each plant.

We have found that NeRF models in particular are sensitive to even very small inaccuracies in view position, such as errors in camera position of ∼1 mm. To address this issue, we incorporated bundle adjustment to refine our camera poses once capture was completed. The bundle adjustment process was initialised using the camera poses produced by our system, after which feature extraction, feature matching, and point triangulation across the captured image set refined the position of each camera. Our generated masks were incorporated into the feature extraction process, ensuring that background pixels were excluded during point triangulation, improving the final accuracy of the bundle adjustment step. While our masks do suffer from slight inaccuracies, we found that the impact on the refinement process was negligible, with the majority of the points correctly triangulated. The refinement process was iterated 3 times to ensure accurate convergence, with camera poses being rescaled between iterations to avoid drift of the generated point cloud. Once the camera poses had been optimised, each image was undistorted and a final point cloud was generated, since 3DGS models require these data to train correctly. This process was performed using COLMAP [62], a popular SfM framework.

We have made all software, configuration files, and documentation required to run our image capture pipeline publicly available.

## Experiment

### Data acquisition

To evaluate the efficiency of view synthesis models for plant reconstructions, we captured a large dataset of wheat plant images and associated transforms. Wheat plants were chosen due to their agricultural significance and the challenges they present for 3D reconstruction, such as occluded leaves and thin stem structures.

To ensure that a diverse dataset was captured, 20 individual wheat plants, selected from 6 different genotypes (see below), were imaged at 6 distinct time points. Image capturing began 6 weeks after the plants were sown, and we captured these plants again at 7, 9, 10, 11, and 15 weeks. The plants were divided into 3 batches, with each batch being imaged on different days to maintain consistency. Each batch was transported from the University of Nottingham (UoN) Sutton Bonnington campus, where they were grown, to our imaging centre at the UoN Jubilee campus. On week 7, only 12 of the 20 plants were captured due to a technical issue with one of the UR5s, delaying image capture for 1 week. We include this week regardless as 12 valid instances were captured. Plants were germinated in John Innes No. 2 compost and then vernalised at 6°C with 18 hours light, 6 hours dark for 4 weeks. After vernalisation, plants were potted into 2-L pots with John Innes No. 3 compost and grown in glasshouse conditions. The cultivars used in this study were provided by the UoN Wheat Research Centre and the John Innes Centre Germplasm Resource Unit; they are as follows: Chinese Spring, Langdon, BC1(1051-1054), GRU-2B(2J), GRU-2D(2J), and GRU-DA5J.

Chinese Spring is an elite cultivar of hexaploid bread wheat, *Triticum aestivum* (2n = 6x = 42 (AABBDD)). Langdon is an elite cultivar of tetraploid durum wheat, *Triticum turgidum* (2n = 4x = 28 (AABB)). BC1 plants are from an original cross of Chinese Spring × *Aegilops mutica* (2n = 2x = 14 (TT)), creating the first filial generation, and subsequently backcrossed to Chinese Spring, creating a BC1 introgression line. GRU-2B(2J) and GRU-2D(2J) are *T. aestivum* with a chromosome substitution from *Thinopyrum bessarabicum* (2n = 2x = (JJ)), and GRU-DA5J are where a disomic additional chromosome of *Th. bessarabicum* is present.

These cultivars were selected based on their genetic variability. Bread wheat, durum wheat, and wild relative substitution, additions, and introgression lines all express varying phenotypes. This broad range of different wheat plant ensures that our dataset is extensive and provides additional challenges for downstream tasks.

To ensure consistent alignment, a crosshair icon was attached to the pot of each plant. This enabled us to position the plant in a similar pose and orientation for each capture session, potentially facilitating growth tracking over time.

During capture, approximately 320 RGB-D images were taken around each plant at equidistant intervals from the centre of the main stem. This number was chosen to balance reconstruction quality and capture time. A 1:8 ratio was used for our training/evaluation images, which ensured that our evaluation results correctly reflect the accuracy of the final reconstruction while also ensuring that sufficient images were utilised in the training process. The entire imaging process, including postcapture bundle adjustment, took approximately 30 minutes for each plant.

On the 11th week, we captured a ground-truth scan of each plant using an Einstar 3D Handheld Portable Scanner. This scan provided a precise ground-truth 3D point cloud, allowing direct comparisons between the scans and model reconstructions. To validate the scanner’s capability in generating a consistent ground-truth point cloud, we generated 5 scans of a metal plant model and assessed the consistency of generated points between the resulting point clouds. We found that the average distance between corresponding points across repeated scans was 0.76 mm, demonstrating a high degree of repeatability. We also compared each of the generated scan point clouds against a reference point cloud produced by an X-ray microCT scanner (Model v|tome|x L; GE Healthcare) with a spatial resolution of 150 µm. The averaged accuracy difference was 0.75 mm, suggesting that the Einstar is suitable for providing accurate ground truth for our experiments.

### Training

For each plant, we trained several variations of our captured data, as shown in Table 1. Our aim was to find the combination of image, transform, and model that produced the best reconstruction results for both NeRF and 3DGS.

**Table 1:** The different combinations of input images, transforms. and models used for the various training setups

	Transform type	Training images	Model
1	Original	RGB	NeRFacto
2	Refined	RGB	NeRFacto
3	Refined	Segmented RGB	NeRFacto
4	Refined	RGB + depth map	Depth-NeRFacto
5	Refined	Undistored	Splatfacto
6	Refined	Segmented undistored	Splatfacto

We trained using both original transforms and those refined via bundle adjustment. Next, models were trained using the standard RGB images with backgrounds, and others were trained using the segmented images with the background removed. Depth maps were also included when training the NeRF model; currently, this is not supported in the 3DGS model. The 3DGS models were trained on undistorted images following bundle adjustment, as well as using the initial sparse point cloud produced by this process.

Two variants of NeRF models were trained, NeRFacto and Depth-NeRFacto, depending on whether an experiment utilised the depth information provided with each image. Each NeRF model was trained for 30,000 iterations, after which we observed no further improvement in performance. All models were trained using the Adam optimiser and a batch size of 4,096. We used an initial learning rate of $1\times 10^-2$ reducing to $1\times 10^-4$ over the training process. After training each model, the final iteration was used to evaluate testing performance. Each NeRF model was also converted into a point cloud and mesh using NeRFStudio.

For Gaussian splatting, we utilised the Splatfacto model. Each was trained with a minimum alpha threshold of $5\times 10^-3$, a scale threshold of 0.5 mm, and a spherical harmonic degree of 3. Gaussians were initialised using the sparse point cloud generated using COLMAP during camera refinement process. We used the default learning rates for Splatfacto, which vary across the parameters such as mean, scale, orientation, and spherical harmonic features. At the time of writing, no standard techniques were available to convert Gaussian splatting data into dense point clouds for analysis. Selecting only the centre positions of each Gaussian would produce a point cloud that was too sparse for an effective comparison with the ground truth. We therefore developed a new approach for this task.

Our framework generates point clouds from Gaussian scenes by fixing the total number of points required and distributing these appropriately across all Gaussians in a scene based on their relative size. Thus, larger Gaussians generated more points. All points were sampled randomly from a Multivariate Normal distribution based on the 3D covariance matrix of each Gaussian. Point colours were derived by rendering images across the dataset for that scene and tracking the contribution of each Gaussian to the final pixel colour at each camera location. Each Gaussian was coloured based on the pixel across the rendered images to which it contributed the most colour. This strategy prevents points with low pixel colour contributions or high transparency being assigned erroneous colours that do not represent the final rendered scene. Our implementation produces accurate results and offers high customisation to support a variety of different scenes.

We incorporated several techniques for generating the point clouds for both NeRF and 3DGS that ensured that the entire plant structure was represented entirely. First, we cropped the generated point cloud using an axis-aligned bounding box to ensure that the background was not included in the point generation process. We set the bounding box size to 1 × 1 × 1.5 m and set the centre of this box to the origin of the scene. For 3DGS point clouds, we set specific parameters during point generation to ensure that the points best fit the reconstructed Gaussian. Points that had a Mahalanobis distance greater than 2.5 standard deviation (SD) from their Gaussian centre were removed and regenerated. Gaussians with an opacity less than 1% were culled, and Gaussians with a volume in the top 2.5% of all Gaussian sizes were removed since we observed these Gaussians were always part of the background.

Each reconstruction was cleaned using a set of common automatic operations. First, a statistical outlier removal algorithm was implemented that grouped neighbouring points together, and then any point that lay a distance further than 1 SD from the local group was removed. Next, a noise filter was used that fit an approximate surface across all points and removed points further than 1 SD from the predicted surface. Points were then clustered, and groups of points with fewer than 2,000 connected points were rejected. Finally, we manually segmented out the points located on the pot for both the ground-truth and reconstructed point clouds, ensuring that our accuracy metrics only contained components of the plant relevant for phenotyping. The majority of these operations can be automated, such that most of the points that were part of the true plant reconstruction were included in generating our accuracy metrics.

All NeRFacto, Depth-Nerfacto, and Splatfacto models were trained using a single Nvidia Geforce RTX 2080 Ti graphics card. Alongside the trained models and exported point clouds, we also rendered a set of evaluation images to provide visual comparisons between the ground-truth images and the trained models. These rendered images were used to generate the evaluation metrics for each plant. When rendering the evaluation images for the Splatfacto model, we added a near clip of 0.25 m into the rendering pipeline, ensuring that Gaussians part of the background behind the camera did not occlude the plant. For NeRF, we set near and far ray clipping values of 0.01 m and 5.0 m, respectively, avoiding reconstruction of spurious areas either very close or far from camera positions.

Given that 3DGS models generate a set of Gaussians distributed in 3D space, we are able to perform post-training editing of the reconstructed scene. To remove the background Gaussians, we culled Gausssians with a volume larger than 2.5 mm, and implemented a bounding box with a size of 1 × 1 × 1.5 m to isolate the foreground plant region. The bounding box process removed the majority of background Gaussians.

We then employed a K-nearest neighbours approach to enhance the precision of background removal. For each Gaussian, we calculated the distances to its 15 nearest neighbours and determined the average of these distances. Gaussians with an average distance exceeding 3.5 cm were deemed to be outside the group associated with the plant and were subsequently removed, since Gaussians part of the plant structure are closely compact. Removing large Gaussians rarefied the remaining background, which assisted in identifying outliers using this method.

This process proved to be fast and efficient, successfully eliminating the majority of background Gaussians while preserving the integrity of the plant’s structure.

Each of these view synthesis models can be executed via a Python script available in our dataset repository. This supports launching the models in NeRFStudio to view the reconstructed plants in 3D, as well as training new datasets on these models. A README file is also included that provides more information.

## Results

### Render quality

We evaluate the effectiveness of each reconstruction approach using several metrics. Each metric compares the rendered evaluation image to the ground-truth images but focuses on highlighting different of types of inconsistencies between images.

NeRFStudio offers scripts that automatically generate the following metrics for the evaluation images:

1. **Peak Signal-to-Noise Ratio (PSNR)**: Measures the difference in the intensity of corresponding pixel values using the mean squared error formula. Higher PSNR values indicate lower distortion, with approximately values of 40 db representing an image that is identical to the ground truth. PSNR values are logarithmic and thus represented using the decibel scale (db).
2. **Structural Similarity Index (SSIM)**: Compares local patterns of pixel intensities normalised for factors such as luminance and contrast. Values range from −1 to 1, with 1 representing 2 identical images.
3. **Learned Perceptual Image Patch Similarity (LPIPS)**: Calculates the perceptual similarities between 2 images by comparing the activations after passing through layers of a pretrained convolution neural network (CNN). Lower values indicate higher perceptual similarities.

While these metrics are effective at measuring the similarity between the rendered image and ground truth, they consider the entire image, including the white background. This inclusion can overestimate the quality of the final render, where the simple background represents a high proportion of the image, and is comparatively simple to render.

We introduce a PSNR masked metric to avoid this problem. This metric is based on the PSNR formula but only includes pixels within the generated image mask. This approach provides a more accurate assessment of the effectiveness of the reconstruction on the plant itself. It is important to note that this metric relies on the accuracy of the input mask. This metric can be considered alongside standard PSNR, which incorporates a measure of background quality.

**Figure 5:**
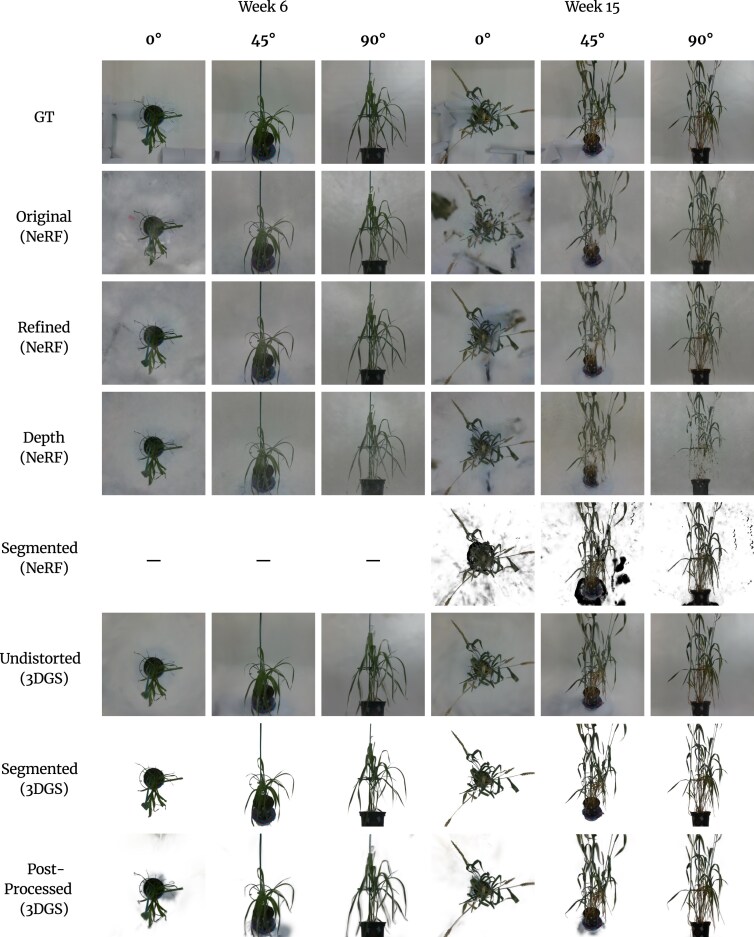
Visual comparison between the reconstruction results for plant bc1_1051 over 2 different time points. The top row of images are the ground truth, not included in the training images, and the images below are the rendered images for each of the different training configurations. The NeRF model with segmented data did not train and produce a valid 3D reconstruction in week 6, which is why there are no rendered images. Rendered images for the rest of the weeks for this plant be found in section 2 of the supplementary material.

The following section is split into a set of experiments, each examining the effectiveness of each of our trained model types. Each of these results are averaged over all 112 trained plant instances. Our goal is to identify the training data configuration that produces the best results for both NeRF and 3DGS. The list of results for all 112 plants can be found in section 3 of the supplementary material. Figure 5 presents a comparison of rendered images for each of the following training configurations.

#### The effect of bundle adjustment on camera accuracy

First, we evaluate the impact of bundle adjustment on the accuracy of 3D reconstructions using RGB images. We compare the original transforms generated via our robot setup to those refined by the bundle adjustment process.

Table 2 shows that, as expected, the bundle adjustment process improved the PSNR by approximately 2.5 db. This shows the importance of extremely precise transform positions for these modern 3D reconstruction processes. As a result, we decided to utilise the refined transforms for all subsequent models, since they produce stronger results compared to the original transforms. We only conducted this comparison on NeRF models, as 3DGS models require the sparse point cloud initialisation after bundle adjustment.

**Table 2:** Evaluation results for NeRF models trained on original transforms acquired from our setup and transforms calculated using bundle adjustment. Both models were trained on RGB images.

Training type	PSNR $\uparrow$	SSIM $\uparrow$	LPIPS $\downarrow$	PSNR masked $\uparrow$
Original (NeRF)	21.28	0.80	0.28	15.29
Refined (NeRF)	**23.90**	**0.86**	**0.22**	**19.49**

#### The impact of depth on synthetic view quality

We examined the impact of including depth maps during model training. We performed these experiments using the NeRF models, as the 3DGS model does not currently support depth maps.

Perhaps counterintuitively, the inclusion of depth maps produced a slightly poorer final plant reconstruction, as highlighted by the PSNR masked values in Table 3. The lower performance of RGB-D is caused by lower render quality on thin individual leaf tips. It is likely that the depth maps were not sufficiently accurate to properly reconstruct the thin structures prevalent in plant shoots. Furthermore, the depth map resolution of 720 × 720 is lower than the RGB image resolution of 1,080 × 1,080, a typical restriction of RGB-D cameras. An additional advantage of using only RGB images is that future experiments based on our system are not required to include depth cameras. We therefore do not consider RGB-D for any further experiments.

**Table 3:** Evaluation results for NeRF models trained on RGB images and RGB images with depth maps.

Training type	PSNR $\uparrow$	SSIM $\uparrow$	LPIPS $\downarrow$	PSNR masked $\uparrow$
RGB (NeRF)	23.90	0.86	**0.22**	**19.49**
RGB-D (NeRF)	**23.95**	**0.87**	**0.22**	18.15

#### The effect of background removal on synthetic view quality

We explored the incorporation of background removal as a preprocessing strategy to enhance render quality. NeRF and 3DGS models are designed to reconstruct the entire scene, including elements irrelevant to the target plant. Consequently, the final 3D representation can generate a white sphere around the plant during reconstruction. This obstructs views captured from outside this sphere, obscuring the plant.

The NeRF training process was adapted to produce no density or colour in areas of background. Similarly for 3DGS, the training process was restricted to only generate Gaussians that appear in the mask foreground, preventing reconstruction of the background. This adapts the training process of 3DGS, but we also implement our postprocessing Gaussian removal process to eliminate the background Gaussians generated during training on unsegmented images.

The results, shown in Table 4, were evaluated using the PSNR-masked metric to focus the metric on foreground regions. It is important to note that this metric is not entirely accurate due to the presence of noise in the masks themselves, which introduces penalties that do not reflect the efficiency of the background removal methods. Nevertheless, the metric offers improved insight over whole-image PSNR.

**Table 4:** Evaluation results for NeRF and 3DGS models using either masked or full RGB images. The 3DGS results for post-training background removal are also included.

Training type	PSNR masked $\uparrow$
RGB (NeRF)	**19.49**
Segmented (NeRF)	6.46
Undistorted (3DGS)	**26.31**
Segmented (3DGS)	13.75
Postprocessed (3DGS)	17.87

Our findings indicate that models trained on segmented images generally produced less accurate reconstructions compared to those trained on unsegmented images. In particular, NeRF often failed to converge when trained using segmented images. These techniques are already reliant on highly accurate camera positions; the addition of potentially imperfect segmentation masks can compound this loss in accuracy. We experimented with various segmentation methods, including CNN-based approaches, but none demonstrated sufficient accuracy to overcome this barrier. These methods also added additional complexity to the reconstruction pipeline. The inclusion of masks did confine computation of the scene reconstruction to pixels relevant to the plant, which reduced training time for both NeRF and 3DGS.

In contrast, the postprocessing Gaussian removal technique proved more effective, with accurate elimination of the majority of background Gaussians. This method was straightforward to implement and integrate into the pipeline. Some small issues remain, such as compact groups of background Gaussians persisting near the base of the turntable or around the top of the plant. These limitations suggest that the process would benefit from incorporating more advanced background removal algorithms in the future.

#### A comparison of robot-derived and SfM calculated camera poses

To compare our image capture setup to standard SfM, we trained the models on transforms generated entirely using COLMAP’s SfM functionality, which is a common approach to calibration and reconstruction across image datasets with unknown camera poses. During the feature extraction process, our generated masks were utilised to ensure that only points on the plant were extracted and matched, facilitating accurate point cloud reconstruction.

For each set of SfM-generated transforms, we calculated an absolute trajectory error (ATE) by aligning the world coordinate systems between our robot camera and SfM camera poses. We then calculated the euclidean distance between corresponding camera poses in each system. If the ATE was greater than 1.5 mm, then it was determined that the SfM process failed to converge correctly, with only 12 of 20 SfM reconstructions meeting this criterion. To ensure a fair comparison between models trained on the robot-derived transforms and SfM-generated transforms, we only included results from SfM transforms that had an ATE less than 1.5 mm, as other results were much less accurate.

As seen in Table 5, our pipeline achieves higher accuracy over a traditional SfM approach. While SfM uses the same bundle adjustment process as our refinement step, SfM must determine the initial camera poses during the sparse point cloud reconstruction process, whereas our approach leverages accurately known robot position data. Consequently, the SfM process often failed to calculate correct positions across all images, only identifying camera positions for an average of 265 of 320 images per plant scene. This inconsistency directly affected the quality of reconstructions, as failed images could not be incorporated into the reconstruction process. Furthermore, only 12 of 20 SfM reconstructions produced camera poses within 1 mm of our robot-derived transforms, implying that SfM may not be a reliable tool for calculating camera poses for indoor plant capturing environments, such as ours.

**Table 5:** Evaluation results for NeRF and 3DGS models. One set was trained using our transforms acquired from the robot setup and bundle adjustment. Another was trained using transforms acquired from SfM. Only results trained on the generated SfM camera poses with an average error less than 1.5 mm were included. Both were trained using RGB images.

Training type	PSNR $\uparrow$	SSIM $\uparrow$	LPIPS $\downarrow$	PSNR masked $\uparrow$
Ours (NeRF)	**23.90**	**0.86**	**0.22**	**19.49**
SfM (NeRF)	21.99	0.82	0.31	17.42
Ours (3DGS)	**28.17**	**0.95**	**0.15**	**26.31**
SfM (3DGS)	26.43	0.93	0.2	21.89

#### Synthetic view quality of NeRF and 3DGS

We compare the performance of the 3DGS model to the NeRF model for rendering new synthetic views of each plant, using our complete robot-based turntable system and refined camera positions. The results in Table 6 show the 3DGS model produced higher-quality synthetic views compared to the NeRF model. From visual observations, there was reduced noise in the 3DGS reconstruction, particularly with view points above the plant. We hypothesise that this is due to 3DGS being more effective at resolving inconsistent background appearance in top-down views, where the robot pedestals are visible. It also seemed that the NeRF models struggled more when handling thin structures on the plant, while the 3DGS models appear to reconstruct these features more effectively. Gaussians on thin structures naturally elongate and align along the direction of that object, potentially offering a more appropriate representation of these shapes.

**Table 6:** Evaluation results for NeRF and 3DGS models. Both were trained on the original RGB images and transforms calculated using bundle adjustment.

Training type	PSNR $\uparrow$	SSIM $\uparrow$	LPIPS $\downarrow$	PSNR masked $\uparrow$
NeRF	23.90	0.86	0.22	19.49
3DGS	**28.17**	**0.95**	**0.15**	**26.31**

### Reconstruction accuracy

Whilst rendering new images of the captured plant is useful, the accuracy of the final plant reconstruction is crucial for extraction of correct phenotypic traits in 3D. We compared point clouds created from each model against our captured ground truths. We employed CloudCompare, an open-source project designed for handling 3D point clouds, to calculate a final accuracy metric [63]. We used the provided average point distance functionality to perform this comparison. To provide a more comprehensive comparison, we include average measures of distance from model points to the ground truth and in the reverse direction from the ground truth to the nearest model points. The first comparison aims to evaluate the similarity of the entire ground-truth scan structure to the reconstructed point cloud, while the other evaluates the accuracy of each reconstructed point, regardless of the sparsity. The standard deviation of each mean distance represents the consistency of the accuracy for reconstructed points compared to the ground truth. A larger standard deviation typically implies that more areas of the reconstruction were underrepresented in the point cloud.

It should be noted that the ground-truth point cloud often failed to capture the thin structures of the plant, which is why the results have a higher inaccuracy value for comparison of the model points to the ground truth.

We first generated a point cloud representation for the final NeRF and 3DGS models. It is important to note that, since both NeRF and 3DGS are dense data structures, there is no limit to the number of points that can be generated by each representation. We chose to generate exactly 10,000,000 points, which ensured our point clouds were dense enough for an accurate comparison against the ground truth. Each point cloud had approximately 7,500,000 points after performing the noise removal operations.

Each reconstruction point cloud was registered and aligned with the scanned ground-truth point cloud via the Iterative Closest Point algorithm. Since the camera positions were captured using our robot setup in metric units, all performance measurements are calculated in millimetres.

To compare the accuracy against other reconstruction techniques, we also generated a sparse point cloud using SfM and a dense point cloud using MVS. We utilised COLMAP to generate these point clouds using the same camera poses captured by our robot setup after refinement. For feature extraction, our masks were included to ensure that the background was not included during feature matching.

We utilised the same noise removal process as described above for MVS since the point cloud had a similar level of noise as the generated 3DGS and NeRF point clouds. For the SfM point clouds, we applied the same noise filter as with the 3DGS and NeRF point clouds, but we then manually removed certain groups of points part of the background. We found that automating the noise removal on SfM points often degraded the quality of the model.

SfM generated an average of 16,760 number of points for all plants that were compared with the ground truth, which were reduced to 16,150 number of points after noise removal. MVS generated an average of 1,650,000 number of points for all plants that were compared with the ground truth, which were reduced to 1,215,000 number of points after noise removal.

The final results are presented in Table 7.

**Table 7:** The accuracy (mean) and variability (SD) of each of the 3D reconstruction methods. The top section of the table is calculated as the average distance from each ground-truth point to the nearest neighbouring point in the reconstructed point cloud. The bottom section of the table shows the average distance from each reconstructed point to the nearest point on the ground truth. A low mean distance implies that the reconstruction was accurate, while a lower standard deviation indicates that the accuracy was consistent for different areas of the plant.

Comparison method	Mean distance (mm)	SD (mm)
GT $\rightarrow$ NeRF	1.43	3.63
GT $\rightarrow$ 3DGS	0.74	0.72
GT $\rightarrow$ MVS	1.31	1.04
GT $\rightarrow$ SfM	6.77	4.34
NeRF $\rightarrow$ GT	4.75	7.42
3DGS $\rightarrow$ GT	4.99	9.56
MVS $\rightarrow$ GT	7.08	12.31
SfM $\rightarrow$ GT	11.43	14.45

Alongside numerical results, we also report the average training times for both NeRF and 3DGS models, as well as the time taken for SfM and MVS to generate a completed point cloud. It is important to note that times required for determining the camera poses for MVS, NeRF, and 3DGS have been omitted. Typically, SfM is required to determine the camera poses; in our case, we utilised our robot setup to determine the camera poses alongside the image capturing. The time taken to capture the images and optimise camera poses is comparable to SfM.

We also determine the total file sizes generated by each of the models, including the neural network for the NeRF representation, the generated.ply file for 3DGS, and the generated COLMAP files for SfM and MVS. Finally, we record the average frames per second (FPS) achieved when generating new 2K resolution images for both NeRF and 3DGS in the NeRFStudio real-time viewer. These results are shown in Table 8.

**Table 8:** Training and rendering results for the different reconstruction models across the 20 tested reconstructed plants. The file size results were the splat file for 3DGS, the weights of the NerFacto neural network for NeRF, and the generated COLMAP files for MVS and SfM. The compute time was rounded to the nearest minute. The render times were calculated as the average FPS for rendering new 2K images in the NeRFStudio real-time viewer.

Reconstruction type	Compute time (minutes)	Rendering time (FPS)	File size (GB)
3DGS	15	15	0.049
NeRF	22	0.2	0.172
MVS	128	N/A	11.683
SfM	11	N/A	0.048

**Figure 6:**
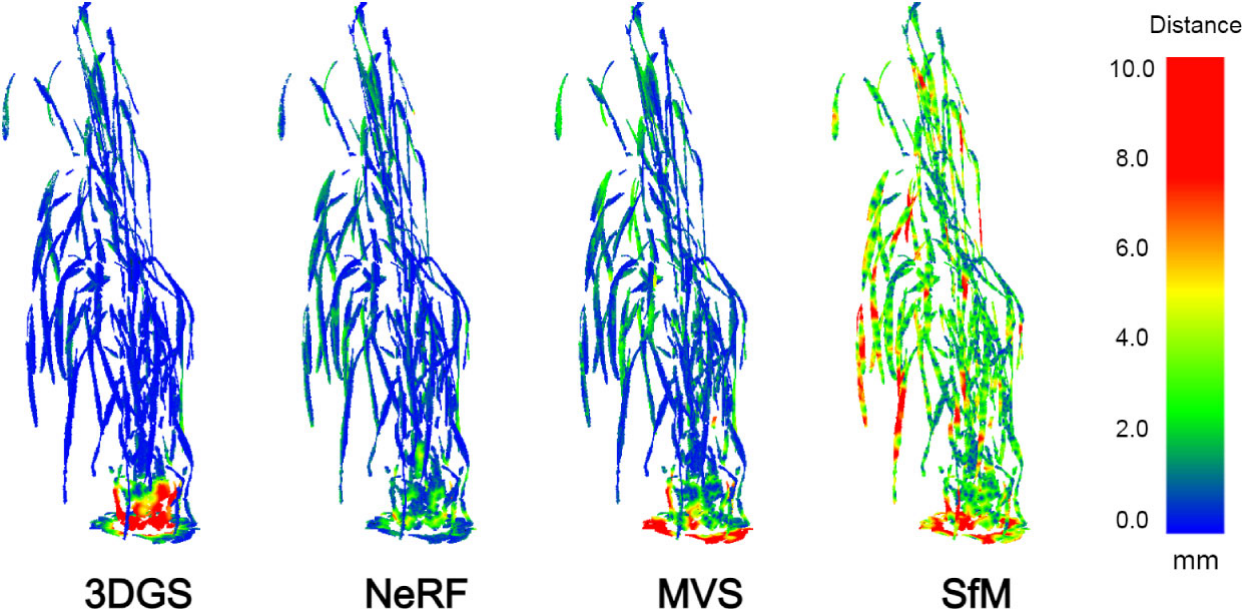
Comparison between the distance error between the ground truth and each of the NeRF, 3DGS, MVS, and SfM produced point clouds for the langdon_3 plant on week 11. On the right is the colour map key, with blue indicating a low distance error and red indicating a high distance error. The displayed pot and turntable of the ground-truth point clouds were not included in the calculation of the reconstruction accuracy metrics in Table 7. We include them here to highlight the effectiveness of how each each method handles different types of surface.

3DGS produced a more accurate ground-truth to point cloud reconstruction accuracy when compared to NeRF. We believe that this is because 3DGS is able to produce a larger set of points around thin structures of the plant, such as the stems, most likely due to the dense population of Gaussians in these areas. The Gaussians can adapt shape and position to better fit different plant structures. However, both methods produced similar results when the point clouds were compared to the ground truth, implying that each method produces accurate points consistent with the underlying 3D plant representation. NeRF typically produced point clouds with reduced noise, and these point clouds may better represent larger surfaces. This is visualised in Fig. 6, in which 3DGS had a higher accuracy around the thin stems of the plant but struggled with larger areas, such as the pot. However, we excluded the pot when calculating the overall accuracy metrics. A table of results with the pot included can be found in section 3 of the supplementary material. When considering the entirety of the scenes, we found that 3DGS had a higher average accuracy than NeRF, suggesting that 3DGS is better for generating more precise 3D representations.

In addition to this, 3DGS offers several advantages over NeRF, training approximately 1.5× faster while also producing rendering results 75× times quicker. The file size of the 3DGS scene was less than the size of the neural network used for encoding the NeRF scene. However, it is important to note that the neural network size is fixed regardless of the scene size or complexity, and so for larger scenes, the neural network may be the better option if file size is a concern. Despite some differences, both approaches are suitable candidates for effective 3D reconstruction, offering similarly effective representations.

Our 3DGS to point cloud approach, and NeRFStudio’s point cloud generation algorithms, take different approaches to creating the final point cloud. Hence, using alternative point cloud generation algorithms for NeRF and 3DGS could produce different accuracy results compared to the results in Table 7.

Compared to SfM and MVS, 3DGS produced more accurate point cloud reconstructions, while NeRF produced more accurate reconstructions only when the point cloud was compared against the ground truth. We believe this is because NeRF struggles to produce a dense number of points around the thin stems of the plant, which is when the standard deviation was high. However, the accuracy of the points generated from NeRF was higher than the 3DGS, MVS, and SfM point clouds, implying that the points around the thin structures were less prone to noise. The accuracy metrics for NeRF were more comparable with that from 3DGS while surpassing MVS and SfM if the pot was included for all point clouds.

View synthesis models are particularly effective at representing detailed structures on the plants, including complex leaf shape, topology, and self-occlusion. Visualised results may be found in Fig. 6. NeRFs and 3DGS can reconstruct scenes as they appear in each camera view, including areas of low texture. SfM, in contrast, must extract, match, and triangulate each point between images accurately, which we find causes additional noise on narrow plant structures that have low contrast and texture. SfM generated a sparser point cloud than the other approaches, particularly on the stems of the plant, negatively impacting the final reconstruction accuracy. SfM has similar compute time and file sizes to 3DGS. MVS produced more dense and accurate point clouds compared to SfM. MVS builds upon the acquired camera poses provided to it and applies more thorough reconstruction steps that aim to extract and project points, for example, patch matching. However, while the reconstructions represent the original plant well, the overall point cloud was slightly less accurate than NeRF and much less accurate than 3DGS, taking into account both comparisons metrics. We believe this is because MVS projects only as many pixels as are present in the image set that can be accurately identified during feature extraction and then triangulated onto the plant. Meanwhile, view synthesis methods offer representations that are not constrained to matching pixels between views.

While MVS reconstructed a denser point cloud than SfM, it required additional computational time to process. MVS took approximately 9× longer to complete than 3DGS. In addition, the total file sizes of the generated MVS Colmap configuration were over 230× larger than 3DGS. MVS applies the same processing per image, meaning that the required compute time and file sizes increase linearly with the number of supplied training images. In contrast, 3DGS and NeRF use a set number of iterations, and so training times will be quite consistent between scenes with varying numbers of images.

## Discussion

Reconstruction of plant shoots in 3D has remained a substantial challenge for many years. We have shown here that both NeRF and 3DGS exhibit remarkable capabilities in reconstructing plants with diverse physical characteristics and complex topology. These approaches rival traditional standardised 3D reconstruction techniques and often provide higher accuracy over common approaches such as SfM and MVS. When used for view synthesis, these models can provide new high-quality synthetic images of plants from views that have not been captured in the original dataset, potentially driving new research in active phenotyping using robotic manipulators and improving our ability to capture phenotypic traits in the presence of substantial occlusion.

To date, there has been limited work using new view synthesis methods on plants. Of those that exist, our results are comparable to other captured plant view synthesis datasets. In [43], various single indoor crop plants were reconstructed using NeRFacto. It was found that the average PSNR for a set single indoor corn scenes was 22.24 db, while the average PSNR for captured plant instances was 23.93 db and 19.47 db for our masked PSNR metric.

In [46], a comparison was made against a ground-truth scan of a series of pepper plants, with the error between each generated NeRF point cloud and the ground-truth scan ranging between 0.865 mm and 0.909 mm. This error is slightly lower than our reported average accuracy of 1.43 mm. It may be challenging to compare results presented on very different species and scenes. However, these metrics still indicate that our results are similar to other recent plant reconstructions and show that view synthesis models have broad applicability across species and scene configurations.

NeRF and 3DGS models offer 2 different approaches that, while superficially similar, are quite different. NeRF models train a neural network to generate an implicit scene representation, where ray-marching is then used to sample colour and density from this space. This approach has some notable advantages: models are continuous representations, allowing us to sample higher-resolution images by simply casting more rays into the scene, at the cost of longer render times. The neural models predict not only colour but also the opacity of material in 3D space, allowing them to be easily converted into volumetric representations such as voxel grids or 3D representations such as meshes. Utilising a neural network means that an entire scene, regardless of the number of images or scale, can be encoded with a consistent file size of roughly 172 MB for a NeRFacto model, as depicted in Table 8. These allow NeRF to be used as part of phenotyping pipelines that leverage these representations, with potentially higher accuracy than previous reconstruction methods.

3DGS instead represents the scene as a series of 3D coloured ellipsoids. This representation is closer to a traditional point cloud representation, but where each point has additional parameters governing shape and colour. Our results in Table 6 show that 3DGS is capable of extremely high-quality view synthesis, often outperforming NeRF on this task. Since the representation is held as discrete points, noise and background removal is comparatively straightforward, which we have demonstrated. However, the number of Gaussians needed to reconstruct a scene can vary depending on the complexity of the training data, meaning that large and complex scenes can produce a file size larger than 1 GB. Despite this, 3DGS offers efficient rasterisation, generating new views almost instantly and comfortably at >60 FPS on a modern desktop PC for standard HD images. This is compared against a NeRF model, where volumetric ray-marching will take approximately 2 seconds per image to render.

The training times for both NeRF and 3DGS are comparable, with each plant instance requiring approximately 15 minutes for 3DGS and 22 minutes for NeRF. Variants of these models exist, such as Instant-NGP [40] and InstantSplat [49], which reduce the time required to train, but these often reduce render quality, and we have focused here on the maximum quality possible as a demonstration of the technology. Both NeRF and 3DGS are active areas of research, and it is likely that some limitations of these approaches will be addressed over the coming years. Our plant dataset provides a new test environment in which to evaluate new developments in these approaches, and improving NeRF and 3DGS for plants specifically, perhaps by targeting methods to improve performance on thin structures or heavily occluded regions, represents a promising area for future work.

### Comparison of view synthesis models and traditional 3D reconstruction techniques

View synthesis models such as 3DGS and NeRF present several compelling advantages over traditional 3D reconstruction methods such as SfM and MVS. As detailed earlier, 3DGS and NeRF produce 3D representations that surpass the accuracy of sparse reconstruction methods such as SfM. While their performance is more comparable to dense reconstruction methods like MVS, 3DGS and NeRF still produced more precise and detailed point clouds in our experiments. Accuracy is an important consideration in selecting the appropriate 3D reconstruction method, since erroneous points may hinder the effectiveness of downstream tasks that depend on precise plant geometry.

A key factor behind the accuracy of the NeRF and 3DGS point clouds is their ability to sample much denser representations. NeRF holds the scene in an implicit continuous representation, permitting sampling of any number of points at any resolution. In a similar way, 3DGS represents the scene using ellipsoids that have quantifiable dimensions, from which any number of points can be sampled. In addition to accuracy, 3DGS and NeRF offer efficiency in terms of file size and computational demands. As shown in Table 8, both methods produced smaller file sizes with faster training times compared to MVS. Despite these promising results, there still exist notable challenges associated with view synthesis models. While Gaussian splats and neural network approaches provide high-quality renderings, these representations are more complex to handle and manipulate than traditional point clouds. There is currently limited support for these representations in the context of 3D phenotyping, where simpler point cloud-based approaches are more commonly used. We theorise that 3DGS will be more useful for phenotyping problems such as segmentation of different key structures of the plant, as part of phenotyping pipelines that derive quantitative measurements. Meanwhile, we predict that NeRF will be employed for situations where its continuous nature can be leveraged, such as ray-casting, to predict light interaction within a plant canopy [64]. We hope that our dataset will assist in the development of tools better suited to using these advanced representations in 3D phenotyping applications.

Currently, 3DGS relies on an initial point cloud for effective population of Gaussians in the scene, meaning that SfM remains a common prerequisite for most 3DGS models. 3DGS and NeRF also currently require extremely accurate initial camera poses to produce effective results. These camera poses are typically estimated through SfM, and as we noted earlier, SfM may fail to provide sufficiently accurate pose estimations, leading to errors in the reconstruction. This limitation is shared by other methods, including MVS, which also depend on accurate pose estimation for effective reconstruction. Nevertheless, it is important to note that using these methods repeatably may require an accurate system for camera capture.

### Automated dataset capture

A notable challenge of both approaches is their requirement for highly accurate camera positions. As shown in Table 2, slight errors in parameters can lead to lower-quality reconstructions. These can be obtained using a pipeline such as ours, combined with modern bundle adjustment algorithms, but we foresee these pipelines becoming a requirement for successful phenotyping using these state-of-the-art approaches. Our robotic image capture system and framework offer several advantages over static or limited capture setups. First, our system captures high-quality images around plants of various different sizes. By utilising robot path planning, dynamic generation of positions allows for flexible image capture should requirements change. This framework is highly customisable, ensuring repeatability across a variety of bespoke ROS setups, with the versatility of each setup being the main restriction in potential view capturing. Unlike unconstrained image capture setups (e.g., using a handheld camera), our system is calibrated such that even after refinement using bundle adjustment, all camera positions and reconstructions are represented in metric units. This is a feature not commonly found in other view synthesis datasets, and the use of ROS-compatible hardware allows other researchers to utilise this setup.

**Figure 7:**
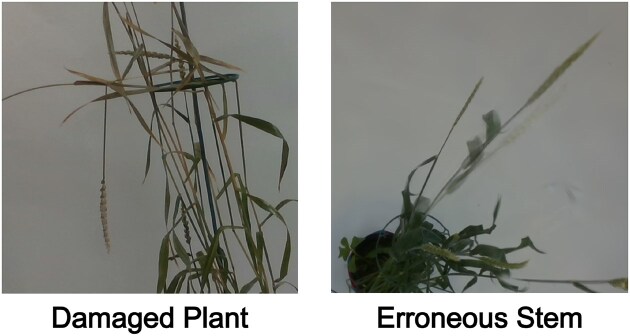
Examples of some of the issues with our dataset. Left shows an image of a damaged stem on plant bc1_1053_1 (16-04-24). Right image shows an image of an erroneous stem being rendered using 3DGS for plant bc1_1033_1 (06-03-24). This is due to a collision that stem had with the robot 2 stand, which caused it to move incorrectly during capturing.

Capturing high-quality data on living organisms such as plants remains a challenge. Transporting each plant from the greenhouse to the imaging setup occasionally resulted in damage, particularly to the spikes. With the larger wheat plants, stems occasionally became entangled with the stand of the second robot, causing discrepancies between views, resulting in floating artefacts in the reconstructions. These issues are shown in Fig. 7. We anticipate that the most effective solutions will be based within the growth environments themselves, and adapting our system to *in situ* robotics is an area of potential future research.

While the turntable was a critical component in allowing full range-of-view capturing in our setup, it did present several challenges. We suspect that rotating the plant sometimes caused micro-movements, impacting the final quality of the reconstructions. Furthermore, the consistent background meant that unwanted artefacts were generated during reconstruction. We implemented image segmentation prior to reconstruction to solve this problem, but we found that this did not consistently improve results and often led to poor reconstructions due to inaccuracies and inconsistencies between masks at different views. When rendering images outside of the capture radius, we noted that areas of background might be rendered instead of the plant. This caused particular issues when rendering the 3DGS images, as large Gaussians representing the white background may obscure the plant in some views. We recommend that utilising a turntable should only be used if full range of motion is not available for a particular setup. We found that postprocesses to remove these Gaussians were more effective than adapting the image capture or 3DGS training process. However, our method occasionally resulted in groups of residual background Gaussians; this approach would benefit from implementation of more robust background removal algorithms.

Many modern phenotyping pipelines, particularly those that make use of genomic techniques, require hundreds or even thousands of plant samples. At the time of this publication, we think that these remain out of reach of this technology, but a number of promising avenues could be explored by the community to achieve this goal. These reconstruction techniques require many images and accurately calibrated camera and scene parameters. Automated greenhouses that utilise robotics, conveyor belts, or a comparable delivery system [65] to autonomously transport plants to the capture setup offer a potential solution to this. A critical challenge with this approach lies in ensuring precise alignment and placement of the plants onto the turntable to maintain consistency between imaged plants. It is also imperative to implement measures that prevent potential collisions between the robotic arms and the plants being captured, given the potential variability in growth stages and morphological characteristics of the subject plants. To address these complexities, incorporating depth data from the cameras into the processing pipeline is a potential solution. This would allow for real-time determination of each plant’s spatial location and dimensions, ensuring accurate and efficient capture. For the time being, this technology is confined to controlled environments. In the future, improvements in selective scene reconstruction, through the inclusion of other machine learning processes such as segmentation, may allow in-field imaging to become possible.

## Conclusion

We have presented a new dataset for multiview reconstruction of plant shoots. By utilising a dual-robot image capture system and a turntable, we capture full 360-degree views of each plant, adapted to their size. This capture setup produces accurate camera positions in metric units, with associated high-resolution images, and depth information. Using this dataset, we demonstrate the strong performance of 2 recent approaches to view synthesis: neural radiance fields and 3D Gaussian splatting. We demonstrate state-of-the-art performance in both view synthesis and 3D model reconstruction. On our test data captured using a handheld scanner, the trained 3DGS and NeRF models had an average surface accuracy of 0.74 mm and 1.43 mm, respectively, compared to 2.32 mm and 7.23 mm for popular MVS and SfM techniques. We argue that both approaches will lead to a step-change in our ability to capture 3D models of plants, which have historically proved very challenging due to their complex shape, frequent occlusion, and self-similarity. We release all configuration files and scripts associated with our image capture system, which can be deployed on any ROS-compatible hardware. We also release our dataset of 112 wheat plants captured approximately $\sim$300 times each and associated camera position in metric units. Finally, we release all training scripts and trained NeRF and 3DGS models, as well as 3D reconstruction output across all plants. We hope that our study will provide opportunities for researchers exploring new and improved 3D phenotyping algorithms, 3D reconstruction and view synthesis research, and active vision systems.

## Availability of Source Code and Requirements

Project name: 3D Plant View Synthesis:

- Project homepage: https://github.com/Lewis-Stuart-11/3D-Plant-View-Synthesis [13]
- Operating system(s): Windows, Ubuntu
- Programming language: Python (>=3.8)
- License: Apache 2.0
- Any restrictions to use by nonacademics: None

Our code has also been archived in Software Heritage [66]. Functionality, such as Robotic View Capturing, 3DGS to Point Cloud, and our UR5 Configs files, are stored on separate GitHub repositories that can be accessed via the project README.

## Supplementary Material

giaf022_Supplemental_File

giaf022_GIGA-D-24-00315_Original_Submission

giaf022_GIGA-D-24-00315_Revision_1

giaf022_GIGA-D-24-00315_Revision_2

giaf022_GIGA-D-24-00315_Revision_3

giaf022_Response_to_Reviewer_Comments_Original_Submission

giaf022_Response_to_Reviewer_Comments_Revision_1

giaf022_Response_to_Reviewer_Comments_Revision_2

giaf022_Reviewer_1_Report_Original_Submission Deli Zhu -- 9/7/2024

giaf022_Reviewer_1_Report_Revision_1 Deli Zhu -- 1/21/2025

giaf022_Reviewer_2_Report_Original_Submission Rick van de Zedde -- 10/2/2024

giaf022_Reviewer_2_Report_Revision_1 Rick van de Zedde -- 1/11/2025

## Data Availability

Our main dataset and data further supporting this work are openly available at https://plantimages.nottingham.ac.uk [11] and in the *GigaScience* repository, GigaDB [12]. Our dataset annotation is available at the DOME Registry [67].

## Additional Files

**Supplementary Fig. S1**. ROS visualisation of the dual robot setup that needs optimising. Important transforms, and their links, are displayed.

**Supplementary Fig. S2**. ROS visualisation of the TCP transform and the predicted camera lens transform. The TCP transform is positioned at the end of the robot, while the camera lens transform is positioned left of the centre of the camera.

**Supplementary Fig. S3**. ROS visualisation of the transform tree that maps the camera lens of the first robot to the centre of the turntable. The goal is to accurately determine the mapping between the robot 1 pedestal and the centre of the turntable.

**Supplementary Fig. S4**. ROS visualisation of the mapping of both robot pedestals to the centre of the turntable. Transform from robot 1 pedestal to the turntable has been optimised. Transform from robot 2 pedestal to the turntable currently still needs to be optimised.

## Abbreviations

3DGS: 3D Gaussian splatting; ATE: absolute trajectory error; CNN: convolutional neural network; FPS: frames per second; LiDAR: Light detection and ranging; LPIPS: learned perceptual image patch similarity; MVS: multiview stereo; NeRF: neural radiance field; PSNR: peak signal-to-noise ratio; RGB: red, green, and blue; ROS: robot operating system; SD: standard deviation; SfM: structure from motion; SSIM: structural similarity index measure; TCP: tool center point; UoN: University of Nottingham; URDF: Unified Robot Description Format.
