# Supplementary material for: High-fidelity wheat plant reconstruction using 3D Gaussian splatting and neural radiance fields
Source: Gigascience. 2025 Mar 26;14:giaf022. doi: 10.1093/gigascience/giaf022 (PMC11945317; doi:10.1093/gigascience/giaf022)
Supplement: giaf022_Supplemental_File [file giaf022_supplemental_file.pdf]

Supplementary Material for *High-fidelity Wheat Plant Reconstruction using 3D Gaussian Splatting and Neural Radiance Fields*

Lewis A G Stuart[1], Darren M Wells[2], Jonathan A Atkinson[2], Simon Castle-Green[1], Jack Walker[2], and Michael P Pound[1]

[1]School of Computer Science, University of Nottingham, UK

[2]School of Biosciences, University of Nottingham, UK

1 Calibration

Current 3D reconstruction approaches often require pre-determined camera poses to facilitate effective reconstruction. SfM is typically used to determine each camera pose, but we chose to initialise our process with the recorded kinematic transforms from our robot setup. In order for these transforms to be accurate, we calibrated our system using pipeline that we outline below. In brief, the process requires the following steps:

- Mapping of end of each robot arm to the optical centre of each camera.
- Calibration of the turntable to the base of a single robot.
- Calibration of the dual robot system.

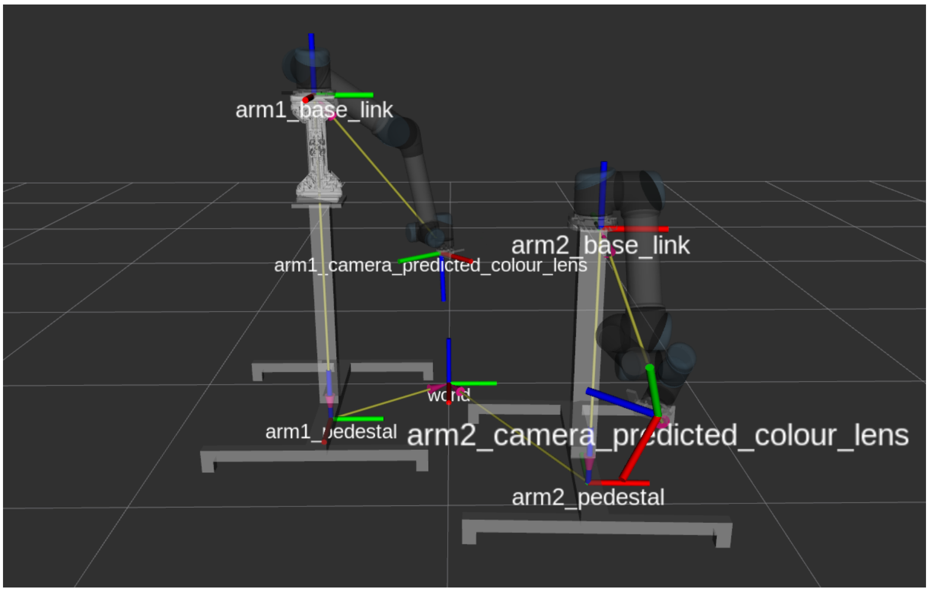

Figure 1: ROS visualisation of the dual robot setup that needs optimising. Important transforms, and their links, are displayed.

1.1 RealSense D345

We map the tool centre point (TCP) of the robot, which is where the camera is mounted, to the optical centre of the RealSense camera. The provided UR5 MoveIt URDF file accurately determines the position of the tool center point of each UR5. We combined the dimensions of our custom 3D printed brackets with the dimensions of the Realsense cameras using the official camera specification documentation. This also provided an estimated offset from the centre of the camera to the RGB camera lens, allowing us to calculate a mapping from the UR5 TCP to the camera lens.

We still found that the predicted position and orientation of the camera lens required further refinement for the highest accuracy. We used the MoveIt Hand-Eye Camera Calibration package to produce a final transform from the robot TCP to the camera optical centre. We printed a calibration target and captured 15 images using the robot around the target. This package then optimised the difference between a predicted camera lens position and the calculated camera lens position based on the calibration target in the frame. Finally, we fine-tuned this transform, through a visual estimate of the camera optical centre. We performed this operation for both robots independently. The mapping from robot base to camera optical centre can now be calculated for any robot pose using a combination of the camera mapping, and the internal robot joint positions.

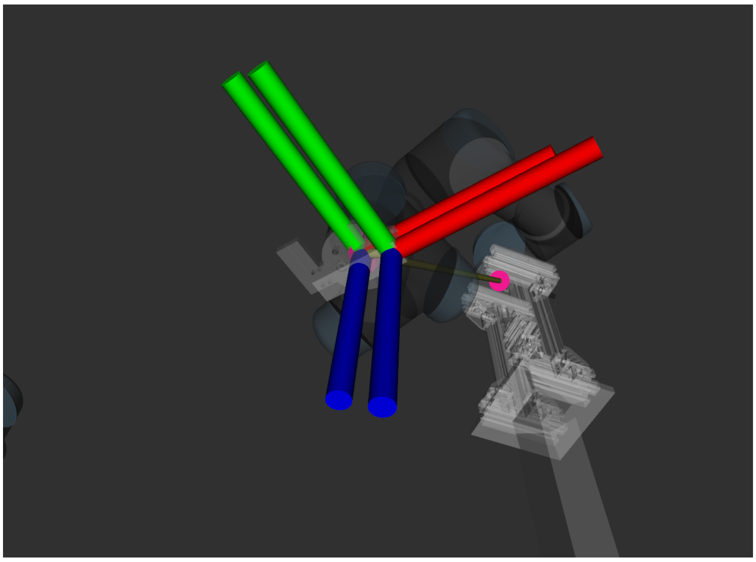

Figure 2: ROS visualisation of the TCP transform and the predicted camera lens transform. The TCP transform is positioned at the end of the robot, while the camera lens transform is positioned left of the centre of the camera.

1.2 Turntable

We next calibrated the turntable position with respect to a single robot. We placed a standard checkerboard in the centre of the turntable such that the centre of the checkerboard approximately aligned with the centre of the turntable. We then captured 50 images from varied poses around the turntable using robot 1, recording the camera positions of each of the captured images. We performed sparse reconstruction of the scene using COLMAP, with parameters initialised from our previously calculated intrinsic and extrinsic camera parameters. The output of this step is a series of sparse features, matched and projected into 3D space within the world coordinate system. This allowed us to identify key positions of the turntable in 3D using the COLMAP GUI, which gave us an estimate of the turntable centre by taking the mean of four features chosen from the four inner checkerboard squares.
We then took a series of images of the turntable on a fixed X-axis, simulating the images that the robot will take during view capture. We fine-tuned the turntable position by manually adjusting value until the turntable centre is in the centre of each image.
For convenience, we translated all calculated coordinates such that the centre of the turntable now represents the origin of the world coordinate system.

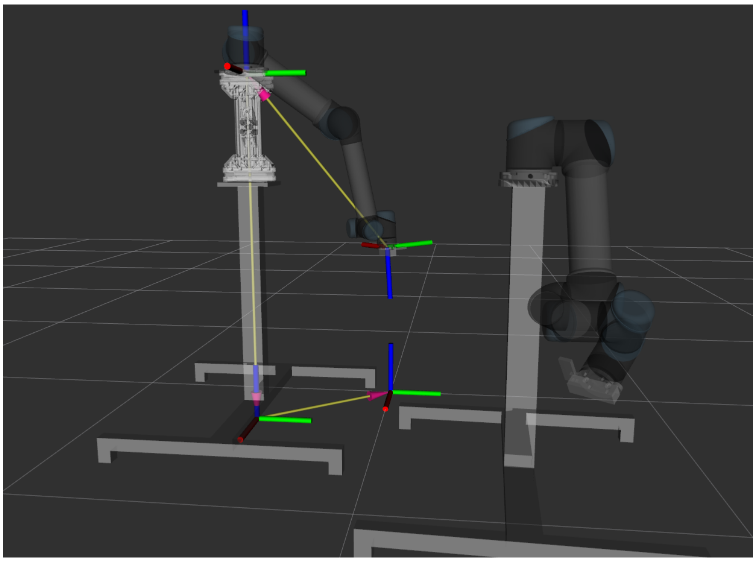

Figure 3: ROS visualisation of the transform tree that maps the camera lens of the first robot to the centre of the turntable. The goal is to accurately determine the mapping between the robot 1 pedestal and the centre of the turntable.

1.3 Dual Robots

To calibrate robot 2, we first produce an approximate by using real-world measurement from the base of both robots, and then optimising this measurement to become more accurate. Our approach involves capturing a set of views reachable from both cameras, in the same predicted camera positions, and optimising the second robot position to align these views. We calculated a set of 50 camera poses of the scene that can be reached by both robots, and captured a set of images for each of these camera poses for both robots. Our goal is to find a global transform for robot 2 that maps each image from robot 2 to robot 1 in 3D. We create a sparse point cloud of the scene with images and camera poses from robot 1, using the same method as described in 1.2. We then determine the correct camera poses of robot 2 by registering the captured images from robot 2 based on the current generated point cloud. We used a differential evolution optimisation algorithm to learn a global transformation mapping the approximate locations of robot 2 images to the true calibrated locations. Now, we have a global transformation matrix that can map any camera pose captured from robot 2 into the correct coordinate system. Therefore, any transform generated by robot 2 during image capture was multiplied by this matrix to calculate its true camera pose. We have included a script in our Dual Robot Github that contains functions that can generate this global transform in a straightforward manner.

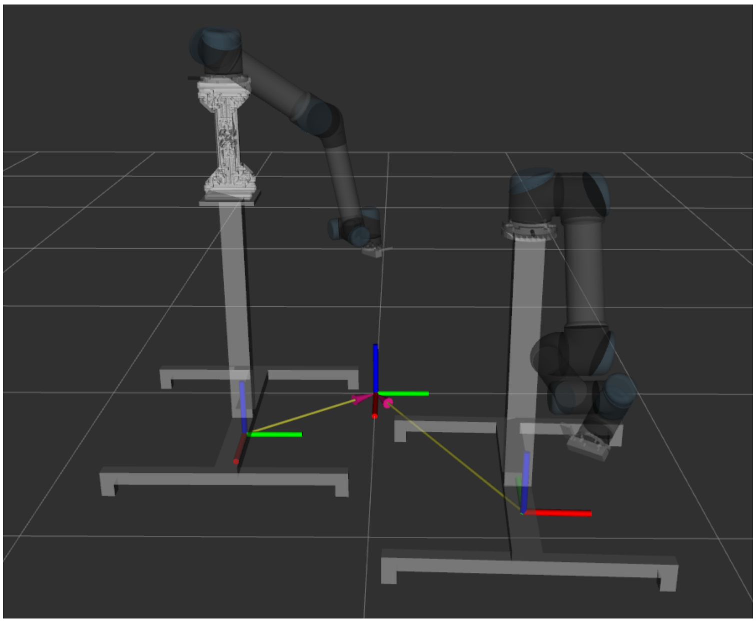

Figure 4: ROS visualisation of the mapping of both robot pedestals to the centre of the turntable. Transform from robot 1 pedestal to the turntable has been optimised. Transform from robot 2 pedestal to the turntable currently still needs to be optimised.

2 Rendered Images

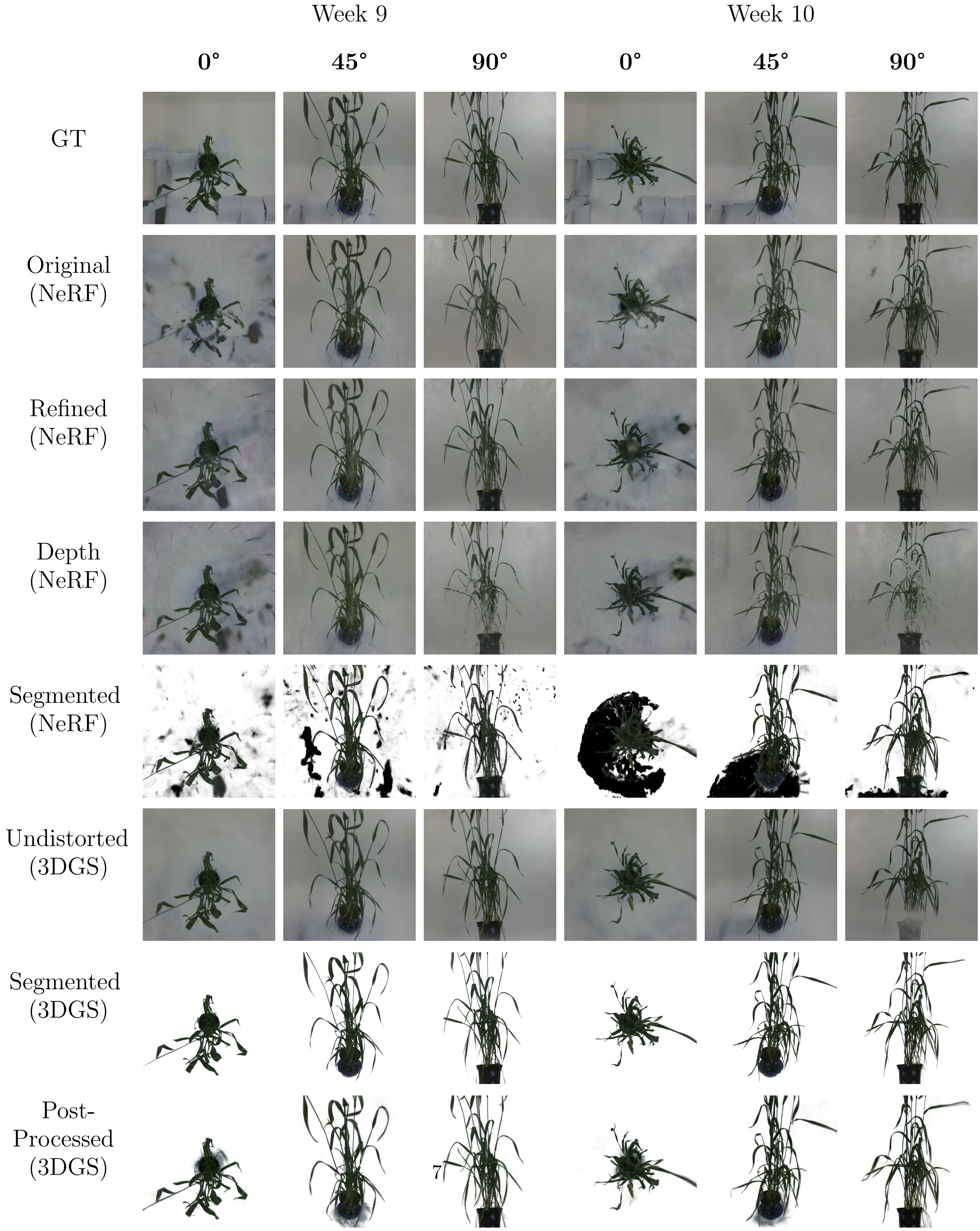

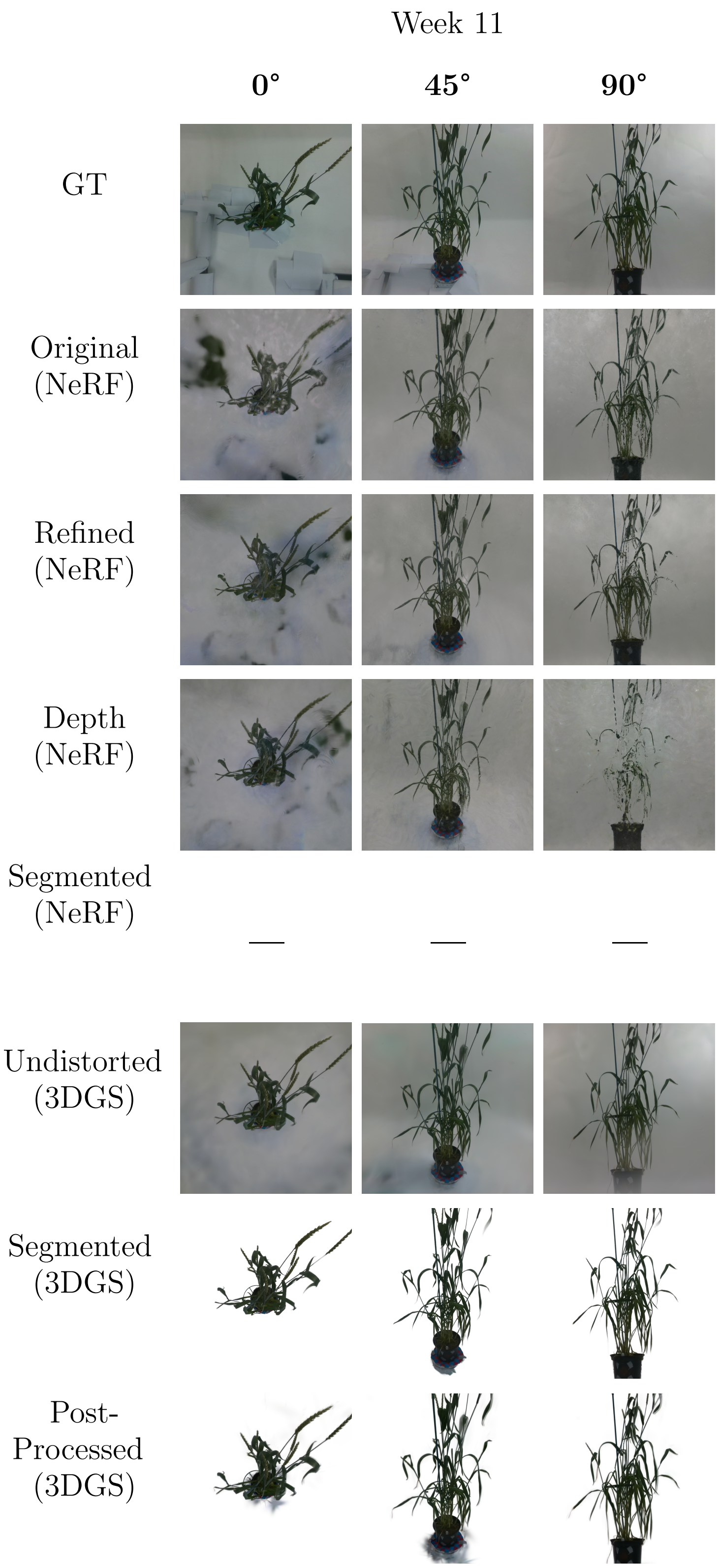
Week 11
0°
45°
90°
GT
Original (NeRF)
Refined (NeRF)
Depth (NeRF)
Segmented (NeRF)
—
—
—
Undistorted (3DGS)
Segmented (3DGS)
Post-Processed (3DGS)

3 Full Results

3.1 NeRF Original Transforms

**Plant Name**	Date	PSNR	SSIM	LPIPS	PSNR MASK
bc1_1033_3	06/03/24	20.958	0.783	0.299	14.203
	13/02/24	21.936	0.846	0.211	17.618
	13/03/24	21.096	0.793	0.272	14.151
	16/04/24	21.896	0.81	0.251	15.558
	20/03/24	20.33	0.784	0.297	14.232
bc1_1050	06/03/24	20.731	0.755	0.311	14.258
	13/02/24	22.638	0.843	0.192	16.449
	13/03/24	20.922	0.779	0.28	14.058
	16/04/24	21.753	0.805	0.27	14.951
	20/03/24	20.988	0.8	0.263	14.745
bc1_1051	06/03/24	19.723	0.757	0.298	14.106
	13/03/24	20.146	0.788	0.266	14.399
	14/02/24	22.87	0.843	0.202	17.16
	16/04/24	19.401	0.752	0.304	13.798
	20/03/24	19.415	0.773	0.315	13.713
bc1_1052_2	06/03/24	21.008	0.786	0.276	14.697
	13/03/24	21.023	0.794	0.266	14.659
	14/02/24	23.401	0.868	0.196	17.71
	16/04/24	21.226	0.777	0.264	15.408
	20/03/24	20.254	0.785	0.291	14.607
bc1_1053_1	06/03/24	20.741	0.78	0.291	14.497
	13/03/24	20.753	0.794	0.262	14.662
	14/02/24	22.206	0.814	0.219	16.649
	16/04/24	21.998	0.816	0.25	15.558
	20/02/24	20.375	0.75	0.25	15.937
	20/03/24	20.952	0.792	0.264	14.447

bc1_1053_2	07/03/24	19.55	0.731	0.334	14.081
	14/02/24	23.216	0.85	0.199	17.376
	14/03/24	21.09	0.794	0.282	15.024
	18/04/24	21.093	0.784	0.282	15.37
	21/03/24	20.386	0.772	0.284	14.235
bc1_1054	06/03/24	20.165	0.756	0.299	14.454
	13/03/24	20.492	0.787	0.283	14.307
	14/02/24	22.973	0.844	0.211	17.511
	16/04/24	22.209	0.833	0.236	16.082
	20/03/24	20.572	0.797	0.288	14.645
cs_1	05/03/24	20.682	0.806	0.268	14.419
	12/03/24	21.805	0.84	0.24	14.654
	13/02/24	23.702	0.889	0.182	17.679
	15/04/24	21.905	0.833	0.239	15.224
	19/02/24	23.702	0.873	0.19	17.292
	19/03/24	20.378	0.812	0.302	14.362
cs_2	05/03/24	20.804	0.783	0.277	14.713
	12/03/24	21.053	0.816	0.256	14.425
	13/02/24	23.22	0.86	0.194	17.1
	15/04/24	20.959	0.813	0.253	15.033
	19/02/24	22.784	0.836	0.22	16.51
	19/03/24	19.525	0.782	0.314	13.935
cs_2b_2eb_4_1	07/03/24	20.583	0.782	0.326	13.701
	14/02/24	24.348	0.886	0.175	17.433
	14/03/24	20.953	0.794	0.282	14.695
	18/04/24	20.402	0.762	0.305	14.544
	20/02/24	23.836	0.877	0.2	17.313
	21/03/24	20.093	0.758	0.294	14.302

cs_2d_2eb_1	07/03/24	21.569	0.798	0.28	13.995
	14/02/24	22.827	0.832	0.201	17.119
	14/03/24	21.68	0.8	0.265	14.518
	18/04/24	23.219	0.848	0.258	15.98
	20/02/24	20.95	0.786	0.23	15.894
	21/03/24	21.508	0.792	0.282	14.918
cs_2d_2eb_2	07/03/24	20.917	0.785	0.287	14.316
	14/02/24	22.676	0.837	0.195	16.805
	14/03/24	22.013	0.823	0.247	14.517
	18/04/24	22.697	0.835	0.235	16.273
	21/03/24	20.815	0.793	0.305	14.173
cs_2d_2eb_4_1	06/03/24	20.77	0.796	0.284	14.176
	13/03/24	20.476	0.782	0.306	13.857
	14/02/24	22.448	0.839	0.206	17.383
	16/04/24	22.584	0.845	0.254	15.285
	20/03/24	21.408	0.818	0.258	14.681
gru_da5j_1	05/03/24	20.034	0.779	0.255	14.665
	12/03/24	20.859	0.829	0.236	15.152
	13/02/24				
	15/04/24	21.89	0.849	0.225	15.791
	19/02/24	22.601	0.846	0.21	16.43
	19/03/24	20.336	0.823	0.282	14.65
gru_da5j_2	05/03/24	20.726	0.787	0.254	14.638
	12/02/24	23.094	0.857	0.201	16.981
	12/03/24	21.206	0.812	0.242	14.977
	15/04/24	22.067	0.835	0.243	15.978
	19/02/24	22.446	0.818	0.303	16.718
	19/03/24	20.348	0.808	0.35	14.828
gru_da5j_3	05/03/24	20.586	0.813	0.332	14.871
	12/02/24	23.313	0.885	0.268	17.33
	12/03/24	21.761	0.85	0.32	15.415
	15/04/24	21.541	0.826	0.329	15.622
	19/02/24	23.461	0.875	0.261	16.795
	19/03/24	21.061	0.834	0.335	15.293

langdon_1	07/03/24	20.985	0.773	0.365	14.886
	14/02/24	21.718	0.797	0.32	16.245
	14/03/24	20.448	0.736	0.416	14.944
	18/04/24	20.163	0.724	0.471	14.412
	21/03/24	19.413	0.707	0.441	14.797
langdon_2	05/03/24	20.456	0.752	0.376	15.205
	12/02/24	20.947	0.795	0.342	15.977
	12/03/24	21.304	0.789	0.358	15.883
	15/04/24	19.951	0.748	0.404	14.34
	19/03/24	20.229	0.784	0.381	15.176
	20/02/24	20.489	0.768	0.349	15.794
langdon_3	07/03/24	20.398	0.772	0.391	14.117
	14/02/24	22.703	0.836	0.301	16.568
	14/03/24	20.748	0.779	0.382	15.343
	18/04/24	20.72	0.772	0.375	15.092
	21/03/24	19.978	0.767	0.387	15.361
langdon_4	05/03/24	19.448	0.741	0.395	14.402
	12/03/24	20.438	0.77	0.389	15.194
	13/02/24	22.103	0.818	0.311	16.021
	15/04/24	19.762	0.746	0.407	14.356
	19/03/24	19.343	0.753	0.414	14.087
	20/02/24	21.062	0.787	0.332	15.951

3.2 NeRF Refined Transforms

**Plant Name**	Date	PSNR	SSIM	LPIPS	PSNR MASK
	06/03/24	24.268	0.864	0.192	18.843
	13/02/24	23.191	0.867	0.18	19.876
bc1_1033_3	13/03/24	23.574	0.846	0.197	18.109
	16/04/24	24.813	0.892	0.181	20.975
	20/03/24	23.683	0.882	0.198	20.117
	06/03/24	23.857	0.839	0.197	18.355
	13/02/24	23.677	0.865	0.171	18.114
bc1_1050	13/03/24	24.256	0.865	0.183	19.47
	16/04/24	24.983	0.885	0.193	20.364
	20/03/24	23.376	0.866	0.212	18.651
	06/03/24	24.423	0.855	0.206	20.718
	13/03/24	23.531	0.849	0.205	19.756
bc1_1051	14/02/24	24.271	0.864	0.178	19.154
	16/04/24	23.711	0.839	0.2	19.703
	20/03/24	22.817	0.856	0.209	19.79
	06/03/24	24.017	0.851	0.2	18.878
	13/03/24	24.4	0.868	0.193	20.161
bc1_1052_2	14/02/24	24.783	0.889	0.156	19.98
	16/04/24	23.929	0.858	0.202	19.441
	20/03/24	23.218	0.851	0.231	19.284
	24.687	0.868	0.19	19.765	
	13/03/24	24.203	0.868	0.204	19.62
	14/02/24	23.882	0.848	0.183	19.089
bc1_1053_1	16/04/24	24.842	0.881	0.185	19.935
	20/02/24	24.043	0.829	0.172	19.901
	20/03/24	23.75	0.868	0.18	20.015

	07/03/24	23.44	0.82	0.245	19.835
	14/02/24	25.018	0.875	0.161	19.7
bc1_1053_2	14/03/24	24.124	0.867	0.2	19.725
	18/04/24	24.004	0.869	0.22	18.814
	21/03/24	23.681	0.871	0.189	20.408
	06/03/24	23.437	0.826	0.214	18.843
	13/03/24	23.887	0.854	0.203	18.591
bc1_1054	14/02/24	24.713	0.874	0.169	19.816
	16/04/24	23.868	0.871	0.191	18.365
	20/03/24	23.396	0.868	0.215	19.933
	05/03/24	24.896	0.891	0.171	22.247
	12/03/24	25.391	0.907	0.174	21.701
cs_1	13/02/24	25.23	0.918	0.161	21.982
	15/04/24	25.955	0.91	0.176	21.886
	19/02/24	25.607	0.902	0.155	21.024
	19/03/24	22.514	0.868	0.239	18.251
	05/03/24	24.729	0.863	0.188	20.106
	12/03/24	25.035	0.89	0.177	19.978
cs_2	13/02/24	24.775	0.881	0.153	19.405
	15/04/24	25.526	0.906	0.178	22.019
	19/02/24	24.468	0.862	0.185	18.793
	19/03/24	22.285	0.87	0.215	18.739
	07/03/24	24.437	0.862	0.215	19.46
	14/02/24	25.966	0.902	0.158	21.134
cs_2b_2eb_4_1	14/03/24	24.379	0.867	0.196	19.317
	18/04/24	23.21	0.846	0.221	18.91
	20/02/24	25.687	0.898	0.155	20.337
	21/03/24	23.094	0.844	0.206	18.941
	07/03/24	24.992	0.878	0.182	19.066
	14/02/24	24.367	0.866	0.18	18.826
cs_2d_2eb_1	14/03/24	24.646	0.875	0.178	19.793
	18/04/24	25.168	0.897	0.192	20.733
	20/02/24	24.706	0.862	0.171	20.847
	21/03/24	24.574	0.881	0.182	20.642

cs_2d_2eb_2	07/03/24	24.085	0.849	0.199	18.904
	14/02/24	24.552	0.865	0.177	19.695
	14/03/24	25.117	0.894	0.175	20.182
	18/04/24	25.593	0.908	0.173	22.191
	21/03/24	23.931	0.875	0.206	19.807
cs_2d_2eb_4_1	06/03/24	25.454	0.892	0.169	20.789
	13/03/24	24.619	0.885	0.184	20.572
	14/02/24	24.372	0.866	0.168	19.557
	16/04/24	25.366	0.905	0.188	20.823
	20/03/24	24.208	0.893	0.181	20.036
gru_da5j_1	05/03/24	23.742	0.864	0.187	19.858
	12/03/24	24.574	0.913	0.156	22.037
	13/02/24				
	15/04/24	24.052	0.893	0.175	20.356
	19/02/24	24.88	0.884	0.177	20.339
	19/03/24	22.951	0.893	0.203	19.148
gru_da5j_2	05/03/24	23.992	0.86	0.167	19.995
	12/02/24	25.522	0.905	0.149	22.202
	12/03/24	24.636	0.881	0.165	19.964
	15/04/24	25.265	0.898	0.172	20.442
	19/02/24	23.73	0.84	0.268	18.541
	19/03/24	21.736	0.848	0.308	17.16

gru_da5j_3	05/03/24				
	12/02/24	24.34	0.901	0.244	19.944
	12/03/24	23.915	0.888	0.275	19.419
	15/04/24	23.724	0.864	0.276	18.705
	19/02/24	25.185	0.897	0.232	20.339
	19/03/24	22.634	0.875	0.286	18.072
langdon_1	07/03/24	23.484	0.829	0.296	18.491
	14/02/24	22.929	0.818	0.289	17.901
	14/03/24	22.74	0.793	0.358	18.313
	18/04/24				
	21/03/24	21.038	0.766	0.407	17.151
langdon_2	05/03/24	22.643	0.792	0.322	18.249
	12/02/24	22.558	0.827	0.305	18.253
	12/03/24	23.059	0.829	0.304	18.521
	15/04/24	22.504	0.819	0.345	18.22
	19/03/24	21.584	0.831	0.341	17.127
	20/02/24	21.976	0.795	0.317	18.099
langdon_3	07/03/24	22.648	0.822	0.319	18.145
	14/02/24	23.757	0.855	0.282	18.094
	14/03/24	23.079	0.831	0.336	18.443
	18/04/24	22.833	0.823	0.335	17.916
	21/03/24	21.989	0.8	0.359	17.98
langdon_4	05/03/24	21.336	0.784	0.334	17.123
	12/03/24	22.819	0.82	0.337	18.583
	13/02/24	23.672	0.84	0.284	18.182
	15/04/24	22.242	0.797	0.355	17.815
	19/03/24	20.87	0.792	0.367	16.405
	20/02/24	22.89	0.821	0.301	18.475

3.3 NeRF Segmented Transforms

**Plant Name**	Date	PSNR MASK
bc1_1033_3	06/03/24	3.71
	13/02/24	5.429
	13/03/24	5.603
	16/04/24	3.812
	20/03/24	4.192
bc1_1050	06/03/24	14.111
	13/02/24	1.531
	13/03/24	1.811
	16/04/24	1.877
	20/03/24	15.316
bc1_1051	06/03/24	16.395
	13/03/24	16.972
	14/02/24	1.512
	16/04/24	15.811
	20/03/24	1.648
bc1_1052_2	06/03/24	15.56
	13/03/24	15.169
	14/02/24	13.736
	16/04/24	2.134
	20/03/24	1.783
bc1_1053_1	06/03/24	1.858
	13/03/24	1.797
	14/02/24	15.027
	16/04/24	2.034
	20/02/24	15.737
	20/03/24	1.806

**Plant Name**	Date	PSNR MASK
bc1_1053_2	07/03/24	1.865
	14/02/24	1.597
	14/03/24	17.544
	18/04/24	1.903
	21/03/24	1.817
bc1_1054	06/03/24	14.126
	13/03/24	1.691
	14/02/24	16.549
	16/04/24	1.937
	20/03/24	3.964
cs_1	05/03/24	1.702
	12/03/24	1.719
	13/02/24	2.231
	15/04/24	16.227
	19/02/24	1.493
	19/03/24	11.327
cs_2	05/03/24	14.905
	12/03/24	1.78
	13/02/24	1.626
	15/04/24	1.707
	19/02/24	17.497
	19/03/24	13.025
cs_2b_2eb_4_1	07/03/24	1.735
	14/02/24	1.552
	14/03/24	14.833
	18/04/24	16.09
	20/02/24	1.48
	21/03/24	1.784

**Plant Name**	Date	PSNR MASK
cs_2d_2eb_1	07/03/24	1.863
	14/02/24	1.635
	14/03/24	1.831
	18/04/24	2.243
	20/02/24	15.977
	21/03/24	1.911
cs_2d_2eb_2	07/03/24	14.352
	14/02/24	1.702
	14/03/24	1.807
	18/04/24	16.697
	21/03/24	1.783
cs_2d_2eb_4_1	06/03/24	1.807
	13/03/24	1.816
	14/02/24	1.634
	16/04/24	2.062
	20/03/24	15.171
gru_da5j_1	05/03/24	1.575
	12/03/24	14.705
	13/02/24	14.176
	15/04/24	18.793
	19/02/24	1.505
	19/03/24	1.786
gru_da5j_2	05/03/24	16.441
	12/02/24	1.6
	12/03/24	17.191
	15/04/24	1.635
	19/02/24	14.441
	19/03/24	13.298

**Plant Name**	Date	PSNR MASK
gru_da5j_3	05/03/24	3.095
	12/02/24	5.789
	12/03/24	4.98
	15/04/24	4.769
	19/02/24	5.396
	19/03/24	5.649
langdon_1	07/03/24	3.577
	14/02/24	4.697
	14/03/24	3.594
	18/04/24	
	21/03/24	3.607
langdon_2	05/03/24	4.664
	12/02/24	4.529
	13/03/24	4.475
	15/04/24	4.112
	19/03/24	4.97
	20/02/24	4.247
langdon_3	07/03/24	4.006
	14/02/24	5.07
	14/03/24	4.178
	18/04/24	4.402
	21/03/24	4.338
langdon_4	05/03/24	4.34
	12/02/24	4.377
	13/03/24	4.58
	15/04/24	4.276
	19/03/24	4.451
	20/02/24	4.416

3.4 NeRF Depth Transforms

**Plant Name**	Date	PSNR	SSIM	LPIPS	PSNR MASK
bc1_1033_3	06/03/24	23.826	0.859	0.234	17.79
	13/02/24	22.414	0.874	0.197	17.172
	13/03/24	23.316	0.854	0.257	17.179
	16/04/24	24.161	0.872	0.217	18.039
	20/03/24	23.491	0.868	0.235	18.058
bc1_1050	06/03/24	23.389	0.831	0.251	17.363
	13/02/24	24.329	0.872	0.187	18.973
	13/03/24	24.284	0.862	0.218	18.282
	16/04/24	24.318	0.87	0.234	18.073
	20/03/24	23.127	0.852	0.246	16.541
bc1_1051	06/03/24	23.575	0.839	0.249	19.573
	13/03/24	24.146	0.858	0.246	19.259
	14/02/24	25.729	0.883	0.172	20.01
	16/04/24	23.793	0.85	0.234	19.125
	20/03/24	22.713	0.844	0.273	18.096
bc1_1052_2	06/03/24	24.158	0.849	0.242	18.439
	13/03/24	22.994	0.843	0.25	16.932
	14/02/24	23.569	0.903	0.185	17.359
	16/04/24	23.935	0.845	0.241	17.689
	20/03/24	22.408	0.839	0.277	16.803
bc1_1053_1	06/03/24	24.241	0.858	0.231	18.873
	13/03/24	24.123	0.863	0.234	18.905
	14/02/24	25.058	0.869	0.181	19.58
	16/04/24	24.625	0.875	0.237	18.717
	20/02/24	24.482	0.842	0.196	19.883
	20/03/24	23.466	0.859	0.25	17.727

bc1_1053_2	07/03/24	23.318	0.832	0.249	18.754
	14/02/24	25.863	0.881	0.169	20.088
	14/03/24	23.8	0.866	0.235	18.25
	18/04/24	23.91	0.865	0.241	18.66
	21/03/24	23.589	0.848	0.243	18.425
bc1_1054	06/03/24	23.546	0.822	0.245	18.247
	13/03/24	23.381	0.85	0.249	17.64
	14/02/24	25.931	0.887	0.168	20.492
	16/04/24	24.397	0.88	0.232	18.734
	20/03/24	23.611	0.868	0.244	18.206
cs_1	05/03/24	24.848	0.879	0.201	19.482
	12/03/24	24.745	0.889	0.222	18.904
	13/02/24	24.108	0.917	0.178	18.42
	15/04/24	25.243	0.889	0.214	19.61
	19/02/24	26.525	0.906	0.154	21.171
	19/03/24	22.531	0.865	0.256	16.392
cs_2	05/03/24	24.667	0.857	0.216	18.477
	12/03/24	24.171	0.881	0.223	17.834
	13/02/24	26.673	0.906	0.15	20.868
	15/04/24	24.753	0.888	0.219	18.962
	19/02/24	25.957	0.885	0.185	20.17
	19/03/24	22.12	0.856	0.271	16.545
cs_2b_2eb_4_1	07/03/24	24.154	0.853	0.236	18.002
	14/02/24	26.105	0.899	0.158	19.094
	14/03/24	23.753	0.855	0.252	17.845
	18/04/24	23.146	0.846	0.251	17.615
	20/02/24	26.983	0.91	0.158	21.102
	21/03/24	23.221	0.843	0.249	17.675

cs_2d_2eb_1	07/03/24	24.594	0.867	0.223	18.423
	14/02/24	25.591	0.872	0.17	19.78
	14/03/24	24.33	0.87	0.245	17.34
	18/04/24	25.31	0.887	0.222	18.538
	20/02/24	24.975	0.864	0.178	20.596
	21/03/24	24.114	0.866	0.235	17.272
cs_2d_2eb_2	07/03/24	23.874	0.852	0.251	17.807
	14/02/24	24.933	0.868	0.18	19.268
	14/03/24	24.483	0.876	0.243	17.641
	18/04/24	25.737	0.898	0.19	19.631
	21/03/24	23.879	0.864	0.243	17.869
cs_2d_2eb_4_1	06/03/24	24.878	0.877	0.213	18.552
	13/03/24	24.462	0.874	0.214	19.09
	14/02/24	23.469	0.877	0.191	17.775
	16/04/24	25.458	0.886	0.224	18.422
	20/03/24	23.562	0.877	0.246	17.315
gru_da5j_1	05/03/24	25.634	0.879	0.163	21.281
	12/03/24	24.53	0.895	0.193	19.44
	13/02/24				
	15/04/24	24.825	0.902	0.182	20.03
	19/02/24	26.346	0.898	0.156	21.127
	19/03/24	23.019	0.886	0.221	17.996
gru_da5j_2	05/03/24	25.174	0.875	0.191	19.488
	12/02/24	27.202	0.909	0.153	21.797
	12/03/24	25.462	0.894	0.18	19.706
	15/04/24	24.72	0.883	0.209	18.634
	19/02/24	23.776	0.883	0.184	17.461
	19/03/24	21.449	0.881	0.267	15.216

	05/03/24				
	12/02/24	24.02	0.92	0.18	18.231
gru_da5j_3	12/03/24	21.944	0.91	0.214	15.369
	15/04/24	22.143	0.894	0.22	15.199
	19/02/24	24.62	0.913	0.182	17.72
	19/03/24	21.793	0.897	0.247	15.173
	07/03/24	23.963	0.867	0.244	17.851
	14/02/24	23.372	0.875	0.199	17.022
langdon_1	14/03/24	22.269	0.846	0.267	15.784
	18/04/24				
	21/03/24	22.029	0.831	0.262	16.68
	05/03/24	22.589	0.854	0.225	16.414
	12/02/24	23.146	0.877	0.2	17.459
langdon_2	12/03/24	23.083	0.876	0.248	16.658
	15/04/24	23.3	0.865	0.236	17.662
	19/03/24	22.479	0.868	0.268	16.912
	20/02/24	23.206	0.864	0.188	17.554
	07/03/24	22.818	0.872	0.244	16.443
	14/02/24	23.749	0.888	0.198	17.256
langdon_3	14/03/24	22.917	0.869	0.248	16.786
	18/04/24	22.27	0.861	0.25	17.126
	21/03/24	22.259	0.859	0.248	16.712
	05/03/24	21.289	0.84	0.249	15.337
	12/03/24	22.216	0.867	0.263	15.82
langdon_4	13/02/24	23.942	0.882	0.2	16.959
	15/04/24	22.629	0.859	0.241	17.075
	19/03/24	21.355	0.847	0.287	16.702
	20/02/24	22.861	0.863	0.206	16.701

3.5 NeRF Colmap Transforms

**Plant Name**	Date	PSNR	SSIM	LPIPS	PSNR MASK
bc1_1033_3	20/03/24	22.974	0.866	0.228	19.789
bc1_1050	20/03/24	22.545	0.852	0.272	18.93
bc1_1051	20/03/24	21.538	0.835	0.275	18.57
bc1_1052_2	20/03/24	22.333	0.802	0.265	17.411
bc1_1053_1	20/03/24	23.907	0.872	0.207	20.689
bc1_1053_2	21/03/24	23.6	0.855	0.238	19.918
bc1_1054	20/03/24	23.395	0.867	0.24	20.556
cs_1	19/03/24	21.088	0.806	0.377	13.967
cs_2	19/03/24	20.374	0.764	0.423	13.758
cs_2b_2eb_4_1	21/03/24	22.847	0.843	0.25	18.536
cs_2d_2eb_1	21/03/24	24.31	0.887	0.221	20.742
cs_2d_2eb_2	21/03/24	23.527	0.863	0.236	18.24
cs_2d_2eb_4_1	20/03/24	23.728	0.892	0.227	20.211
gru_da5j_1	19/03/24	21.285	0.837	0.297	16.555
gru_da5j_2	19/03/24	21.23	0.808	0.35	14.624
gru_da5j_3	19/03/24	21.787	0.836	0.327	14.847
langdon_1	21/03/24	21.79	0.808	0.31	17.765
langdon_2	19/03/24	17.892	0.673	0.559	13.636
langdon_3	21/03/24	20.618	0.768	0.373	16.72
langdon_4	19/03/24	19.019	0.726	0.496	12.923

3.6 3DGS Undistorted Transforms

**Plant Name**	Date	PSNR	SSIM	LPIPS	PSNR MASK
bc1_1033_3	06/03/24	28.937	0.952	0.143	25.569
	13/02/24	28.27	0.945	0.135	25.439
	13/03/24	28.494	0.944	0.154	24.571
	16/04/24	28.465	0.955	0.146	27.305
	20/03/24	28.671	0.954	0.145	28.012
bc1_1050	06/03/24	27.657	0.936	0.155	25.726
	13/02/24	29.371	0.952	0.125	25.56
	13/03/24	28.601	0.947	0.156	25.665
	16/04/24	27.815	0.951	0.149	26.719
	20/03/24	25.353	0.938	0.164	25.724
bc1_1051	06/03/24	28.547	0.948	0.131	26.047
	13/03/24	26.577	0.931	0.175	26.236
	14/02/24	29.18	0.952	0.13	26.716
	16/04/24	28.448	0.949	0.135	27.055
	20/03/24	28.525	0.949	0.139	26.16
bc1_1052_2	06/03/24	27.684	0.942	0.141	26.379
	13/03/24	27.278	0.939	0.168	24.457
	14/02/24	28.296	0.955	0.137	26.534
	16/04/24	27.79	0.938	0.162	24.596
	20/03/24	27.586	0.945	0.156	26.765
bc1_1053_1	06/03/24	28.664	0.951	0.142	26.061
	13/03/24				
	14/02/24	28.161	0.948	0.123	27.04
	16/04/24	27.653	0.947	0.16	25.779
	20/02/24	27.158	0.936	0.138	27.463
	21/03/24	28.157	0.95	0.143	26.832

bc1_1053_2	07/03/24	27.855	0.937	0.139	26.551
	14/02/24	28.303	0.948	0.138	25.503
	14/03/24	26.177	0.937	0.166	26.009
	18/04/24	27.275	0.941	0.165	26.482
	21/03/24	26.171	0.936	0.171	25.548
bc1_1054	06/03/24	27.845	0.935	0.155	25.171
	13/03/24	27.179	0.94	0.157	25.22
	14/02/24	28.049	0.949	0.141	26.763
	16/04/24	28.185	0.954	0.155	27.457
	20/03/24	27.299	0.942	0.161	26.616
cs_1	05/03/24	27.062	0.951	0.141	26.595
	12/03/24	28.719	0.953	0.161	25.789
	13/02/24	30.234	0.968	0.132	27.926
	15/04/24	29.024	0.955	0.16	28.523
	19/02/24	28.769	0.961	0.129	28.194
	19/03/24	27.645	0.948	0.198	25.816
cs_2	05/03/24	28.764	0.95	0.144	24.98
	12/03/24	29.034	0.955	0.139	26.141
	13/02/24	27.263	0.953	0.141	26.799
	15/04/24	28.896	0.961	0.134	29.231
	19/02/24	29.998	0.96	0.117	26.05
	19/03/24	25.346	0.934	0.203	24.641
cs_2b_2eb_4_1	07/03/24	28.137	0.941	0.14	24.59
	14/02/24	29.419	0.963	0.124	26.11
	14/03/24	28.553	0.948	0.137	26.197
	18/04/24	27.072	0.939	0.168	26.475
	20/02/24	29.568	0.962	0.12	26.764
	21/03/24	27.047	0.937	0.156	26.583

cs_2d_2eb_1	07/03/24	28.218	0.953	0.136	26.676
	14/02/24	28.83	0.95	0.127	26.862
	14/03/24	28.35	0.953	0.145	26.251
	18/04/24	29.048	0.961	0.146	25.444
	20/02/24	26.942	0.936	0.145	27.45
	21/03/24	30.047	0.963	0.123	27.35
cs_2d_2eb_2	07/03/24	27.802	0.941	0.143	24.076
	14/02/24	28.79	0.95	0.128	25.718
	14/03/24	29.387	0.954	0.146	25.274
	18/04/24	28.044	0.956	0.159	27.438
	21/03/24	26.785	0.943	0.167	24.286
cs_2d_2eb_4_1	06/03/24	28.87	0.952	0.138	25.034
	13/03/24	28.033	0.947	0.157	24.993
	14/02/24	28.795	0.95	0.142	26.074
	16/04/24	29.84	0.959	0.146	25.512
	20/03/24	27.928	0.959	0.138	28.039
gru_da5j_1	05/03/24	30.065	0.956	0.112	27.902
	12/03/24	27.447	0.952	0.139	27.75
	13/02/24	28.442	0.955	0.129	25.292
	15/04/24	29.435	0.966	0.127	29.94
	19/02/24	29.581	0.96	0.131	28.131
	19/03/24	28.076	0.953	0.166	26.545
gru_da5j_2	05/03/24	28.619	0.952	0.132	26.531
	12/02/24	30.219	0.965	0.116	27.012
	12/03/24	28.776	0.952	0.141	27.659
	15/04/24	28.902	0.955	0.147	26.47
	19/02/24	29.471	0.958	0.118	28.611
	19/03/24	26.893	0.945	0.184	25.961

gru_da5j_3	05/03/24	30.323	0.965	0.11	28.351
	12/02/24	30.369	0.97	0.126	27.007
	12/03/24	28.398	0.961	0.143	27.081
	15/04/24	26.831	0.953	0.151	27.965
	19/02/24	31.109	0.972	0.114	28.251
	19/03/24	26.39	0.949	0.184	27.439
langdon_1	07/03/24	27.746	0.942	0.157	25.667
	14/02/24	27.799	0.944	0.146	26.668
	14/03/24	27.944	0.938	0.149	24.908
	18/04/24	25.785	0.926	0.186	21.901
	21/03/24	25.795	0.917	0.176	23.394
langdon_2	05/03/24	28.882	0.945	0.138	27.898
	12/02/24	28.517	0.95	0.131	26.265
	12/03/24	28.016	0.943	0.163	26.455
	15/04/24	28.119	0.943	0.142	24.667
	19/03/24	26.977	0.94	0.184	25.992
	20/02/24	28.857	0.949	0.116	27.269
langdon_3	07/03/24	28.868	0.952	0.132	26.198
	14/02/24	28.051	0.949	0.136	25.671
	14/03/24	27.762	0.943	0.159	25.138
	18/04/24	27.215	0.946	0.159	26.587
	21/03/24	27.536	0.94	0.152	24.756
langdon_4	05/03/24	27.025	0.938	0.139	24.353
	12/03/24	26.597	0.94	0.152	25.37
	13/02/24	28.436	0.95	0.138	26.367
	15/04/24	28.407	0.943	0.154	25.839
	19/03/24	25.871	0.931	0.192	26.389
	20/02/24	29.053	0.952	0.122	26.94

3.7 3DGS Undistorted Segmented Transforms

**Plant Name**	Date	PSNR MASK
bc1_1033_3	06/03/24	13.635
	13/02/24	13.199
	13/03/24	12.801
	16/04/24	14.368
	20/03/24	15.552
bc1_1050	06/03/24	12.283
	13/02/24	13.18
	13/03/24	13.715
	16/04/24	14.152
	20/03/24	13.934
bc1_1051	06/03/24	15.065
	13/03/24	14.693
	14/02/24	13.774
	16/04/24	15.071
	20/03/24	15.047
bc1_1052_2	06/03/24	13.731
	13/03/24	14.127
	14/02/24	13.717
	16/04/24	12.656
	20/03/24	15.187
bc1_1053_1	06/03/24	14.373
	13/03/24	
	14/02/24	14.069
	16/04/24	13.968
	20/02/24	14.325
	20/03/24	14.463

**Plant Name**	Date	PSNR MASK
bc1_1053_2	07/03/24	13.873
	14/02/24	12.75
	14/03/24	14.668
	18/04/24	14.689
	21/03/24	14.942
bc1_1054	06/03/24	13.406
	13/03/24	14.173
	14/02/24	13.002
	16/04/24	16.087
	20/03/24	15.343
cs_1	05/03/24	14.609
	12/03/24	14.24
	13/02/24	15.09
	15/04/24	16.386
	19/02/24	14.357
	19/03/24	10.917
cs_2	05/03/24	13.132
	12/03/24	13.062
	13/02/24	13.543
	15/04/24	16.025
	19/02/24	13.083
	19/03/24	10.892
cs_2b_2eb_4_1	07/03/24	12.058
	14/02/24	12.298
	14/03/24	12.524
	18/04/24	13.172
	20/02/24	13.844
	21/03/24	13.046

**Plant Name**	Date	PSNR MASK
cs_2d_2eb_1	07/03/24	13.169
	14/02/24	13.439
	14/03/24	13.399
	18/04/24	11.468
	20/02/24	14.417
	21/03/24	14.274
cs_2d_2eb_2	07/03/24	11.911
	14/02/24	12.671
	14/03/24	13.053
	18/04/24	14.257
	21/03/24	12.771
cs_2d_2eb_4_1	06/03/24	12.983
	13/03/24	13.284
	14/02/24	13.417
	16/04/24	12.62
	20/03/24	14.541
gru_da5j_1	05/03/24	14.989
	12/03/24	15.777
	13/02/24	11.855
	15/04/24	17.627
	19/02/24	14.699
	19/03/24	11.232
gru_da5j_2	05/03/24	14.547
	12/02/24	13.575
	12/03/24	13.929
	15/04/24	14.699
	19/02/24	14.764
	19/03/24	11.759

**Plant Name**	Date	PSNR MASK
gru_da5j_3	05/03/24	15.964
	12/02/24	14.967
	12/03/24	15.788
	15/04/24	14.762
	19/02/24	15.578
	19/03/24	11.249
langdon_1	07/03/24	13.026
	14/02/24	13.09
	14/03/24	12.868
	18/04/24	10.609
	21/03/24	12.112
langdon_2	05/03/24	14.842
	12/02/24	13.255
	13/03/24	14.452
	15/04/24	13.329
	19/03/24	11.405
	20/02/24	13.93
langdon_3	07/03/24	13.656
	14/02/24	12.715
	14/03/24	13.398
	18/04/24	15.632
	21/03/24	13.366
langdon_4	05/03/24	13.231
	12/02/24	13.845
	13/03/24	13.445
	15/04/24	15.223
	19/03/24	11.784
	20/02/24	13.715

3.8 3DGS Post-processed transforms

**Plant Name**	Date	PSNR MASK
bc1_1033_3	06/03/24	18.788
	13/02/24	19.626
	13/03/24	17.669
	16/04/24	17.994
	20/03/24	17.926
bc1_1050	06/03/24	16.821
	13/02/24	19.452
	13/03/24	17.004
	16/04/24	18.516
	20/03/24	17.675
bc1_1051	06/03/24	19.438
	13/03/24	18.273
	14/02/24	18.968
	16/04/24	18.701
	20/03/24	19.035
bc1_1052_2	06/03/24	18.487
	13/03/24	18.01
	14/02/24	18.395
	16/04/24	17.573
	20/03/24	18.667
bc1_1053_1	06/03/24	18.535
	13/03/24	10.299
	14/02/24	18.369
	16/04/24	17.89
	20/02/24	17.616
	20/03/24	17.232

**Plant Name**	Date	PSNR MASK
bc1_1053_2	07/03/24	19.06
	14/02/24	16.879
	14/03/24	18.631
	18/04/24	18.265
	21/03/24	17.961
bc1_1054	06/03/24	18.51
	13/03/24	17.905
	14/02/24	17.708
	16/04/24	20.051
	20/03/24	19.165
cs_1	05/03/24	20.328
	12/03/24	18.212
	13/02/24	18.969
	15/04/24	19.507
	19/02/24	18.819
	19/03/24	13.387
cs_2	05/03/24	17.406
	12/03/24	16.546
	13/02/24	18.185
	15/04/24	19.082
	19/02/24	17.801
	19/03/24	13.508
cs_2b_2eb_4_1	07/03/24	16.392
	14/02/24	17.201
	14/03/24	16.228
	18/04/24	18.545
	20/02/24	20.802
	21/03/24	17.009

**Plant Name**	Date	PSNR MASK
cs_2d_2eb_1	07/03/24	17.861
	14/02/24	18.307
	14/03/24	17.293
	18/04/24	15.294
	20/02/24	19.271
	21/03/24	16.749
cs_2d_2eb_2	07/03/24	16.264
	14/02/24	17.191
	14/03/24	16.58
	18/04/24	18.42
	21/03/24	16.242
cs_2d_2eb_4_1	06/03/24	19.025
	13/03/24	18.52
	14/02/24	18.491
	16/04/24	17.371
	20/03/24	17.837
gru_da5j_1	05/03/24	19.788
	12/03/24	18.825
	13/02/24	17.118
	15/04/24	21.172
	19/02/24	19.357
	19/03/24	14.563
gru_da5j_2	05/03/24	19.202
	12/02/24	17.715
	12/03/24	16.275
	15/04/24	18.033
	19/02/24	19.705
	19/03/24	14.522

**Plant Name**	Date	PSNR MASK
gru_da5j_3	05/03/24	21.32
	12/02/24	20.74
	12/03/24	20.07
	15/04/24	19.113
	19/02/24	19.654
	19/03/24	13.989
langdon_1	07/03/24	17.23
	14/02/24	17.885
	14/03/24	17.248
	18/04/24	14.932
	21/03/24	15.244
langdon_2	05/03/24	19.13
	12/02/24	18.095
	13/03/24	17.379
	15/04/24	18.476
	19/03/24	13.18
	20/02/24	19.27
langdon_3	07/03/24	18.129
	14/02/24	17.999
	14/03/24	17.309
	18/04/24	18.948
	21/03/24	18.311
langdon_4	05/03/24	18.005
	12/02/24	17.562
	13/03/24	18.941
	15/04/24	19.133
	19/03/24	13.307
	20/02/24	19.201

3.9 3DGS Colmap Transforms

**Plant Name**	Date	PSNR	SSIM	LPIPS	PSNR MASK
bc1_1033_3	20/03/24	27.579	0.939	0.183	24.212
bc1_1050	20/03/24	25.663	0.927	0.207	21.386
bc1_1051	20/03/24	26.273	0.924	0.199	22.706
bc1_1052_2	20/03/24	30.267	0.948	0.103	26.546
bc1_1053_1	20/03/24	27.127	0.932	0.19	22.312
bc1_1053_2	21/03/24	26.986	0.929	0.194	22.93
bc1_1054	20/03/24	25.173	0.918	0.223	21.223
cs_1	19/03/24	24.636	0.916	0.251	21.306
cs_2	19/03/24	23.903	0.907	0.25	21.757
cs_2b_2eb_4_1	21/03/24	25.85	0.915	0.223	21.264
cs_2d_2eb_1	21/03/24	28.094	0.943	0.179	22.901
cs_2d_2eb_2	21/03/24	26.839	0.936	0.194	21.135
cs_2d_2eb_4_1	20/03/24	28.046	0.948	0.175	23.079
gru_da5j_1	19/03/24	26.67	0.936	0.192	24.184
gru_da5j_2	19/03/24	25.862	0.928	0.212	23.192
gru_da5j_3	19/03/24	26.27	0.939	0.196	24.474
langdon_1	21/03/24	24.098	0.895	0.252	19.828
langdon_2	19/03/24	19.447	0.871	0.368	13.841
langdon_3	21/03/24	23.08	0.898	0.267	17.648
langdon_4	19/03/24				22.228

3.10 Colmap Transform ATE

**Plant Name**	Date	ATE (m)
bc1_1033_3	20/03/24	0.00046
bc1_1050	20/03/24	0.00095
bc1_1051	20/03/24	0.00062
bc1_1052_2	20/03/24	-
bc1_1053_1	20/03/24	0.00036
bc1_1053_2	21/03/24	0.00056
bc1_1054	20/03/24	0.00073
cs_1	19/03/24	-
cs_2	19/03/24	-
cs_2b_2eb_4_1	21/03/24	0.00057
cs_2d_2eb_1	21/03/24	0.00044
cs_2d_2eb_2	21/03/24	0.00049
cs_2d_2eb_4_1	20/03/24	0.00041
gru_da5j_1	19/03/24	-
gru_da5j_2	19/03/24	-
gru_da5j_3	19/03/24	-
langdon_1	21/03/24	0.0047
langdon_2	19/03/24	0.00135
langdon_3	21/03/24	0.00094
langdon_4	19/03/24	-

3.11 Ground Truth to Point Cloud

**Plant Name**	Date	GT → 3DGS (m)	GT → NeRF (m)	GT → MVS (m)	GT → SfM (m)
bc1_1033_3	20/03/24	0.001971	0.001911	0.001756	0.006802
bc1_1050	20/03/24	0.001685	0.001857	0.00151	0.006507
bc1_1051	20/03/24	0.001079	0.001542	0.001717	0.007274
bc1_1052_2	20/03/24	0.002226	0.002696	0.00218	0.008315
bc1_1053_1	20/03/24	0.002264	0.002568	0.00266	0.00657
bc1_1053_2	21/03/24	0.00281	0.002815	0.002719	0.007265
bc1_1054	20/03/24	0.001855	0.002055	0.002308	0.007882
cs_1	19/03/24	0.001072	0.001437	0.001311	0.007865
cs_2	19/03/24	0.001362	0.002022	0.002784	0.007084
cs_2b_2eb_4_1	21/03/24	0.001909	0.002024	0.002617	0.007078
cs_2d_2eb_1	21/03/24	0.004465	0.001934	0.004028	0.00748
cs_2d_2eb_2	21/03/24	0.002225	0.001243	0.001906	0.005928
cs_2d_2eb_4_1	20/03/24	0.001352	0.00149	0.002194	0.007226
gru_da5j_1	19/03/24	0.000982	0.001298	0.002118	0.005914
gru_da5j_2	19/03/24	0.001451	0.003362	0.002327	0.006934
gru_da5j_3	19/03/24	0.000945	0.001326	0.001705	0.00579
langdon_1	21/03/24	0.002902	0.001412	0.002376	0.008482
langdon_2	19/03/24	0.001766	0.001959	0.001937	0.007391
langdon_3	21/03/24	0.002063	0.002233	0.004097	0.008664
langdon_4	19/03/24	0.001857	0.001662	0.002153	0.008092

3.12 Point Cloud to Ground Truth

**Plant Name**	Date	3DGS → GT (m)	NeRF → GT (m)	MVS → GT (m)	SfM → GT (m)
bc1_1033_3	20/03/24	0.006609	0.007993	0.008602	0.01096
bc1_1050	20/03/24	0.008178	0.011087	0.011922	0.016873
bc1_1051	20/03/24	0.007026	0.007732	0.008144	0.013809
bc1_1052_2	20/03/24	0.005929	0.006466	0.008066	0.012023
bc1_1053_1	20/03/24	0.010055	0.012593	0.014087	0.015815
bc1_1053_2	21/03/24	0.009713	0.011609	0.014689	0.016868
bc1_1054	20/03/24	0.01224	0.020521	0.018106	0.020187
cs_1	19/03/24	0.0059	0.008965	0.009161	0.013098
cs_2	19/03/24	0.004019	0.00626	0.004939	0.008701
cs_2b_2eb_4_1	21/03/24	0.010837	0.012416	0.014303	0.014311
cs_2d_2eb_1	21/03/24	0.018498	0.018614	0.021241	0.022039
cs_2d_2eb_2	21/03/24	0.017273	0.018278	0.021945	0.021498
cs_2d_2eb_4_1	20/03/24	0.012352	0.013382	0.01624	0.019115
gru_da5j_1	19/03/24	0.005855	0.006931	0.006739	0.009074
gru_da5j_2	19/03/24	0.005433	0.024093	0.007323	0.011381
gru_da5j_3	19/03/24	0.006316	0.007675	0.007238	0.00979
langdon_1	21/03/24	0.02564	0.023397	0.028023	0.028134
langdon_2	19/03/24	0.004694	0.00605	0.005791	0.01059
langdon_3	21/03/24	0.00813	0.008502	0.011698	0.013872
langdon_4	19/03/24	0.005136	0.005712	0.007203	0.011557

3.13 Ground Truth to Point Cloud with Segmented Pot

**Plant Name**	Date	GT → 3DGS (m)	GT → NeRF (m)	GT → MVS (m)	GT → SfM (m)
bc1_1033_3	20/03/24	0.00115	0.0018	0.001621	0.006508
bc1_1050	20/03/24	0.000629	0.001395	0.001231	0.006041
bc1_1051	20/03/24	0.000814	0.001426	0.001512	0.007121
bc1_1052_2	20/03/24	0.019623	0.002494	0.0021	0.006553
bc1_1053_1	20/03/24	0.001574	0.002345	0.002357	0.006443
bc1_1053_2	21/03/24	0.00175	0.002706	0.002432	0.007093
bc1_1054	20/03/24	0.001109	0.001869	0.001826	0.007746
cs_1	19/03/24	0.000706	0.001443	0.001238	0.005922
cs_2	19/03/24	0.001047	0.00189	0.00171	0.00657
cs_2b_2eb_4_1	21/03/24	0.00108	0.001766	0.00236	0.006907
cs_2d_2eb_1	21/03/24	0.001444	0.001959	0.001659	0.007141
cs_2d_2eb_2	21/03/24	0.000574	0.000961	0.001165	0.005025
cs_2d_2eb_4_1	20/03/24	0.000906	0.001429	0.001536	0.005482
gru_da5j_1	19/03/24	0.000729	0.001106	0.001354	0.005159
gru_da5j_2	19/03/24	0.000869	0.001628	0.001567	0.0063
gru_da5j_3	19/03/24	0.000959	0.001724	0.001328	0.004599
langdon_1	21/03/24	0.000762	0.001027	0.00131	0.008086
langdon_2	19/03/24	0.000909	0.001928	0.001505	0.007103
langdon_3	21/03/24	0.001215	0.001942	0.002692	0.007741
langdon_4	19/03/24	0.00074	0.001427	0.001305	0.006765

3.14 Point Cloud to Ground Truth with Segmented Pot

**Plant Name**	Date	3DGS → GT (m)	NeRF → GT (m)	MVS → GT (m)	SfM → GT (m)
bc1_1033_3	20/03/24	0.0064	0.00676	0.008359	0.010584
bc1_1050	20/03/24	0.008035	0.010932	0.011986	0.01737
bc1_1051	20/03/24	0.007005	0.007001	0.008082	0.013027
bc1_1052_2	20/03/24	0.005677	0.00567	0.007479	0.011841
bc1_1053_1	20/03/24	0.009917	0.011895	0.013964	0.015936
bc1_1053_2	21/03/24	0.009466	0.010977	0.014534	0.016994
bc1_1054	20/03/24	0.011548	0.013341	0.017305	0.020164
cs_1	19/03/24	0.005442	0.007291	0.008936	0.013256
cs_2	19/03/24	0.003685	0.003796	0.004372	0.007973
cs_2b_2eb_4_1	21/03/24	0.010799	0.012234	0.014466	0.015695
cs_2d_2eb_1	21/03/24	0.018699	0.019995	0.025097	0.025716
cs_2d_2eb_2	21/03/24	0.017396	0.018637	0.022568	0.023707
cs_2d_2eb_4_1	20/03/24	0.009492	0.013809	0.016852	0.020841
gru_da5j_1	19/03/24	0.005367	0.005176	0.006372	0.008573
gru_da5j_2	19/03/24	0.005154	0.005778	0.007125	0.011436
gru_da5j_3	19/03/24	0.006349	0.006098	0.006774	0.008168
langdon_1	21/03/24	0.025764	0.023569	0.028662	0.030283
langdon_2	19/03/24	0.004419	0.004631	0.005715	0.010733
langdon_3	21/03/24	0.009244	0.008288	0.011513	0.013683
langdon_4	19/03/24	0.004987	0.004754	0.007081	0.011428
