# Supplementary material for: High-fidelity wheat plant reconstruction using 3D Gaussian splatting and neural radiance fields
Source: Gigascience. 2025 Mar 26;14:giaf022. doi: 10.1093/gigascience/giaf022 (PMC11945317; doi:10.1093/gigascience/giaf022)
Supplement: giaf022_GIGA-D-24-00315_Original_Submission [file giaf022_giga-d-24-00315_original_submission.pdf]

GigaScience

High-fidelity Wheat Plant Reconstruction using 3D Gaussian Splatting and Neural Radiance Fields

--Manuscript Draft--

**Manuscript Number:**	GIGA-D-24-00315
**Full Title:**	High-fidelity Wheat Plant Reconstruction using 3D Gaussian Splatting and Neural Radiance Fields
**Article Type:**	Research
**Funding Information:**	Doctoral Training Centre in Artificial Intelligence — Mr Lewis Anthony Gordon Stuart<br>Graduate Centre for International Agriculture — Mr Jack Walker
**Abstract:**	3D digital twins offer numerous advantages over traditional 2D approaches by enabling automated phenotyping, and the extraction of key crop characteristics. Conventional 3D reconstruction techniques often produce sparse or noisy representations of plants using software, or are expensive to capture in hardware. Recently, view synthesis models have been developed that can generate detailed 3D scenes, and even 3D models, from only RGB images and camera poses. These models offer unparalleled accuracy, but are currently data hungry, requiring large numbers of views with very accurate camera calibration. In this study we present a view synthesis dataset comprising 20 individual wheat plants captured across 6 different time frames over a 15-week growth period. We develop a camera capture system using two robotic arms combined with a turntable, controlled by a re-deployable and flexible image capture framework. We trained each plant instance using two recent view synthesis models: 3D Gaussian Splatting (3DGS) and Neural Radiance Fields (NeRF). Our results show that both 3DGS and NeRF produce high-fidelity reconstructed images of a plant subject from views not captured in the initial training sets. We also show that these approaches can be used to generate accurate 3D representations of these plants as point clouds, with 1.91mm and 1.89mm average accuracy compared with a handheld scanner for 3DGS and NeRF respectively. We believe that these new methods will be transformative in the field of 3D plant phenotyping, plant reconstruction and active vision. To further this cause, we release all robot configuration and control software alongside our extensive multi-view dataset. We also release all scripts necessary to train both 3DGS and NeRF, all trained models, and final 3D point representations
**Corresponding Author:**	Lewis Anthony Gordon Stuart<br>University of Nottingham School of Computer Science<br>Nottingham, UNITED KINGDOM
**Corresponding Author Secondary Information:**	
**Corresponding Author's Institution:**	University of Nottingham School of Computer Science
**Corresponding Author's Secondary Institution:**	
**First Author:**	Lewis Anthony Gordon Stuart
**First Author Secondary Information:**	
**Order of Authors:**	Lewis Anthony Gordon Stuart
	Darren M Wells
	Jonathan A Atkinson
	Simon Castle-Green
	Jack Walker
	Michael P Pound
**Order of Authors Secondary Information:**	

Powered by Editorial Manager® and ProduXion Manager® from Aries Systems Corporation

Additional Information:	
**Question**	**Response**
Are you submitting this manuscript to a special series or article collection?	No
**Experimental design and statistics**<br><br>Full details of the experimental design and statistical methods used should be given in the Methods section, as detailed in our Minimum Standards Reporting Checklist. Information essential to interpreting the data presented should be made available in the figure legends.<br><br>Have you included all the information requested in your manuscript?	Yes
**Resources**<br><br>A description of all resources used, including antibodies, cell lines, animals and software tools, with enough information to allow them to be uniquely identified, should be included in the Methods section. Authors are strongly encouraged to cite Research Resource Identifiers (RRIDs) for antibodies, model organisms and tools, where possible.<br><br>Have you included the information requested as detailed in our Minimum Standards Reporting Checklist?	Yes
**Availability of data and materials**<br><br>All datasets and code on which the conclusions of the paper rely must be either included in your submission or deposited in publicly available repositories (where available and ethically appropriate), referencing such data using a unique identifier in the references and in the “Availability of Data and Materials” section of your manuscript.	Yes

Have you have met the above requirement as detailed in our Minimum Standards Reporting Checklist?	

Manuscript

Click here to access/download;Manuscript;Reconstructing_Plants_using_Ne

GigaScience, 2024, 1–14

doi: xx.xxxx/xxxx
Manuscript in Preparation
Research

RESEARCH

High-fidelity Wheat Plant Reconstruction using 3D Gaussian Splatting and Neural Radiance Fields

Lewis A G Stuart[1], Darren M Wells[2], Jonathan A Atkinson[2], Simon Castle-Green[1], Jack Walker[2] and Michael P Pound[1]

[1]School of Computer Science, University of Nottingham, UK and [2]School of Biosciences, University of Nottingham, UK

Abstract

3D digital twins offer numerous advantages over traditional 2D approaches by enabling automated phenotyping, and the extraction of key crop characteristics. Conventional 3D reconstruction techniques often produce sparse or noisy representations of plants using software, or are expensive to capture in hardware. Recently, view synthesis models have been developed that can generate detailed 3D scenes, and even 3D models, from only RGB images and camera poses. These models offer unparalleled accuracy, but are currently data hungry, requiring large numbers of views with very accurate camera calibration. In this study we present a view synthesis dataset comprising 20 individual wheat plants captured across 6 different time frames over a 15-week growth period. We develop a camera capture system using two robotic arms combined with a turntable, controlled by a re-deployable and flexible image capture framework. We trained each plant instance using two recent view synthesis models: 3D Gaussian Splatting (3DGS) and Neural Radiance Fields (NeRF). Our results show that both 3DGS and NeRF produce high-fidelity reconstructed images of a plant subject from views not captured in the initial training sets. We also show that these approaches can be used to generate accurate 3D representations of these plants as point clouds, with 1.91mm and 1.89mm average accuracy compared with a handheld scanner for 3DGS and NeRF respectively. We believe that these new methods will be transformative in the field of 3D plant phenotyping, plant reconstruction and active vision. To further this cause, we release all robot configuration and control software alongside our extensive multi-view dataset. We also release all scripts necessary to train both 3DGS and NeRF, all trained models, and final 3D point representations. Our dataset can be accessed via **[Temporary Review URL].** Our software can be accessed via **this link.**

Key words: 3D Gaussian Splatting, 3DGS, Neural Radiance Fields, NeRF, View Synthesis, Machine Learning, 3D Reconstruction, Digital Twin, Robotics, Phenotyping, Imaging

Introduction

In recent years, 3D reconstruction of plants has become an important tool in plant phenotyping pipelines. Generating a 3D digital twin of a plant subject facilitates the extraction of key traits, and simplifies the analysis of complex plant structures. The ability to accurately capture these traits in 3D provides valuable information for determining a plant's growth rate, health and stress factors [1]. Plant leaves (and the canopies they form) are inherently 3D structures, and factors such as leaf curling, rolling, and occlusion lead to inaccuracies when determining parameters from 2D images [2]. Determining this information is critical in assessing the overall validity of the crop and identifying potential alterations needed to improve yield.

Reconstruction of plants in 3D has typically been solved through either hardware or software approaches. Hardware systems based on Light Detection and Ranging (LiDAR) use time of flight light measurement to accurately measure the distance between the sensor and evenly spaced points within a scene. These devices are capable of highly accurate representations of plants [3]. However, they are

Compiled on: August 16, 2024.
Draft manuscript prepared by the author.

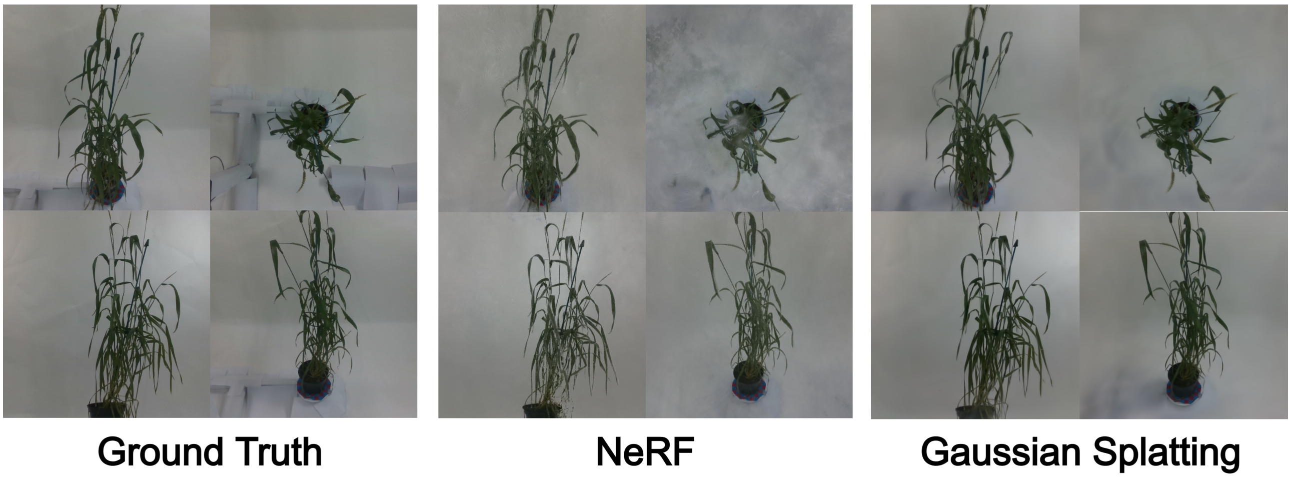

Figure 1. Showcase of some of the rendered images for one the plants in our view synthesis dataset (bc1_1054: 13-03-24). Left column displays the captured groundtruth images. Middle column contains images rendered after training with standard RGB images, with transforms calculated after bundle adjustment, on the nerfacto NeRF model. Right column displays images rendered after training with undistorted RGB images, with transforms calculated after bundle adjustment, on the splatfacto 3DGS model.

often expensive to acquire and require expertise to operate. Lower cost software-based methods such as Structure from Motion (SfM) operate by generating a point cloud from a series of 2D images of a plant [4]. Points are triangulated across views to estimate their position in 3D space. Modern SfM approaches are efficient, and require little hardware beyond image capture devices. However, these methods often produce sparse representations of a plant, and may struggle to capture the fine detail necessary for accurate phenotyping. Both LiDAR and SfM generate point representations of scenes, rather than continuous surface representations, which may be required depending on the phenotyping task.

Recent progress in deep learning has lead to the development of view synthesis models, which offer exciting new opportunities for 3D plant phenotyping. These models are trained from 2D images of a scene, and are commonly used to generate new views of objects not included in the initial training set. However, they can also be used to extract volumetric representations of plants, point clouds, and continuous representations, potentially enabling step change in 3D plant phenotyping.

Neural Radiance Fields (NeRF) [5], popularised in 2020, utilise a neural network and volumetric rendering to generate a continuous representation of a scene. 3D Gaussian Splatting (3DGS) [6], projects a series of coloured ellipsoids into a scene and employs gradient descent to optimise their positions and shape. These methods implicitly generate a 3D representation of a scene, and while most literature focuses on generating new views, these techniques can be utilised for 3D reconstruction of plants. There has been limited research on the use of view synthesis models for plant shoot reconstruction, these are emerging technologies, but primarily there is finite availability of large multi-view datasets required to exploit these methods.

In this paper we introduce an extensive multi-view dataset of wheat plants, and demonstrate the state-of-the-art performance of view synthesis models on both novel view synthesis, and 3D plant reconstruction. Our dataset comprises 20 wheat plants captured over six time frames. For each plant, and at each time point, we train high quality models using both NeRF and 3DGS approaches, which we use for novel view synthesis and full 3D reconstruction of each plant. Our dataset aims to serve as a baseline for evaluating different view synthesis models on plants, and can also be used to develop and test a large number of downstream tasks related to 3D phenotyping, such as extraction of 3D traits, surface reconstruction, canopy light modelling, and next-best-view problems. We provide straightforward scripts and thorough documentation to assist other researchers in executing our trained view synthesis models locally.

We utilise wheat plants in this paper as these species are one of the most widely produced crops worldwide, accounting for 20% of human calories as well as providing vital proteins, minerals and vitamins for a healthy human diet [7]. The global average annual yield increase of wheat is 0.9%, however the predicted increase in demand is 2.4% [8]. Wheat plants offer substantial challenges compared to typical scenes used to evaluate view synthesis models. These include multi-layered occlusions and narrow leaf structure, making them an appropriate target for evaluating the capabilities of different 3D reconstruction methods.

Each wheat plant was captured from multiple views using a dual-robot imaging setup, enabling the capture of a wide range of views and good coverage of each plant. Our robot setup also facilitates logging of camera positions in metric units, ensuring that the measurements recorded on the reconstructed plants from either NeRF or 3DGS are equivalent to their real-life counterpart. We use two robots to capture the widest possible range of views, however our approaches are compatible with single robot or other systems.

We validate the accuracy of novel view synthesis by comparing rendered images against unseen views of the real plants. We find that both approaches offer excellent render quality, with 3DGS offering the best performance. Figure 1 shows rendered images of a wheat plant that was reconstructed using both of these methods.

To validate the accuracy of the 3D reconstructions produced by both NeRF and 3DGS, we manually capture several of the imaged plants using a handheld structured light scanner (Handheld 3D Scanner, Einstar). We compare our model reconstructions against this ground truth and we find that the average error between the reconstruction and ground truth scan was only 1.91mm for 3DGS and 1.89mm for NeRF. In contrast, a point cloud generated using a standard Structure from Motion (SfM) framework had an average error of 11.35 mm.

We conclude by discussing the potential use cases and implications of these new technologies on the field of plant phenotyping. We release the full dataset of 112 plant instances and over 35,000 RDB-D images, all trained models, camera parameters, computed 3D representations and ground truth scans. We also release our image capture framework, compatible with any robot that supports the Robot Operating System (ROS) [9]. This framework can generate new datasets ready for training on any standardised view synthesis model. We also provide our robot configuration files, enabling convenient replication of the setup in any environment. If required, this same setup can be deployed virtually using the Gazebo robotics simulator library [10], enabling the capture of synthetic plant models.

In summary, our main contributions are:

- A new view synthesis dataset of 112 wheat plant instances. This dataset can be used to develop and train new view synthesis and 3D modelling approaches that target complex plant topology, or to develop and evaluate new 3D phenotyping approaches. This dataset can be accessed via **this link.**
- A dual-robot image capture setup applicable to a variety of robot manipulators and image capture devices. Our system is designed such that all 3D models exist in a metric co-ordinate system, and so phenotyping measurements are directly mapped to the original plants.
- Experiments demonstrating the benefits and drawbacks of view synthesis models compared to standardised methods for 3D plant reconstruction, and a detailed comparison of the strengths and weakness of both NeRF and 3DGS approaches for plant phenotyping.
- All of our robot configuration files, view capture pipeline and 3D Gaussian Splatting to point cloud conversion codebase can be found on our Github Repository using **this link.**

Background

3D Plant Representations

Point clouds represent one of the more fundamental forms of 3D representation, wherein an object's surface is encoded as a set of points with a 3D position, and optionally an RGB colour value. This data representation has become popular for downstream phenotyping tasks, such as leaf/stem segmentation [11] or estimating branch angles [12]. Additionally, several software packages have been developed to automatically extract phenotypic traits, such as plant height, projected leaf area and convex hull volume, from point clouds of various species [13, 14]. Consequently, many 3D plant datasets have been developed that consist of point clouds of plant structures that can be utilised for phenotyping [15]. Despite this, point clouds are often impacted by erroneous outliers, frequently necessitating the application of post-processing algorithms to denoise the reconstructed data. In addition, point clouds provide no explicit surface representation.

Voxel-grids constitute another widely adopted representation method, in which the 3D environment is divided into a grid of voxels, each constituting distinct colour values in a predefined space. This representation has demonstrated its efficacy in various phenotyping tasks, including the assessment of holistic and component characteristics [16], as well as the computation of leaf angles [17]. While voxel grids offer good noise robustness, they often sacrifice fine-grained surface detail when compared to point clouds, due to their fixed grid resolution.

Meshes represent an alternative 3D representation approach that involves the reconstruction of plant surfaces through the use of polygons. While meshes have occasionally been utilised for phenotyping [18], their additional complexity often sees their use in physical simulations rather than standardised phenotyping practices.

A drawback common across current 3D representations is that the quality of the reconstruction is reliant on challenging data acquisition and 3D reconstruction methods. Image based methods often struggle to reconstruct the complex topology of plants, and as such these 3D structures are often sparse, inadequately capturing the intricacies of their real-life counterparts.

Recent developments in deep learning have led to several new formats for representing 3D structures. One important development has been the adoption of implicit representations, which model plants as a continuous structure, rather than at discrete positions such as points or voxels. Typically this is achieved using a deep neural network trained to represent the plant and sample from any position. These representations circumvent the limitations of traditional 3D structures, as the accuracy of the reconstruction depends solely on the resolution of the input data and the complexity of the reconstructed model. While these models offer potentially unlimited sampling resolution, in practice they can be challenging to use to extract plant traits. All existing phenotyping pipelines assume a discrete representation in a form above, and further research is required to explore the potential of these exiting new models.

Another recent development has been in 3D Gaussian representations, which are conceptually similar to point clouds. This representation is formed of a series of 3D Gaussian functions projected into 3D space, with their shape and colour being optimised to effectively model the plant. Intuitively, these can be thought of as a coloured or semi-transparent ellipsoids. Many ellipsoids can be positioned and shaped to represent a dense reconstruction of the surfaces in the scene.

Overall, these modern representations circumvent the limitations of traditional 3D structures, as the accuracy of the reconstruction depends more on the resolution of the input data and the complexity of the reconstruction model. We refer interested readers to [19] for a detailed discussion of 3D representations and reconstruction approaches for plants and trees.

3D Reconstruction Methods

Reconstruction methods are typically split into two categories. Active approaches, in which light emitters are utilised to retrieve information about a 3D scene [20], and passive approaches, in which equipment, typically RGB cameras, are employed to receive light that can be used to extract 3D information of an environment [21].

A common approach to active 3D reconstruction involves the utilisation of 3D laser scanners/LiDAR cameras. These devices determine distances from their optical centers by measuring the time it takes for emitted light to reach a specific point on a surface within an environment. Costly industrial-grade scanners are capable of generating highly detailed 3D point clouds within a defined area [22]. Where cost is prohibitive, low cost depth cameras have also been utilised for effective plant reconstruction [23]. While these technologies excel in rapid data acquisition, they do have limitations, including restricted coverage and difficulty capturing dense or topologically complex regions. As a result, these scanners are not optimally suited for capturing plants characterized by intricate detail (e.g. thin leaves, small branches, spikes)[24].

Two-view stereo is one of the early forms of passive 3D reconstruction and requires only two RGB cameras. Conversion from 2D to 3D involves triangulation of pixel data based on registered camera positions. This process offers rapid and effective retrieval of plant characteristics, but typically yields sparse reconstructions of plant models [25].

Multi-view stereo (MVS) extends this approach by introducing multiple cameras into the image acquisition process. Consequently, this approach can generate dense 3D point clouds with impressive high point-position accuracy. MVS has been shown to reconstruct plant canopies with high accuracy [26, 27], and has become popular as an initial step in phenotyping pipelines [28, 29]. Nevertheless, this approach can incur a high computational cost compared to active reconstruction methods, and the accuracy of the 3D point cloud is directly reliant on the precision of the registered cameras position and rotation.

Structure from Motion (SfM) extends MVS, where camera positions are not known prior to image acquisition. Hence, SfM involves preliminary steps such as point extraction, matching, and triangulation to accurately determine camera positions before proceeding to dense reconstruction. SfM has been shown to work effectively for reconstruction of plant geometry [30] and trees [31]. However, these process require accurate feature matching, which is challenging on plants where texture is often repetitive, and they exhibit

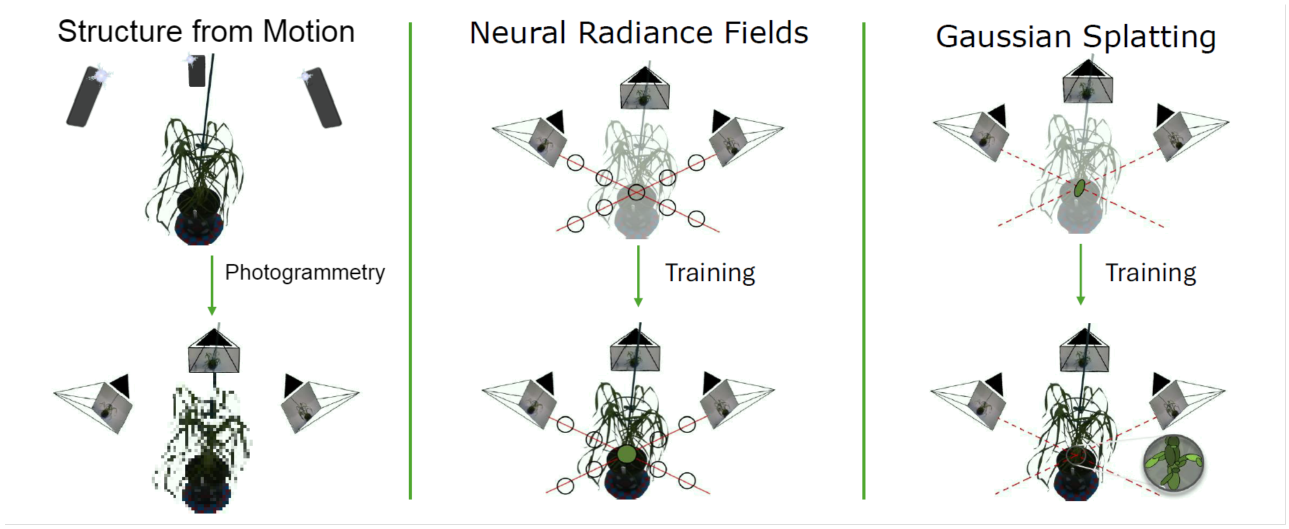

Figure 2. A visual depiction of the basics of SfM, NeRF and 3DGS. In SfM, data is captured and a point cloud is generated using photogrammetry. Both NeRFs and 3DGS initially have an empty scene and are trained on the captured images with associated camera poses. In NeRFs, ray marching is utilised to interact with the scene at specific locations and these queried points are optimised to reconstruct the plant correctly. 3DGS projects a set of initial Gaussians into the scene, and overtime these Gaussians are optimised to better represent the plant in 3D space.

complex shape and self-occlusion. Furthermore, while the process of camera calibration in SfM makes image acquisition more flexible, this commonly results in 3D scenes that don't correspond to real metric or known units. This means that scenes must be manually scaled or otherwise registered later by some additional process. Without such a registration, key phenotyping characteristics such as plant height, leaf area, and convex hull would be inaccurate.

Ultimately, the choice of 3D reconstruction technique depends on the specific plant being captured, the available capture equipment and the desired processing time [32]. Additional information on various standardized 3D plant reconstruction methods may be found in [33] and [34].

View Synthesis Models

View synthesis is the process of generating novel images of an environment from a specific viewpoint not included in the set of prior images. Although view synthesis models have seen limited uptake for plant phenotyping so far, we foresee increased use in the future, better enabling applications such as next best view and extracting phenotypic traits from multiple views. View synthesis models only require a set of 2D images and a series of 'transforms', which define the intrinsic and extrinsic camera parameters, similar to the requirements to generate a point cloud using MVS.

Neural Radiance Fields (NeRF) [5] are a proposed solution to view synthesis, producing novel views that have been seen to far surpass previous methods, even on complex scenes. NeRF employs volumetric rendering techniques that utilise a neural network to predict density and colour at positions in the environment. Consequently, NeRFs are a promising candidate for 3D reconstruction from images.

Several impressive extensions have been proposed for NeRF, such as improved ray-casting in Mip-NeRF 360 [35][36] and hash-encoding in Instant-NGP [37]. NeRFStudio, a popular view synthesis framework, introduced NeRFacto, which incorporates the most successful architectural improvements from various NeRF models [38].

While NeRFs produce extremely impressive reconstruction results, utilising a neural network to encode the entire scene leads to slow rendering times and challenges that arise with handling implicit data.

At the time of writing, there has been limited research utilising NeRFs for 3D plant reconstruction. Firstly, it has been shown that plants can be reconstructed in high accuracy by comparing the NeRF representation to a captured ground truth scan, yielding and impressive result of only 10mm error for single indoor maize plant [39]. Other studies have extended this by evaluating NeRF on multiple indoor and outdoor plants [40], confirming similar results, with NeRF representations trained using NeRFacto producing the most precise 3D representations.

It has also been demonstrated that NeRF can reconstruct a variety of different types of fruit with high accuracy [41], including peppers, tomatoes and pitahaya. This shows that NeRFs are capable of effectively reconstructing plants with complex structures, materials and occlusions.

Other studies focused more on applying NeRF directly to phenotyping problems. PeanutNeRF [42] accomplished peanut pod detection by creating a 3D implicit representation of the peanut plant using a NeRFacto model, and using a segmentation and bounding box estimation pipeline to identify areas in the scene that encapsulate each individual peanut pod. Another study deployed a portable robot with an attached camera and scanner in a greenhouse to reconstruct peppers [43]. A segmentation algorithm was developed to identify these peppers from a trained NeRF model and extract phenotypic traits, such as width and height. These measurements could be accurately calculated since the robot was calibrated in metric units.

While NeRF models are capable of high quality reconstructions, replicating these results can be challenging, and captured datasets are either limited or have not been made public.

3D Gaussian Splatting (3DGS) [6] represents another approach to view synthesis, in which the scene is populated with 3D Gaussians which encode colour and density at significant positions within an environment. Gradient descent is used to optimise each of the Gaussians in the scene to fit the environment correctly. Culling algorithms are incorporated to ensure redundant Gaussians are removed from the scene.

There have been several proposed improvements to 3DGS, such as incorporating anchor points [44] and improved pruning functions [45], but so far the process is still in its infancy. NeRFStudio has released their own 3DGS model known as Splatfacto, which can produce high-quality reconstructions. Unlike NeRF, Gaussians are an explicit representation of the scene, which makes them more flexible to handle and this is why 3DGS can perform real-time rendering. The differences between these two methods are visualised in Figure 2.

To our knowledge, there has been no previous application of 3DGS to plant shoot reconstruction.

Plant Imaging Setups

Numerous plant image capture setups have been proposed for 3D reconstruction, including those noted in the papers above. Some involve gantry systems equipped with robotic arms designed to capture views from various angles around a plant subject [46]. Simpler setups utilise a rotating board to reposition two cameras around a plant subject [47], while other systems use a turntable to rotate the plant subject rather than maneuvering the cameras [48, 49, 29, 50, 51]. Many existing installations are challenging to re-deploy into new locations due to a lack of available configuration and software. Others with limited range of movement are incapable of capturing the full range of views required for effective 3D Reconstruction using view synthesis models.
Here, we utilise two Universal Robots UR5 robotic arms, along with a turntable, to capture the broad range of necessary views for reconstruction of wheat plants. UR5 robotic arms have found application in various phenotyping contexts, such as leaf scanning [52], plant grasping/pruning [53, 54] and next-best view planning [55], primarily due to ease of use and moderate reach.

Methodologies

Robotic Imaging Setup

View synthesis models, such as NeRF and 3DGS, benefit from a large number of views of the scene. Ideal imaging setups would capture images at equidistant intervals around an object being imaged, with as much of the object as possible in view within each frame. Our robot capture setup is designed with these features in mind, while remaining easily reconfigurable and adaptable to other plant species or installation locations.
We experimented with a single UR5 using an Intel Realsense D435i camera mounted at the Tool Center Point (TCP). However, we found that a single robot failed to provide adequate reach to obtain the majority of required views, particularly across the full range of 360 degrees around the plant.
To address this limitation, we integrated a Zaber X-RST stepper motor turntable, which offers a full 360°rotation range with 0.16°unidirectional accuracy. The turntable's ability to rotate to any angle allowed us to focus only on viewpoints along the x and z axis, with the y axis being fixed. We set the turntable speed to precisely 3°per second to minimise plant micro-movements during rotation while maintaining efficient rotation time. The turntable was centered at the origin (0,0,0) of our robot's coordinate system, allowing straightforward calculations of transform positions relative to the turntable.
Despite this we found that some views, particularly those above the plant, remained challenging to reach for a single robot. We therefore employed a second UR5 robotic arm mounted on a separate pedestal, elevated above the base of the other robot, which increased our range of potential views. The base of the first UR5 was positioned at coordinates (0.35m, -0.45m, 1.3m), while the base of the second UR5 was located at coordinates (0.85m, 0.45m, 0.85m). Our coordinate system adhered to the standard Robot Operating System (ROS) convention, where the positive Z-axis points upward and values are in metric units. Each UR5 base was mounted on a customised pedestal, strategically positioned to provide access to views ranging from 0.3 meters to 1.5 meters from the turntable origin. These choices ensured that our setup could capture a wide range of views for a variety of different plant sizes. Each iteration of our setup, along with a showcase of reachable views, is depicted in Figure 3.

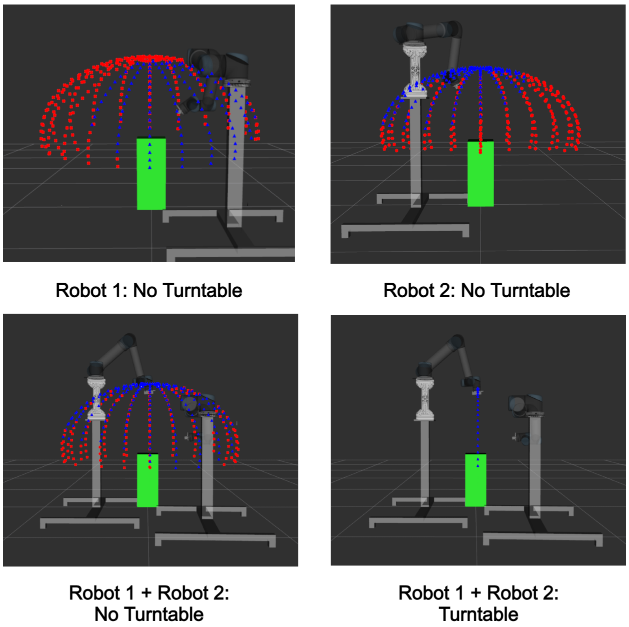

Figure 3. Image showing the effectiveness of the different considered setups. Blue points represent positions around the plant that could be reached, while red points represent positions that were unreachable. These points were generated for a plant with a height of 0.5m and a capture radius of 0.75m. The different setups are: 1) A setup consisting of only 1 UR5 arm in position (0.35m, -0.45m, 1.3m). 2) A setup consisting of only 1 UR5 arm in position (0.85m, 0.45m, 0.85m). 3) A setup with two UR5 arms in positions (0.35m, -0.45m, 1.3m) and (0.85m, 0.45m, 0.85m). 4) Our final setup with two UR5 arms in positions (0.35m, -0.45m, 1.3m) and (0.85m, 0.45m, 0.85m) and an accompanying turntable

To control the UR5 robotic arms, we installed ROS Noetic Ninjemys and developed a custom dual UR5 MoveIt [56] package, enabling parallel path planning for both arms. To facilitate this, we created a custom Unified Robot Description Format (URDF) file with joints extending from the turntable center to each camera's optical center. Utilising ROS ensured that all generated transforms and robot positions were consistently in metric units. Additionally, we established distinct kinematic chains for each arm to precisely align the plant's center with the middle of each captured image, an important factor for accurate 3D reconstruction.
We utilised two RealSense D435i cameras for image capture, mounted on the TCP of each UR5 robotic arm. The RealSense cameras were chosen for their capacity to capture high-quality RGB imaging, alongside precise depth mapping. These cameras allowed us acquire depth information that could be integrated into the 3D reconstruction process. The depth channel represents an optional addition to any 3D reconstruction pipeline, which we evaluate on page 8. We calculate the intrinsic parameters for each camera through a standard calibration process utilising a chessboard pattern and OpenCV's camera calibration [57] toolkit. These parameters can be combined with the camera pose, provided by the positioning of each robot, to produce a full mapping from 3D world coordinates into each image.
One of the key challenges associated with using a turntable is that, although the plant subject can rotate to any desired angle, the background remains consistent in each view. This is a challenge for view synthesis models, as the discrepancy between the foreground and background introduces significant noise during model training. We address this by using a white background, where the lack of notable features increased the quality of the final 3D reconstruction. We also experiment with additional background removal on page 8.
We observe that in some views the base of the second robot appears in the images captured from the first robot, adding additional noise in the final reconstructions and causing the plant to be rendered incorrectly. We resolved this issue by cropping each image to have

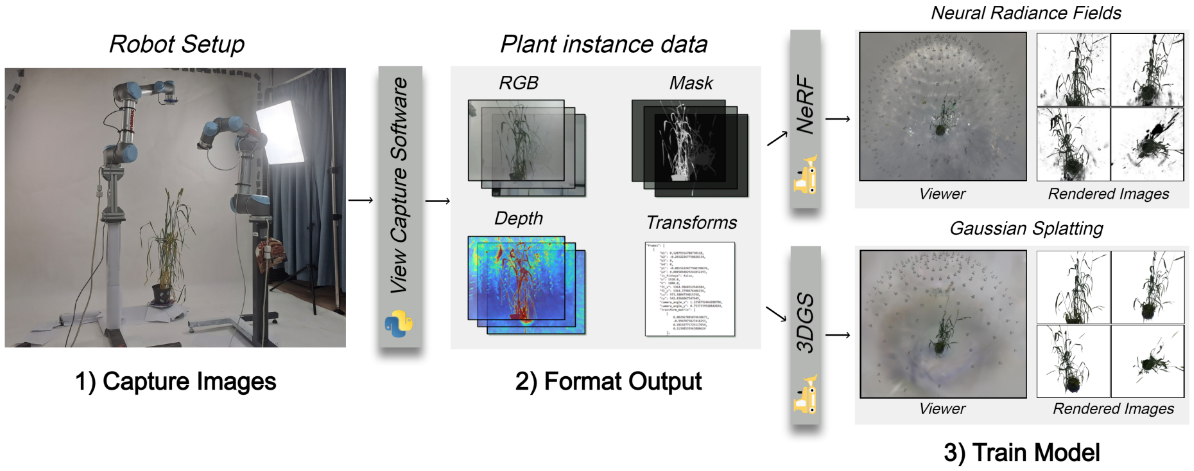

Figure 4. Overview of our entire process covering image capturing to 3D reconstruction. Firstly, a set of images and transforms are captured using the view capture framework. Next, the outputs are formatted. This includes operations such as refining transforms using bundle adjustment and generating masks. Finally, the generated dataset is trained using NeRFStudios' NeRF and 3DGS models.

a square aspect ratio with a pixel size of 1080x1080, improving reconstruction quality and reducing training time by half. We have found that increasing image resolution beyond this has no positive effect on reconstruction accuracy.

Two diffuse light sources were positioned either side of the plant to ensure that lighting would appear uniform, with a minimum of cast shadows and specular reflections, when the plant was 3D reconstructed.

Finally, apart from the turntable, we faithfully replicated our system in a Gazebo simulation environment. This allows the simulation to be run with an associated view capture software package to generate view synthesis datasets on synthetic 3D models. Our robot configuration files and comprehensive documentation can be accessed in our Git repository.

View Capturing Pipeline

We build upon our robotic platform and develop a highly customisable view capture framework capable of generating view synthesis datasets with any ROS-supported robot equipped with a camera and an associated MoveIt package. The framework is designed to capture image datasets with known transforms that can be used to train view synthesis models. This capture pipeline is shown in Figure 4.

In typical pipelines, SfM is required in order to determine the camera poses at each image. Our approach utilising robotics ensures that the camera poses are already known for each image, skipping the SfM stage that could lead to additional errors or inaccuracies. This also provides 3D coordinates in metric units, which is helpful for plant phenotyping problems, and forgoes the need to calibrate camera poses for each plant.

We have found that NeRF models in particular are sensitive to even very small inaccuracies in view position, such as errors in camera position of ~1mm. To address this issue, we incorporate bundle adjustment to refine our camera poses once capture is completed. The bundle adjustment process was initialised using the camera poses produced by our system, after which feature extraction, feature matching and point triangulation across the captured image set refines the position of each camera. This process was iterated three times to ensure accurate convergence. Once the camera poses had been optimised, each image was undistorted and a final point cloud was generated, since 3DGS models require this data to train correctly. This process was performed using COLMAP [58], a popular SfM framework.

A full capture run begins using an approximation of the dimensions of the plant, as well as the optimal radius around the object's center used for generating the camera positions. Exact dimensions of the plant are not necessary, during our experiments we typically chose a single value for all plants at the same growth stage. We found that capturing views roughly 1.5x the height of the plant resulted in the best reconstructions, since the majority of the plant is in the camera frame while not being too distant to impact image quality. Using a simple sphere point generation algorithm, potential views are formed around the object, with the turntable rotations being calculated as the angle between the fixed X axis and the generated points.

We then determined the closest robot to each specified point, adding that point into the respective robot's movement queue. During execution, each robot attempts to reposition to the next point in the queue in parallel. If this fails, each robot will then attempt to move to each point sequentially. At every point, an RGB-D image is captured alongside the current transform, which accurately maps the camera in 3D space relative to the current angle of the turntable. For each captured image an associated mask is generated that labels pixels that are part of the plant structure, which can be used for segmenting out the background. A simple pixel intensity threshold algorithm was used to remove pixels in the white background. Erosion and dilation functions are included to remove excess noise. Finally, a connected components process was utilised to identify the largest component in the mask, which we found was the plant in all cases, and other components are removed from the image. While both depth and segmentation information are useful inclusions to the dataset, these are not essential for training of view synthesis models. We experiment with including this data in the training process on page 8.

We have made all software, configuration files and documentation required to run our image capture pipeline publicly available.

Experiment

Data Acquisition

In order to evaluate the efficiency of view synthesis models for plant reconstructions, we captured a large dataset of wheat plant images and associated transforms. Wheat plants were chosen due to their agricultural significance and the challenges they present for 3D reconstruction, such as occluded leaves and thin stem structures.

To ensure that a diverse dataset was captured, 20 individual wheat plants, selected from 6 different genotypes (see below), were imaged at 6 distinct time points. Image capturing began 6 weeks after the plants were sown, and we captured these plants again at 7, 9, 10, 11, and 15 weeks. The plants were divided into three batches, with each batch being imaged on different days to maintain consistency. Each batch was transported from the University of Nottingham (UoN) Sutton Bonnington campus, where they were grown, to our imaging centre at the UoN Jubilee campus. On the 8th week, only 12 of the 20 plants were captured, due to a technical issue with one of the UR5s, delaying image capture for one week. We include this week regardless as 12 valid instances were captured. Plants were germinated in John Innes No.2 compost then vernalised at 6°C with 18 hours light, 6 hours dark for 4 weeks. After vernalisation, plants were potted into 2L pots with John Innes No.3 compost and grown in glasshouse conditions. The cultivars used in this study were provided by the UoN Wheat Research Centre and the John Innes Centre Germplasm Resource Unit, they are as follows: Chinese Spring, Langdon, BC1(1051-1054), GRU-2B(2J), GRU-2D(2J) and GRU-DA5J.

Chinese Spring is an elite cultivar of hexaploid bread wheat, *Triticum aestivum* (2n = 6x = 42 (AABBDD)). Langdon is an elite cultivar of tetraploid durum wheat, *Triticum turgidum* (2n = 4x = 28 (AABB)). BC1 plants are from an original cross of Chinese Spring x *Aegilops mutica* (2n = 2x = 14 (TT)), creating the first filial generation, and subsequently backcrossed to Chinese Spring creating a BC1 introgression line. GRU-2B(2J) and GRU-2D(2J) are *T. aestivum* with a chromosome substitution from *Thinopyrum bessarabicum* (2n = 2x = (JJ)) and GRU-DA5J are where a disomic additional chromosome of *Th. bessarabicum* is present.

These cultivars were selected based on their genetic variability. Bread wheat, durum wheat and wild relative substitution, additions and introgression lines all express varying phenotypes. This broad range of different wheat plant ensures that our dataset is extensive and provides additional challenges for downstream tasks.

To ensure consistent alignment, a crosshair icon was attached to the pot of each plant. This enabled us to position the plant in a similar pose and orientation for each capture session, potentially facilitating growth tracking over time and allowing simpler analysis between different 3D reconstructions of the same plant at different time steps.

During capture, approximately 320 RGB-D images were taken around each plant at equidistant intervals from the centre of the main stem. This number was chosen to balance reconstruction quality and capture time. A 1:8 ratio was used for our training/evaluation images, which ensured that our evaluation results correctly reflect the accuracy of the final reconstruction, while also ensuring that sufficient images were utilised in the training process. The entire imaging process, including post-capture bundle adjustment, took approximately 30 minutes for each plant.

On the 11th week, we captured a ground truth scan of each plant using an Einstar 3D Handheld Portable Scanner with an accuracy of 0.1 mm. This scan provided a precise ground truth 3D point cloud, allowing direct comparisons between the scans and model reconstructions.

Training

For each plant, we trained several variations of our captured data, as shown in Table 1. Our aim was to find the combination of image, transform and model that produced the best reconstruction results for both NeRF and 3DGS.

We trained using both original transforms and those refined via bundle adjustment. Next, models were trained using the standard RGB images with backgrounds, and others were trained using the segmented images with the background removed. Depth maps were also included when training the NeRF model; currently this is not supported in the 3DGS model. The 3DGS models were trained on undistorted images following bundle adjustment, as well as using the initial sparse point cloud produced by this process.

	Transforms Type	Training Images	Model
1	Original	RGB	NeRFacto
2	Refined	RGB	NeRFacto
3	Refined	Segmented RGB	NeRFacto
4	Refined	RGB+Depth Map	Depth-NeRFacto
5	Refined	Undistored	Splatfacto
6	Refined	Segmented Undistored	Splatfacto

Table 1. The different combinations of input images, transforms and models used for the various training setups.

Two variants of NeRF models were trained, NeRFacto and Depth-NeRFacto depending on whether an experiment utilised the depth information provided with each image. Each NeRF model was trained for 30,000 iterations, after which we observed no further improvement in performance. All models were trained using the Adam optimiser, and a batch size of 4096. We used an initial learning rate of 1×10^{-2} reducing to 1×10^{-4} over the training process. After training each model, the final iteration was used to evaluate testing performance. Each NeRF model was also converted into a point cloud and mesh using NeRFStudio.

For Gaussian splatting we utilised the Splatfacto model. Each was trained with a minimum alpha threshold of 5×10^{-3}, a scale threshold of 0.5mm, and a spherical harmonic degree of 3. Gaussians were initialised using the sparse point cloud generated using COLMAP during camera refinement process. We used the default learning rates for Splatfacto, which vary across the parameters such as mean, scale, orientation and spherical harmonic features. At the time of writing, no standard techniques were available to convert Gaussian splatting data into dense point clouds for analysis. Selecting only the centre positions of each Gaussian would produce a sparse point cloud. We therefore developed a new approach for this task.

Point clouds were generated by fixing the total number of points required, and distributing these appropriately across all Gaussian's in a scene based on their relative size. Thus, larger Gaussian generated more points. All points were sampled randomly from a Multivariate Normal distribution based on the 3D covariance matrix of each Gaussian. Point colours were derived by rendering images across the dataset for that scene, and tracking the contribution of each Gaussian to the final pixel colour at each camera location. Each Gaussian was coloured based on the pixel across the rendered images to which it contributed the most colour. This strategy prevents points with very high transparency being assigned erroneous colours that don't represent the final rendered scene. Our implementation produces accurate results and offers high customisation to support a variety of different scenes. This algorithm is also released alongside our other code.

All models took approximately 20 minutes to train using a single Nvidia Geforce RTX 2080 Ti graphics card. Alongside the trained models and exported point clouds, we also rendered a set of evaluation images to provide visual comparisons between the ground truth images and the trained models. These rendered images were used to generate the evaluation metrics for each plant. When rendering the evaluation images for the Splatfacto model, we added a near clip of 0.25m into the rendering pipeline, ensuring that Gaussians that are part of the background behind the camera did not occlude the plant. For NeRF, we set near and far ray clipping values of 0.01m and 5.0m respectively, avoiding reconstruction of spurious

points either very close or far from camera positions.
Each of these models can be executed via a Python script available in our dataset repository. This supports launching the models in NeRFStudio to view the reconstructed plants in 3D, as well as training new datasets on these models. A README file is also included that provides more information.

Results

Render Quality

We evaluate the effectiveness of each reconstruction approach using several metrics. Each metric compares the rendered evaluation image to the ground truth images, but focuses on highlighting different of types of inconsistencies between images.
NeRFStudio offers scripts that automatically generate the following metrics for the evaluation images:

i. **Peak Signal-to-Noise Ratio (PSNR)**: Measures the difference in the intensity of corresponding pixel values using the mean squared error formula. Higher PSNR values indicate lower distortion, with approximately values of 40db representing an image that is identical to the ground truth. PSNR values are logarithmic and thus represented using the decibel scale (db).
ii. **Structural Similarity Index (SSIM)**: Compares local patterns of pixel intensities normalised for factors such as luminance and contrast. Values range from -1 to 1, with 1 representing two identical images.
iii. **Learned Perceptual Image Patch Similarity (LPIPS)**: Calculates the perceptual similarities between two images by comparing the activations after passing through layers of a pre-trained convolution neural network (CNN). Lower values indicate higher perceptual similarities.

While these metrics are effective at measuring the similarity between the rendered image and ground truth, they consider the entire image, including the white background. This inclusion can overestimate the quality of the final render, where a simple white background represents a high proportion of the image, and is comparatively simple to render.
We introduce a PSNR masked metric to avoid this problem. This metric is based on the PSNR formula, but only includes pixels within the generated image mask. This approach provides a more accurate assessment of the effectiveness of the reconstruction on the plant itself. This metric can be considered alongside standard PSNR, which incorporates a measure of background quality.
The following section is split into a set of experiments, each examining the effectiveness of each of our trained model types. Each of these results are averaged over all 112 trained plant instances. Our goal is to identify the training data configuration that produces the best results for both NeRF and 3DGS.

The Effect of Bundle Adjustment on Camera Accuracy

First, we evaluate the impact of bundle adjustment on the accuracy of 3D reconstructions using RGB images. We compare the original transforms generated via our robot setup to those refined by the bundle adjustment process.

Training Type	PSNR ↑	SSIM ↑	LPIPS ↓	PSNR Masked ↑
Original (NeRF)	21.28	0.80	0.28	15.29
Refined (NeRF)	**23.93**	**0.86**	**0.22**	**19.47**

Table 2. Evaluation results for NeRF models trained on original transforms acquired from our setup, and transforms calculated using bundle adjustment. Both models were trained on RGB images.

Table 2 shows that, as expected, the bundle adjustment process improved the PSNR by approximately 2.5db. This shows the importance of extremely precise transform positions for these modern 3D reconstruction processes. As a result, we decided to utilise the refined transforms for all subsequent models, since they produce stronger results compared to the original transforms. We only conducted this comparison on NeRF models, as 3DGS models require the sparse point cloud initialisation after bundle adjustment.

The Impact of Depth on Synthetic View Quality

We examined the impact of including depth maps during model training. We performed these experiments using the NeRF models, as the 3DGS model does not currently support depth maps.

Training Type	PSNR ↑	SSIM ↑	LPIPS ↓	PSNR Masked ↑
RGB (NeRF)	23.93	0.86	**0.22**	**19.47**
RGB-D (NeRF)	**23.95**	**0.87**	**0.22**	18.15

Table 3. Evaluation results for NeRF models trained on RGB images, and RGB images with depth maps.

Perhaps counter intuitively, the inclusion of depth maps produced a slightly poorer final plant reconstruction, as highlighted by the PSNR Masked values in Table 3. The lower performance of RGB-D is caused by substandard render quality, particularly on thin individual leaf tips. It is likely that the depth maps were not sufficiently accurate to properly reconstruct the thin structures prevalent in plant shoots. Furthermore, the depth map resolution of 720x720 is lower than the RGB image resolution of 1080x1080, a typical restriction of RGB-D cameras. The additional advantage of using RGB images only is that this is more straightforward and cost efficient if deployed in future experiments based on our system. We therefore do not consider RGB-D for any further experiments.

The Effect of Background Removal on Synthetic View Quality

We experimented with background removal as a potential approach to more efficient reconstruction. NeRF and 3DGS models are trained to reconstruct the entire scene, which means that resource is spent reconstruction parts of the scene that are not relevant for plant reconstruction. Consequently, this can generate a white sphere around the plant during reconstruction, which can obstruct views outside the distance of the original captured images. We trained new models using the foreground masks described on page 6. The NeRF training process is adapted to produce no density or colour in areas of background. Similarly for 3DGS, the training processes is restricted to only generate Gaussians that appear in the masks when rendered, preventing reconstruction of the background.

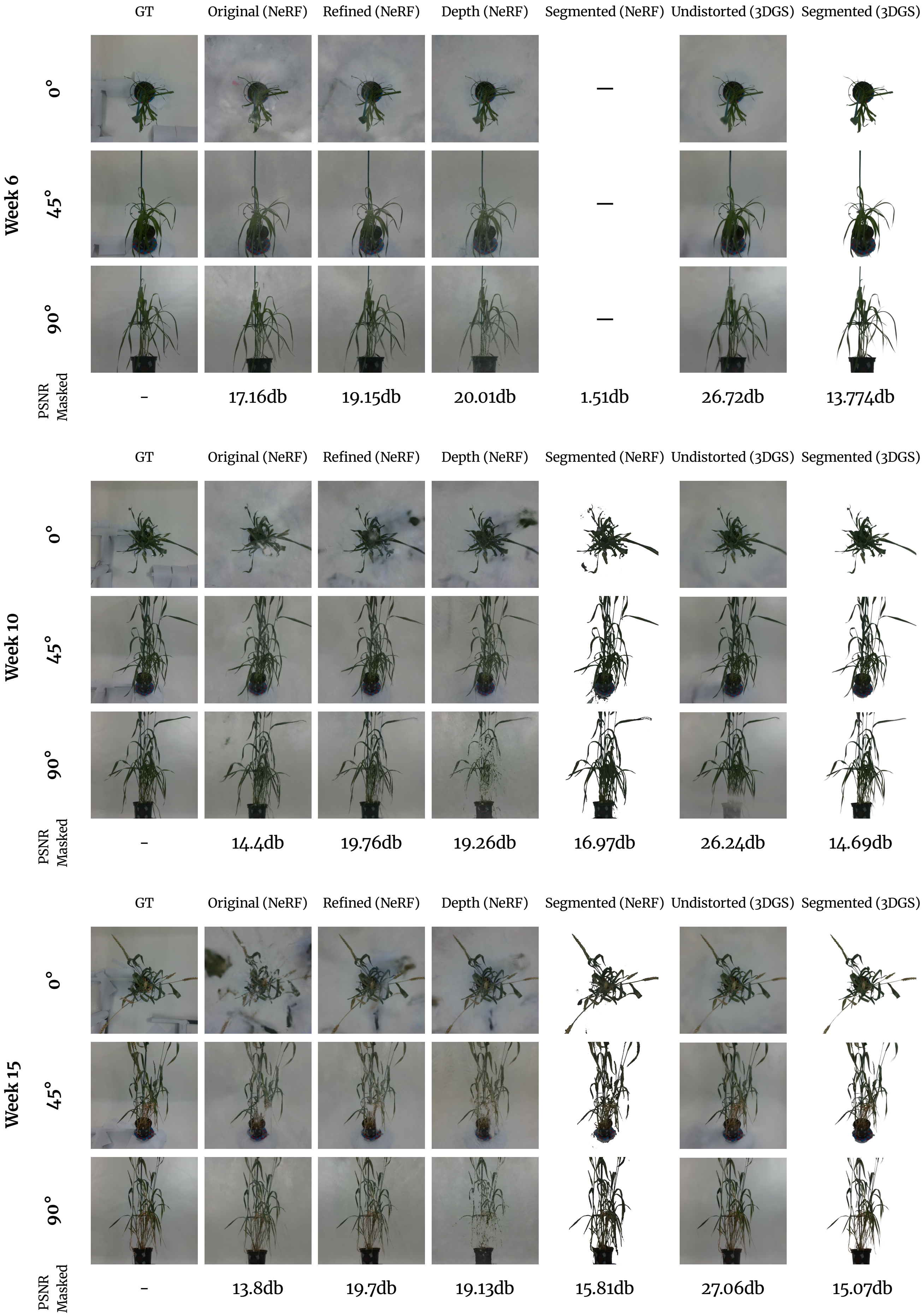

Figure 5. Visual comparison between the reconstruction results for plant bc1_1051 over three different time points. The left image is the ground truth, not included in the training images, and the images on the right are the rendered images for each of the different training configurations. Numerical values show the calculated PSNR masked metric for each evaluation image, with higher db values indicating better reconstruction quality. The NeRF model with segmented data did not train and produce a valid 3D reconstruction in week 6, which is why there are no rendered images. Also, the segmented NeRF models are automatically trained and rendered with a black background. We have altered all segmented NeRF images to have a white background by selecting all groups of black pixels and inverting them.

Training Type	PSNR ↑	SSIM ↑	LPIPS ↓	PSNR Masked ↑
RGB (NeRF)	**23.93**	**0.86**	**0.22**	**19.47**
Segmented (NeRF)	9.36	0.6	0.47	8.92
Undistorted (3DGS)	**28.17**	**0.95**	**0.15**	**26.31**
Segmented (3DGS)	21.10	0.94	0.10	13.75

Table 4. Evaluation results for NeRF and 3DGS models on both segmented and RGB images.

The results can be viewed in Table 4, and it can be seen that the segmented models perform less well than using standard RGB images for NeRFs and 3DGS. These techniques are already reliant on highly accurate camera positions, the addition of potentially imperfect segmentation can compound any loss in accuracy. We experimented with a variety of foreground segmentation techniques, including convolutional networks. However we found none offered sufficiently high accuracy, and all introduce an additional unnecessary process within the reconstruction pipeline.

A Comparison of Robot-derived and SfM Calculated Camera Poses

To compare our image capture setup to standard SfM, we trained the models on transforms generated entirely using COLMAP's SfM functionality, which is a common approach to calibration and reconstruction across image datasets with unknown camera poses.

Training Type	PSNR ↑	SSIM ↑	LPIPS ↓	PSNR Masked ↑
Ours (NeRF)	**23.93**	**0.86**	**0.22**	**19.47**
SfM (NeRF)	22.021	0.822	0.297	16.924
Ours (3DGS)	**28.17**	**0.95**	**0.15**	**26.31**
SfM (3DGS)	27.70	0.942	0.162	25.643

Table 5. Evaluation results for NeRF and 3DGS models. One set was trained using our transforms acquired from the robot setup and bundle adjustment. Another was trained using transforms acquired from SfM. Both were trained using RGB images.

As seen in Table 5, our pipeline shows higher accuracy over a traditional SfM approach. SfM uses a similar bundle adjustment approach to our refinement step, but must start this process from scratch, rather than from already accurate robot position data. The SfM process therefore often failed to calculate the positions for all images, only identifying camera positions for an average of 265/320 images per plant scene. The failed images could not be used within any downstream reconstruction process.

Synthetic View Quality of NeRF and 3DGS

Finally, we compared the performance of the 3DGS model to the NeRF model for rendering new synthetic views of each plant. The results in Table 6 show the 3DGS model produced higher quality synthetic views compared to the NeRF model. From visual observations, there was reduced noise in the 3DGS reconstruction, particularly with view points above the plant. We hypothesise that this is due to 3DGS being more effective at resolving inconsistent background appearance in top-down views, where the robot pedestals are visible. It also seemed that the NeRF models struggled more when handling thin structures on the plant, while the 3DGS models appear to reconstruct these features more effectively.

Training Type	PSNR ↑	SSIM ↑	LPIPS ↓	PSNR Masked ↑
NeRF	23.93	0.86	0.22	19.47
3DGS	**28.17**	**0.95**	**0.15**	**26.31**

Table 6. Evaluation results for NeRF and 3DGS models. Both were trained on the original RGB images and transforms calculated using bundle adjustment.

Reconstruction Accuracy

Whilst rendering new images of the captured plant is useful, the accuracy of the final plant reconstruction is crucial for extraction of correct phenotypic traits. To measure the accuracy of the NeRF and 3DGS reconstructions, we generated point clouds from the different models and compared these to our captured ground truths. We utilised CloudCompare, an open-source project designed for handling 3D point clouds, to calculate a final accuracy metric [59]. We use average point distance to perform this comparison. This is calculated as the average distance of each point on the reconstructed point cloud, to the nearest point on the ground truth.

3D Reconstruction Method	Mean Distance (mm)	SD (mm)
NeRF	1.89	2.43
3DGS	1.91	3.86
Structure from Motion (SfM)	11.35	9.05

Table 7. The accuracy of each of the 3D reconstruction methods when compared to the captured ground truth scans.

We first generated a point cloud representation for the final NeRF and 3DGS models. It is important to note that, since both NeRF and 3DGS are dense data structures, there is no limit to the number of points that can be generated by each representation. We chose to generate exactly 10,000,000 points, which ensured our point clouds were dense enough for an accurate comparison against the ground truth. We cropped the generated point cloud using an axis-aligned bounding box to ensure that the background was not included in the point generation process. We set the bounding box size to 1mx1mx2.5m and set the centre of this box to the origin of the scene. This ensured that the entire plant was reconstructed, with little of the background being included.
For 3DGS point clouds, we set specific parameters during point generation to ensure that points best fit the reconstructed Gaussian. Firstly, points that have a Mahalanobis distance greater than 2.5 standard deviation (SD) from their Gaussian centre were removed and regenerated. Gaussians with an opacity less than 1% were culled and Gaussians with a volume in the top 2.5% of all Gaussian sizes were removed, since we observed these Gaussians were always part of the background.
Each reconstruction was cleaned using a set of common automatic operations. Firstly, a statistical outlier removal algorithm was implemented that groups neighbouring points together, and then any point that lies a distance further than 1 SD from the local group is removed. Next, a noise filter was used that fit an approximate surface across all points, and removed points further than 1 SD from the predicted surface. Finally, points were clustered, and groups of points with fewer than 2000 connected points were rejected. These operations can be automated, such that the majority of points that were part of the true plant reconstruction were included in generating our accuracy metrics. Each point

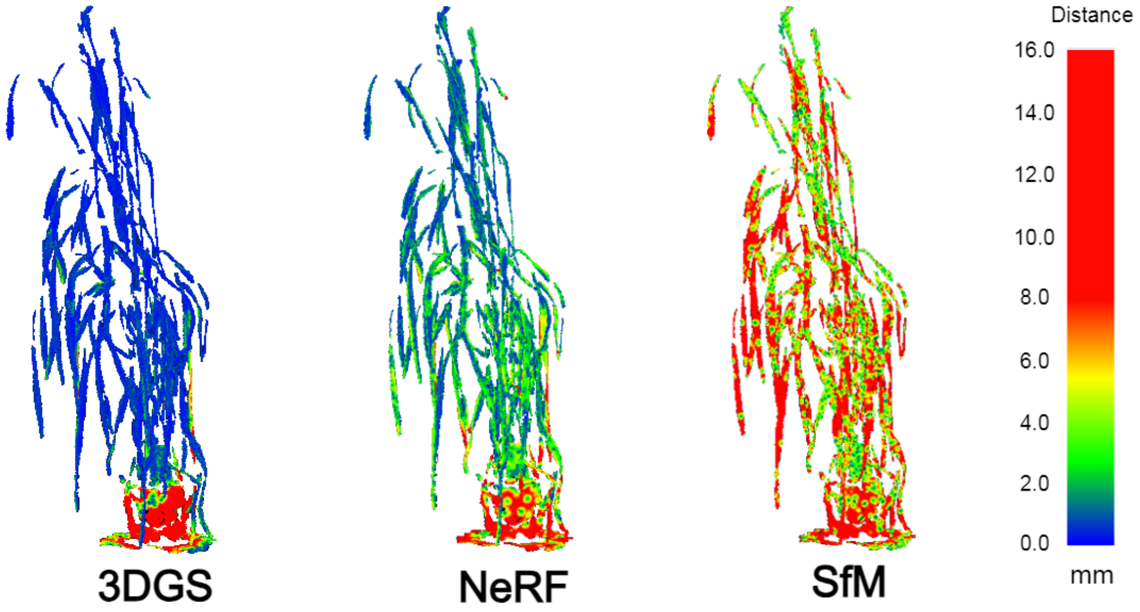

Figure 6. Comparison between the distance error for the NeRF, 3DGS and SfM produced point clouds for the langdon_3 plant on week 11. On the right is the colourmap key, with blue indicating a low distance error, and red indicating a high distance error.

cloud had approximately 7,500,000 points after performing these operations.

Next, we registered and aligned each reconstruction point cloud with the scanned ground truth point cloud via the Iterative Closest Point algorithm. Since the camera positions were captured using our robot setup, all the reconstructed point clouds are in metric units, allowing for direct comparison in millimeters without any scaling. We then compare the average point distance to the ground truth, as described above, with results presented in Table 7.

Both 3DGS and NeRF models produced very similar accuracy over all plant reconstructions. However, we note that point clouds generated by 3DGS had higher precision around thin structures of the plant, such as the stems, most likely to the dense population of Gaussians in these areas. NeRF typically produced point clouds with reduced noise, as shown by the lower standard deviation, and these point clouds may better represent larger surfaces. This is visualised in Figure 6, where 3DGS had a higher accuracy around the thin stems of the plant, but struggled with larger areas, such as the pot. Both approaches are suitable candidates for 3D reconstruction, offering similar effective representations. We discuss the benefits and drawbacks of each in the following section.

To compare the accuracy against other reconstruction techniques we also generated a sparse point cloud using SfM. We utilised COLMAP to generate a point cloud using the camera poses calculated after bundle adjustment.

Overall, both NeRF and 3DGS reconstructions were more accurate than the SfM reconstructions. View synthesis models are more effective at representing detailed structures on the plants, which includes complex leaf shape, topology, and self-occlusion. Visualised results may be found in Figure 6. NeRFs and 3DGS can reconstruct scenes as they appear in each camera view, including areas of low texture. SfM, in contrast, must extract, match and triangulate each point between images accurately, which may lead to additional noise on narrow plant structures that have low contrast and texture.

Discussion

Reconstruction of plant shoots in 3D has remained a substantial challenge for many years. We have shown here that both NeRF and 3DGS exhibit remarkable capabilities in reconstructing plants with diverse physical characteristics and complex topology. These approaches rival traditional standardised 3D reconstruction techniques, and often provide higher accuracy over common approaches such as SfM. When used for view synthesis, these models can provide new high-quality synthetic images of plants from views that have not been captured in the original dataset, potentially driving new research in active phenotyping using robotic manipulators, and improving our ability to capture phenotypic traits in the presence of substantial occlusion.

NeRF and 3DGS models offer two different approaches, that while superficially similar, are quite different. NeRF models train a neural network to generate an implicit scene representation, where ray-marching is then used to sample colour and density from this space. This approach has some notable advantages: models are continuous representations, allowing us to sample higher resolution images by simply casting more rays into the scene, at the cost of longer render times. The neural models predict not only colour, but the opacity of material in 3D space, allowing them to be easily converted into volumetric representations such as voxel grids, or 3D representations such as meshes. Utilising a neural network means that an entire scene, regardless of the number of images or scale, can be encoded with a consistent file size of roughly 200MB for a NeRFacto model. These allow NeRF to be used as part of phenotyping pipelines that leverage these representations, with potentially higher accuracy than previous reconstruction methods.

3DGS instead represents the scene as a series of 3D coloured ellipsoids. This representation is closer to a traditional point cloud representation, but where each point has additional parameters governing shape and colour. Our results in Table 6 show that 3DGS is capable of extremely high quality view synthesis, often outperforming NeRF on this task. The 3D Gaussians can be converted into traditional point cloud representations through careful sampling, from which common phenotyping pipelines can be run. Since the representation is held as discrete points, noise and background removal is typically straightforward, for example by simply removing points outside of a predictable range. However, the number of Gaussians needed to reconstruct a scene can vary depending on the complexity of the training data, meaning that complex scenes can have a file size larger than 1GB. Despite this, 3DGS offers efficient rasterisation, meaning that new views can be generated almost

instantly, and comfortably at >60fps on a modern desktop PC. This is compared with a NeRF model, where volumetric ray marching can take approximately 5 seconds per image to render.
The training times for both NeRF and 3DGS are comparable, with each plant instance requiring approximately 20 minutes. Variants of NeRF exist that reduce the time required to train [37] but these often reduce image quality, and we have focused here on the maximum quality possible as a demonstration of the technology. Both NeRF and 3DGS are active areas of research, and it is likely that some limitations of these approaches will be addressed over the coming years. Our plant dataset provides a new test environment in which to evaluate new developments in these approaches, and improving NeRF and 3DGS for plants specifically represents a promising area for future work.

Automated Dataset Capture

A notable challenge of both approaches is their requirement for highly accurate camera positions. As shown in Table 2, slight errors in parameters can lead to lower quality reconstructions. These can be obtained using a pipeline such as ours, combined with modern bundle adjustment algorithms, but we foresee these pipelines becoming a requirement for successful phenotyping using these state-of-the-art approaches. Our robotic image capture system and framework offer several advantages over static or limited capture setups. Firstly, our system captures high quality images around plants of various different sizes. By utilising robot path planning, dynamic generation of positions allows for flexible image capture should requirements change. This framework is highly customisable, ensuring repeatability across a variety of bespoke ROS setups. Unlike unconstrained image capture setups, for example using a hand-held camera, our system is calibrated such that even after refinement using bundle adjustment, all camera positions and reconstructions are represented in metric units. This is a feature not commonly found in other view synthesis datasets, and the use of ROS compatible hardware allows other researchers to utilise this setup.
Capturing high quality data on living organisms such as plants remains a challenge. Transporting each plant from the greenhouse to the imaging setup occasionally resulted in damage, particularly to the spikes. With the larger wheat plants, stems occasionally became entangled with the stand of the second robot, causing discrepancies between views, resulting in floating artifacts in the reconstructions. These issues are shown in Figure 7. We anticipate that the most effective solutions will be based within the growth environments themselves, and adapting our system to in situ robotics is an area of potential future research.

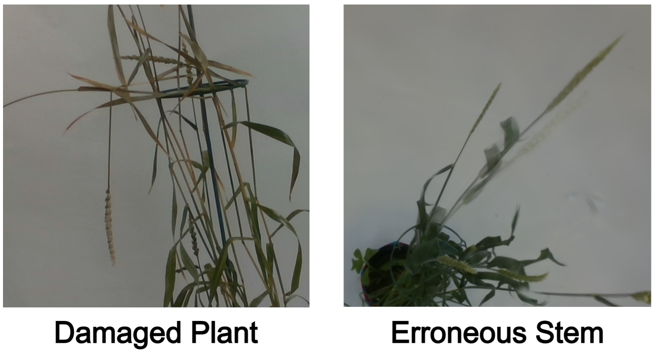

Figure 7. Examples of some of the issues with our dataset. Left shows an image of a damaged stem on plant bc1_1053_1 (16-04-24). Right image shows an image of an erroneous stem being rendered using 3DGS for plant bc1_1033_1 (06-03-24). This is due to a collision that stem had with the robot 2 stand, that caused it to move incorrectly during capturing.

We found that segmenting plants prior to reconstructions did not consistently improve results, and often led to poor reconstructions due to inaccuracies and inconsistencies between masks at different views. When rendering images outside of the capture radius, we noted that areas of background might be rendered instead of the plant. This caused particular issues when rendering the 3DGS images, as large Gaussians representing the white background may obscure the plant in some views. We found that post-processes to remove these Gaussians were more effective than adapting the image capture or 3DGS training process.

Conclusion

We have presented a new dataset for multi-view reconstruction of plant shoots. By utilising a dual-robot image capture system and a turntable, we capture full 360 degree views of each plant, adapted to their size. This capture setup produces accurate camera positions in metric units, with associated high resolution images, and depth information. Using this dataset we demonstrate the strong performance of two recent approaches to view synthesis, Neural Radiance Fields and 3D Gaussian Splatting. We demonstrate state-of-the-art performance in both view synthesis and 3D model reconstruction. On our test data captured using a handheld scanner, the trained NeRF and 3DGS models had an average surface accuracy of 1.89mm and 1.91mm respectively, compared to 11.35mm for a recent and popular SfM technique. We argue that both approaches will lead to a step-change in our ability to capture 3D models of plants, which have historically proved very challenging due to their complex shape, frequent occlusion and self-similarity. We release all configuration files and scripts associated with our image capture system, which can be deployed on any ROS compatible hardware. We also release our dataset of 112 wheat plants captured approximately ~300 times each, and associated camera position in metric units. Finally, we release all training scripts and trained NeRF and 3DGS models, and 3D reconstruction output across all plants. We hope that our paper will provide opportunities for researchers exploring new and improved 3D phenotyping algorithms, 3D reconstruction and view synthesis research, and active vision systems.

Availability of Source Code and Requirements

Project name: 3D Plant View Synthesis
Project home page:
`https://github.com/Lewis-Stuart-11/3D-Plant-View-Synthesis`
Operating system(s): Windows, Ubuntu
Programming language: Python (>=3.8)
License: Apache 2.0
Any restrictions to use by non-academics: None

Functionality, such as Robotic View Capturing, 3DGS to Point Cloud and our UR5 Configs files, are stored on separate GitHub repositories that can be accessed via the project README.

Availability of Supporting Data and Materials

Snapshots of our main dataset and data further supporting this work are openly available in the GigaScience repository, GigaDB **[Temporary Review URL].**

Abbreviations

3DGS: 3D Gaussian Splatting; CNN: Convolutional Neural Network; LiDAR: Light Detection and Ranging; LPIPS: Learned Perceptual Image Patch Similarity; MVS: Multi-View Stereo; NeRF: Neural

Radiance Field; PSNR: Peak Signal-to-Noise Ratio; RGB: Red, Green and Blue; ROS: Robot Operating System; SD: Standard Deviation; SfM: Structure From Motion; SSIM: Structural similarity index measure; TCP: Tool Center Point; UoN: University of Nottingham; URDF: Unified Robot Description Format;

Competing Interests

The Authors declare that they have no competing interests.

Funding

This work was supported by the Doctoral Training Centre in Artificial Intelligence, an UoN studentship for multidisciplinary research focused on AI.
Furthermore, the International Agriculture Doctoral Training Programme, an UoN studentship scheme started by the Future Food Beacon of Excellence, assisted the maintenance of the wheat plant used in our experiments.

Authors' Contributions

M.P.P, D.M.W and J.A.A designed the project; L.A.G.S implemented the view capturing software, generated the complete dataset and handling duel robot configuration/calibration, under the direct supervision of M.P.P; D.M.W and J.A.A assisted in implementing the turntable, robot stands and organising wheat plant capturing; S.C configured the initial robot setup and provided resources for extension; J.W handled plant selection, germination and maintenance while in the greenhouse. L.A.G.S and M.P.P wrote the manuscript. All authors contributed to and approved the final manuscript.

Acknowledgements

We thank Alexis J S Payne and Dr Michael Wilson of the University of Nottingham for their contributions in the development of the Docker file used for running NeRFStudio with our dataset.

References

1. Harandi N, Vandenberghe B, Vankerschaver J, Depuydt S, Van Messem A. How to make sense of 3D representations for plant phenotyping: a compendium of processing and analysis techniques. Plant Methods 2023 Jun;19(1):60. https://doi.org/10.1186/s13007-023-01031-z.
2. Zhang H, Wang L, Jin X, Bian L, Ge Y. High-throughput phenotyping of plant leaf morphological, physiological, and biochemical traits on multiple scales using optical sensing. The Crop Journal 2023;11(5):1303–1318. https://www.sciencedirect.com/science/article/pii/S2214514123000740.
3. Jin S, Sun X, Wu F, Su Y, Li Y, Song S, et al. Lidar sheds new light on plant phenomics for plant breeding and management: Recent advances and future prospects. ISPRS Journal of Photogrammetry and Remote Sensing 2021;171:202–223. https://www.sciencedirect.com/science/article/pii/S0924271620303130.
4. Jay S, Rabatel G, Hadoux X, Moura D, Gorretta N. In-field crop row phenotyping from 3D modeling performed using Structure from Motion. Computers and Electronics in Agriculture 2015;110:70–77. https://www.sciencedirect.com/science/article/pii/S0168169914002609.
5. Mildenhall B, Srinivasan PP, Tancik M, Barron JT, Ramamoorthi R, Ng R, NeRF: Representing Scenes as Neural Radiance Fields for View Synthesis; 2020.
6. Kerbl B, Kopanas G, Leimkühler T, Drettakis G. 3d gaussian splatting for real-time radiance field rendering. ACM Transactions on Graphics (ToG) 2023;42(4):1–14.
7. Reynolds M, Foulkes J, Furbank R, Griffiths S, King J, Murchie E, et al. Achieving yield gains in wheat. Plant Cell Environ 2012 Aug;35(10):1799–1823.
8. Ray DK, Mueller ND, West PC, Foley JA. Yield Trends Are Insufficient to Double Global Crop Production by 2050. PLOS ONE 2013 06;8:1–8. https://doi.org/10.1371/journal.pone.0066428.
9. Stanford Artificial Intelligence Laboratory et al , Robotic Operating System;. https://www.ros.org.
10. Koenig N, Howard A. Design and use paradigms for Gazebo, an open-source multi-robot simulator. In: 2004 IEEE/RSJ International Conference on Intelligent Robots and Systems (IROS) (IEEE Cat. No.04CH37566), vol. 3; 2004. p. 2149–2154 vol.3.
11. Elnashef B, Filin S, Lati RN. Tensor-based classification and segmentation of three-dimensional point clouds for organ-level plant phenotyping and growth analysis. Computers and electronics in agriculture 2019;156:51–61.
12. Lou L, Liu Y, Shen M, Han J, Corke F, Doonan JH. Estimation of Branch Angle from 3D Point Cloud of Plants. In: 2015 International Conference on 3D Vision; 2015. p. 554–561.
13. Ziamtsov I, Navlakha S. Machine learning approaches to improve three basic plant phenotyping tasks using three-dimensional point clouds. Plant physiology 2019;181(4):1425–1440.
14. Feldman A, Wang H, Fukano Y, Kato Y, Ninomiya S, Guo W. EasyDCP: An affordable, high-throughput tool to measure plant phenotypic traits in 3D. Methods in Ecology and Evolution 2021;12(9):1679–1686.
15. Schunck D, Magistri F, Rosu RA, Cornelißen A, Chebrolu N, Paulus S, et al. Pheno4D: A spatio-temporal dataset of maize and tomato plant point clouds for phenotyping and advanced plant analysis. PLOS ONE 2021 08;16(8):1–18. https://doi.org/10.1371/journal.pone.0256340.
16. Das Choudhury S, Maturu S, Samal A, Stoerger V, Awada T. Leveraging image analysis to compute 3D plant phenotypes based on voxel-grid plant reconstruction. Frontiers in Plant Science 2020;11:521431.
17. Tross MC, Gaillard M, Zwiener M, Miao C, Grove RJ, Li B, et al. 3D reconstruction identifies loci linked to variation in angle of individual sorghum leaves. PeerJ 2021;9:e12628.
18. Paproki A, Sirault X, Berry S, Furbank R, Fripp J. A novel mesh processing based technique for 3D plant analysis. BMC plant biology 2012;12:1–13.
19. Okura F. 3D modeling and reconstruction of plants and trees: A cross-cutting review across computer graphics, vision, and plant phenotyping. Breeding Science 2022;72(1):31–47.
20. Paulus S, Schumann H, Kuhlmann H, Léon J. High-precision laser scanning system for capturing 3D plant architecture and analysing growth of cereal plants. Biosystems Engineering 2014;121:1–11. https://www.sciencedirect.com/science/article/pii/S1537511014000166.
21. Gibbs JA, Pound M, French AP, Wells DM, Murchie E, Pridmore T. Approaches to three-dimensional reconstruction of plant shoot topology and geometry. Functional Plant Biology 2016;44(1):62–75.
22. Forero MG, Murcia HF, Méndez D, Betancourt-Lozano J. LiDAR Platform for Acquisition of 3D Plant Phenotyping Database. Plants 2022;11(17). https://www.mdpi.com/2223-7747/11/17/2199.
23. Paulus S, Behmann J, Mahlein AK, Plümer L, Kuhlmann H. Low-Cost 3D Systems: Suitable Tools for Plant Phenotyping. Sensors 2014;14(2):3001–3018. https://www.mdpi.com/1424-8220/14/2/3001.
24. McCormick RF, Truong SK, Mullet JE. 3D sorghum reconstructions from depth images identify QTL regulating shoot architecture. Plant physiology 2016;172(2):823–834.

25. Li D, Xu L, Tang Xs, Sun S, Cai X, Zhang P. 3D Imaging of Greenhouse Plants with an Inexpensive Binocular Stereo Vision System. Remote Sensing 2017;9(5). https://www.mdpi.com/2072-4292/9/5/508.
26. Hui F, Zhu J, Hu P, Meng L, Zhu B, Guo Y, et al. Image-based dynamic quantification and high-accuracy 3D evaluation of canopy structure of plant populations. Annals of Botany 2018 03;121(5):1079–1088. https://doi.org/10.1093/aob/mcy016.
27. Li D, Shi G, Kong W, Wang S, Chen Y. A leaf segmentation and phenotypic feature extraction framework for multiview stereo plant point clouds. IEEE Journal of Selected Topics in Applied Earth Observations and Remote Sensing 2020;13:2321–2336.
28. Pound MP, French AP, Murchie EH, Pridmore TP. Automated recovery of three-dimensional models of plant shoots from multiple color images. Plant physiology 2014;166(4):1688–1698.
29. Wu S, Wen W, Gou W, Lu X, Zhang W, Zheng C, et al. A miniaturized phenotyping platform for individual plants using multi-view stereo 3D reconstruction. Frontiers in plant science 2022;13:897746.
30. Paturkar A, Sen Gupta G, Bailey D. Non-destructive and cost-effective 3D plant growth monitoring system in outdoor conditions. Multimedia Tools and Applications 2020 12;79.
31. Iglhaut J, Cabo C, Puliti S, Piermattei L, O'Connor J, Rosette J. Structure from motion photogrammetry in forestry: A review. Current Forestry Reports 2019;5:155–168.
32. Wang Y, Wen W, Wu S, Wang C, Yu Z, Guo X, et al. Maize Plant Phenotyping: Comparing 3D Laser Scanning, Multi-View Stereo Reconstruction, and 3D Digitizing Estimates. Remote Sensing 2019;11(1). https://www.mdpi.com/2072-4292/11/1/63.
33. Kochi N, Isobe S, Hayashi A, Kodama K, Tanabata T. Introduction of All-Around 3D Modeling Methods for Investigation of Plants. International Journal of Automation Technology 2021 05;15:301–312.
34. Paulus S. Measuring crops in 3D: using geometry for plant phenotyping. Plant Methods 2019 Sep;15(1):103. https://doi.org/10.1186/s13007-019-0490-0.
35. Barron JT, Mildenhall B, Tancik M, Hedman P, Martin-Brualla R, Srinivasan PP. Mip-NeRF: A Multiscale Representation for Anti-Aliasing Neural Radiance Fields. CoRR 2021;abs/2103.13415. https://arxiv.org/abs/2103.13415.
36. Barron JT, Mildenhall B, Verbin D, Srinivasan PP, Hedman P. Mip-NeRF 360: Unbounded Anti-Aliased Neural Radiance Fields. CoRR 2021;abs/2111.12077. https://arxiv.org/abs/2111.12077.
37. Müller T, Evans A, Schied C, Keller A. Instant Neural Graphics Primitives with a Multiresolution Hash Encoding. ACM Trans Graph 2022 Jul;41(4):102:1–102:15. https://doi.org/10.1145/3528223.3530127.
38. Tancik M, Weber E, Ng E, Li R, Yi B, Kerr J, et al. Nerfstudio: A Modular Framework for Neural Radiance Field Development. In: ACM SIGGRAPH 2023 Conference Proceedings SIGGRAPH '23; 2023. .
39. Jignasu A, Herron E, Jubery TZ, Afful J, Balu A, Ganapathysubramanian B, et al. Plant Geometry Reconstruction From Field Data Using Neural Radiance Fields. In: 2nd AAAI Workshop on AI for Agriculture and Food Systems; 2023. https://openreview.net/forum?id=TvKKqWn_-6.
40. Arshad MA, Jubery T, Afful J, Jignasu A, Balu A, Ganapathysubramanian B, et al., Evaluating NeRFs for 3D Plant Geometry Reconstruction in Field Conditions; 2024.
41. Hu K, Ying W, Pan Y, Kang H, Chen C. High-fidelity 3D reconstruction of plants using Neural Radiance Fields. Computers and Electronics in Agriculture 2024 May;220:108848. http://dx.doi.org/10.1016/j.compag.2024.108848.
42. Saeed F, Sun J, Ozias-Akins P, Chu YJ, Li CC. PeanutNeRF: 3D Radiance Field for Peanuts. In: Proceedings of the IEEE/CVF Conference on Computer Vision and Pattern Recognition; 2023. p. 6253–6262.
43. Zhao J, Ying W, Pan Y, Yi Z, Chen C, Hu K, et al., Exploring Accurate 3D Phenotyping in Greenhouse through Neural Radiance Fields; 2024.
44. Lu T, Yu M, Xu L, Xiangli Y, Wang L, Lin D, et al., Scaffold-GS: Structured 3D Gaussians for View-Adaptive Rendering; 2023.
45. Fan Z, Wang K, Wen K, Zhu Z, Xu D, Wang Z, LightGaussian: Unbounded 3D Gaussian Compression with 15x Reduction and 200+ FPS; 2024.
46. Chaudhury A, Ward C, Talasaz A, Ivanov AG, Brophy M, Grodzinski B, et al. Machine vision system for 3D plant phenotyping. IEEE/ACM transactions on computational biology and bioinformatics 2018;16(6):2009–2022.
47. Wu S, Wen W, Wang Y, Fan J, Wang C, Gou W, et al. MVS-Pheno: a portable and low-cost phenotyping platform for maize shoots using multiview stereo 3D reconstruction. Plant Phenomics 2020;.
48. Nguyen TT, Slaughter DC, Max N, Maloof JN, Sinha N. Structured Light-Based 3D Reconstruction System for Plants. Sensors 2015;15(8):18587–18612. https://www.mdpi.com/1424-8220/15/8/18587.
49. Gao T, Zhu F, Paul P, Sandhu J, Doku HA, Sun J, et al. Novel 3D imaging systems for high-throughput phenotyping of plants. Remote Sensing 2021;13(11):2113.
50. Liu S, Acosta-Gamboa LM, Huang X, Lorence A. Novel Low Cost 3D Surface Model Reconstruction System for Plant Phenotyping. Journal of Imaging 2017;3(3). https://www.mdpi.com/2313-433X/3/3/39.
51. Kochi N, Hayashi A, Shinohara Y, Tanabata T, Kodama K, Isobe S. All-around 3D plant modeling system using multiple images and its composition. Breeding science 2022;72(1):75–84.
52. Bao Y, Zarecor S, Shah D, Tuel T, Campbell D, Chapman A, et al. Assessing plant performance in the Enviratron. Plant Methods 2019 10;15.
53. Atefi A, Ge Y, Pitla S, Schnable J. Robotic Detection and Grasp of Maize and Sorghum: Stem Measurement with Contact. Robotics 2020;9(3). https://www.mdpi.com/2218-6581/9/3/58.
54. Zahid A, Mahmud MS, He L, Heinemann P, Choi D, Schupp J. Technological advancements towards developing a robotic pruner for apple trees: A review. Computers and Electronics in Agriculture 2021;189:106383. https://www.sciencedirect.com/science/article/pii/S0168169921004002.
55. Wu C, Zeng R, Pan J, Wang CC, Liu YJ. Plant phenotyping by deep-learning-based planner for multi-robots. IEEE Robotics and Automation Letters 2019;4(4):3113–3120.
56. David C, Ioan S A, Sachin C, Nikolaus C, Reducing the Barrier to Entry of Complex Robotic Software: a MoveIt! Case Study; 2014.
57. Itseez, Open Source Computer Vision Library; 2015. https://github.com/itseez/opencv.
58. Schönberger JL, Frahm JM. Structure-from-Motion Revisited. In: Conference on Computer Vision and Pattern Recognition (CVPR); 2016. .
59. Girardeau-Montaut D, et al. CloudCompare. France: EDF R&D Telecom ParisTech 2016;11(5).
