# Supplementary material for: High-fidelity wheat plant reconstruction using 3D Gaussian splatting and neural radiance fields
Source: Gigascience. 2025 Mar 26;14:giaf022. doi: 10.1093/gigascience/giaf022 (PMC11945317; doi:10.1093/gigascience/giaf022)
Supplement: giaf022_Response_to_Reviewer_Comments_Original_Submission [file giaf022_response_to_reviewer_comments_original_submission.pdf]

Author's Response To Reviewer Comments

We thank both reviewers for their detailed and constructive feedback. We believe that the reviewers' suggestions have substantially strengthened our submission. We have addressed each point in turn below, and also provide a highlighted copy to facilitate any further reviews.

Reviewer 1

1. "The research used both 3DGS and NeRF to verify the effectiveness of the method. Generally speaking, 3DGS has better reconstruction efficiency than NeRF. Why doesn't the author put effort into improving the 3DGS method to achieve better results in reconstruction? Only through the comparison of two ready-made methods, it is insufficient to reflect the progressiveness of the research. Suggest the author to conduct more methodological research to achieve convincing results."

While our results show that 3DGS produces slightly improved results over NeRF on our dataset, we believe it is still valid to present results on NeRF in the paper. NeRF is still an active research area, and as such it remains possible that competitive methods will appear. There may also be scenarios where NeRF is more suitable than 3DGS, for example in problems requiring implicit / continuous scene representations, such as ray casting for light dynamics, or methods that seek to generate a truncated signed distance function representing plant models.
The primary goal of this paper is to showcase our dataset, and compare currently top performing NeRF and 3DGS models to existing 3D reconstruction techniques such as MVS. We hope that the dataset will drive forward innovation in plant reconstruction, but we feel that substantial alterations or improvements to either NeRF or 3DGS are out of scope for this manuscript. This remains a worthwhile area for future research, and we have highlighted this in the discussion section.

2. "3D Gaussian Splatting is an explicit representation method that utilizes a set of Gaussian ellipsoids to model the scene, thereby achieving fast new view synthesis rendering speed. This article lacks effective analysis on the speed of various methods such as SMF, 3DGS, NERF, etc. I hope it can be supplemented."

We agree with the reviewer's comment. We have added a new section to address this titled 'Comparison of View Synthesis Models and Traditional 3D Reconstruction Techniques', which discusses the training times and rendering times highlighted in Table 8. Here we evaluate the average training times for each of the models on the RGB images for each our plant instances. We show that SfM is fastest, but will produce a lower quality reconstruction. 3DGS and NeRF can be trained in 15 minutes and 20 minutes respectively. Finally, MVS required over 2 hours to reconstruct a scene.
We also evaluate the speed of rendering new high-quality images for both 3DGS and NeRF. We find that 3DGS offers faster rendering than NeRF, with new 2K images rendered at ~15 FPS, with NeRF taking approximately 5 seconds for an image of the same size.

3. "The characteristics of 3D Gaussian Splatting facilitate editing tasks such as dynamic reconstruction, geometric editing, and physical simulation. Further experiments and comparative analysis are needed to improve rendering efficiency and solve discontinuous shading problems by introducing Gaussian ellipsoids."

3DGS does facilitate editing tasks after training, which is a beneficial feature. In plant reconstruction the background is unnecessary. We have added post-processing background removal of these Gaussians to provide better rendering performance. This demonstrates an advantage of Gaussian splatting for easy manipulation of the scene after reconstruction.
Information about this can be found at the end of the Training section and in the "The Effect of Background Removal on Synthetic View Quality" section

Reviewer 2

4. "The term Digital twin is used in the paper without a clear definition, I propose to remove it or add a

definition that matches your work."

We agree. In our case as we not performing simulations or measuring dynamics of the models, we opt to remove the term from the manuscript and focus only on 3D reconstruction.

5. "In the paper (section 3D reconstructions method), the definition of SfM is not clear. It is mentioned that SfM extends multi-view stereo (MVS) but to our best knowledge it is the other way around. SfM produces a sparse point cloud by which can be converted to a dense point cloud by applying MVS."

We thank the reviewers for pointing this out, we agree that our wording here required improvement. When we referred to extension, we meant that SfM is designed to also calculate unknown camera poses, which MVS is not designed to do. However, MVS can take the camera poses calculated by SfM, and generate a much denser point cloud. To clarify this distinction, we have reworded both SfM and MVS in the '3D Reconstriction method' section. We have also added comparisons against MVS below.

6. "In table 7, is SfM or MVS applied?"

In our original submission, we only compared the SfM sparse point clouds against our 3DGS and NeRF point clouds. We realise now that this might be misleading to audiences and overstates the effectiveness of view synthesis models compared to traditional photogrammetry methods. Hence, we have extended this paper to also include MVS, generated using the dense reconstruction methods offered by COLMAP. These have been included in table 7 and added reference to this in the methodology and evaluation sections.
We have also now added a comparison of each point generated by each reconstruction method compared to the ground truth. This provides more information on the accuracy of each point, regardless of sparsity of the overall point cloud.
We make a detailed comparison between the benefits and drawbacks of 3DGS and NeRF compared to MVS in the new discussion section titled 'Comparison of View Synthesis Models and Traditional 3D Reconstruction Techniques'. We find that MVS offers much better performance than SfM, but the reconstruction accuracy of NeRF and 3DGS is higher.

7. "It is mentioned that MVS has high computational costs, and the quality reliant on the precision of the registered camera poses. Both are true, but these reason are both
applicable for NeRF and 3DGS as well; this is confirmed by the results, showing that the camera poses need to be optimized to improve the results."

We agree. NeRF and 3DGS also require accurate camera poses to generate effective reconstructions. We originally mentioned this in the 'Automated Dataset Capture' section before, but we have now added a more direct discussion of this at the end of the 'Comparison of View Synthesis Models and Traditional 3D Reconstruction Techniques' section.

8. "According to the authors a high quality camera is used. This statement, is subjective, and to our honest opinion debatable. The realsense D435 does not create sharp RGB images due to the fixed focus and small sensor. The contrast is especially large if you compare it with industrial camera's; which are often used in phenotyping systems. We understand that depth images are necessary, but then the D405 might have been a better choose given the baseline and distance between the plants and camera."

We agree that the D435 camera does offer lower quality (1920x1080) compared to other cameras used for phenotyping experiments (typically 4K resolution), one of the purposes of the paper is to show that an expensive, high resolution cameras are not strictly required to generate these dense reconstructions using view synthesis models. We have added discussion of this point to the "Robotic Imaging Setup" section.

9. "Figure 2 is a sparse point cloud from SfM. This should be replaced by a reconstruction with MVS."

We have now added an MVS reconstruction to this figure. We hope this adds clarity and highlights the difference between these different methods.

10. "With masking, connected components is applied to focus on the mask. The erosion process, might

cause tiny stems to disappear, which could introduce errors in the system."

We agree. We utilised this process to avoid background pixels being included within the masks. However, our masking process can cause issues with stems not being consistent between masks, and we believe this is why our foreground images sometimes failed to produce an accurate 3D plant model. In our original manuscript we acknowledged the masking process did not produce the best results. To add clarity, we have now mentioned this further in the "View Capturing Pipeline" section and discussed how this impacted feature extraction in the same section. We also add a new background removal approach, discussed below.

11. "The calibration method with respect to the turntable is fully undescribed. Inaccuracies in this calibration could explain the necessity for the 'refinement' of the camera poses."

We originally chose not to include the calibration details as we thought some audiences may not wish to read this. However, following the reviewer comments it is clear that our approach is relevant and should be discussed. We have included a detailed description in the supplementary material to avoid increasing the length of the paper further. We have added a reference to this at the bottom of the "Robotic Imaging Setup" section.

12. "Ground truth is compared with the EinScan reporting an accuracy of 0.1mm, but this 0.1mm is likely not applicable for plants with little features. It would be good to include repeated measurements from the EinScan to validate the ground truth device."

To measure the accuracy of the EinScan data, we have now performed a separate study in which we scanned an imitation plant, in order to establish an accurate ground truth of an object with the complex structure and challenging texture of a real-life counterpart. We scanned the plant from scratch 5 times, and quantified the consistency of the point clouds generated by the scans. We also compare against a high-quality scan captured using a μCT scanner at our institution. We found that the average distance between each of the scans was 0.76mm, while the average point accuracy between each scan and the ground truth was 0.75mm. This error rate is indeed higher than the manufacturer's claim of 0.1mm, potentially because the scanner often failed to capture all regions of the plant. These regions produced a much higher inaccuracy metric. However, we feel this accuracy is sufficient to act as a ground truth to our 3D reconstruction methods, and we have added this information to the "Data Acquisition" section.

13. "The results in Table 5 are a bit vague. 'SfM uses a similar bundle adjustment approach to our refinement step'. Similar is not the same so why was it not possible to align the approach?"

We apologise that our description here was not clear. SfM uses the same bundle adjustment process as we used for refining our camera poses. The main difference is that SfM must determine the camera poses first, before performing global bundle adjustment, while our approach does not (these are derived from the calibrated robot positions). We have clarified this and added more detail in the "A Comparison of Robot-derived and SfM Calculated Camera Poses" section.

14." Furthermore, the setup is not designed for SfM. It is known that SfM struggles with turntables if the background is partly visible. Table 5 could therefore be extended with the masks in the alignment step. In addition, the turntable is completely white, any additional markers could have been added to improve results, and gives SfM a chance to compete with the poses from the robot."

We agree that turntables can be troublesome for SfM (as well as 3DGS and NeRF), however we chose a turntable to allow the maximum view radius to be captured for even larger plants. We did include masks within the alignment step, however this was not clear in our original submission so have added this information at the bottom of the "A Comparison of Robot-derived and SfM Calculated Camera Poses" section. For our robot setup, masks were also included in the camera refinement process, which we have added and discussed in the "Robotic Imaging Setup" section.
We did include a blue and red checkerboard on top of the turntable to help the feature extraction and matching process in SfM/MVS. While further markers could have been used, such as QR codes/checkerboard patterns on stands extended away from the plant, we chose to avoid this to decrease the number of additional objects being reconstructed. We have added this information in the "Robotic

Imaging Setup” section.

15. "In table 5 the Euclidean distance could be added. How inaccurate is SfM? Maybe only a few cameras were incorrect?"

We agree that it is important to explore the accuracy of the SfM camera poses, as these have a direct impact on the reconstructions produced by NeRF and 3DGS. We have now conducted additional experiments to explore this. We first calculated a mapping between the SfM coordinate system and our own setup. To do this, we manually rescaled, rotated and transformed the SfM point cloud until it aligned with our point cloud, and then used an iterative closest point alignment algorithm (with scaling) to accurately register both point clouds. We then further refined the mapping via optimisation using a differential evolution algorithm.
We then calculated the average Euclidean distance between each of the SfM and robot image transforms. We assigned a threshold of 1mm as the greatest permissible distance above which a camera pose was not successfully determined. We removed unsuccessful views when performing a comparison of the image reconstruction quality using SfM views and our own system, in Table 5. This ensures that SfM is evaluated using only its successful views.
Overall, the SfM results were still worse than our robot transforms, mainly because SfM often failed to calculate transforms for all the images. Hence, even if SfM was able to calculate accurate transforms, the overall reconstruction quality was impacted due to some images not being included in training.

16. "The results of the reconstruction accuracy contains many items that should be part of the methodology section."

Thank you for this comment. We have now moved much of this into the training section, and combined it with the existing information in this section about point cloud generation.

17. "Are the results in Table 7 made with different camera poses? If yes, then the comparison might not be correct."

The results in Table 7 were produced using the same camera poses for SfM, MVS, 3DGS and NeRF. We have now included this information in the “Reconstruction Accuracy” section.

18. "Important details of the reconstruction evaluation method are missing. The SfM contains less points, did this influence the reconstruction accuracy. And what about points missing?"

SfM contained far fewer points than the 3DGS and NeRF point clouds. We have added the average number of points that SfM generated in the “Reconstruction Accuracy” section. In particular, there were fewer points on the thin stems of the wheat plants, due to there being less pixel data / texture information for point matching. We have now noted this in the “Reconstruction Accuracy” section.
This did impact the accuracy measurements. However, the SfM points were still less accurate than the average points generated from NeRF or 3DGS. To show this, we now also included a comparison of the SfM, MVS, NeRF and 3DGS points to the ground truth (rather than just the ground truth to the points). This shows that SfM still generated less accurate points, without this metric being impacted by the sparseness of the point cloud. We have added these results in Table 7, and have written more information about this in the “Reconstruction Accuracy” section. We note also that we have now included MVS in these comparisons, which is perhaps a fairer test of these techniques.

- The discussion is unfortunately not elaborate enough. Several important assumptions/details are missing;

19. * "Discussion on influence of turn table"

While the turntable allowed many more views to be captured around the plant with our setup as it is configured, it did cause several challenges. We have added some more detail in the “Automated Dataset Capture” section.

20. * "Limited comparison with results from literature"

We found that there is currently limited published research in this area, making it difficult to directly compare results. Our dataset is of captured wheat plants, which are different to most captured datasets. However, we have added some comparisons with other literature in the Discussion section. We compared our render quality to the ‘Evaluating NeRFs for 3D Plant Geometry Reconstruction in Field Conditions’- which captured a series of plants and generated a series of NeRF scenes from this. We found that our results were similar. In addition, we compared our reconstruction accuracy results to that generated in ‘Exploring Accurate 3D Phenotyping in Greenhouse through Neural Radiance Fields’. We found that their accuracy was roughly 1mm lower than ours, we have added text noting that this is likely due to inconsistencies with the Einstar scanner, which often failed to capture thin areas of the plant.

21. * "New information presented; size of the files; rendering of images"

We have added a new section titled “Comparison of View Synthesis Models and Traditional 3D Reconstruction Techniques” which mentions these factors and compares them to SfM and MVS.

22. * "The framework is presented as user-friendly, customable can deal with various plant sizes. However, in the first part of the paper 3 different designs were proposed that all struggled with capturing all details. Is this framework that flexible? To judge that the sizes of all plant must be mentioned somewhere, but this is to our best knowledge missing."

Our exploration of potential configurations was designed to discover which offered the most practical setup, while also capturing the largest range of views for our typical plant sizes. We did mention that our setup can capture views ranging from 0.3m to 1.5m, but not the exact sizes of the plants that it can capture. We have now added extra clarification to state what range of plant heights it can capture effectively in the ‘Robotic Imaging Setup’ section.
Our use of the term flexible in the manuscript was meant to suggest that our setup was adaptable, and could still capture all possible views that could be reached by each setup. This remains the case even if a particular setup is not well suited to capturing the broadest range of possible views. We have added a brief explanation of this in the “Automated Dataset Capture” section.

23. * "It is mentioned that the systems is novel since everything is in metric units. To our opinion this is incorrect. In almost all commercial and non-commercial SfM/MVS software calibration is standardized, which could have been applied in this research as well."

We did not claim our system of metric units is novel, however perhaps our wording or discussion of the phrase gave this impression. We agree with the reviewers that our system is not novel for utilising metric units. MVS software does support metric units if the setup is already calibrated this way. However, in our experience SfM and similar bundle adjustment approaches do not generate camera poses in accurate metric units. This will be particularly true for ad-hoc setups based on mobile devices, or potentially setups in the field with data captured live. We agree that for some installations where pre-calibration is an option then these systems could and probably would use metric units. We emphasise that our setup can capture in metric units, as the majority of other plant datasets captured for view synthesis reconstructions require SfM to acquire the camera poses (which does not typically produce camera poses in metric units).

24. * "In the discussion it is mentioned that 'post-processes to remove these gaussians were more effective than adapting the image capture of 3DGS training process'. This is an interesting finding, but those finding cannot be concluded from the results."

Thank you for this comment, this is a very valid point. We have now included a new experiment in which we incorporated post-processing background removal functions to cull the background Gaussians. Information about this can be found at the end of the “Training” section and in the “The Effect of Background Removal on Synthetic View Quality” section. We found that the post-processed rendered images produced a PSNR masked metric of 17.87db, while incorporating the foreground images during training produced a PSNR masked metric of 13.75db. Hence, removing these Gaussians after training produced more accurate results compared to altering the input data, which is another benefit of 3DGS compared to continuous models such as NeRF that are not well suited for alterations after training.
