# Supplementary material for: High-fidelity wheat plant reconstruction using 3D Gaussian splatting and neural radiance fields
Source: Gigascience. 2025 Mar 26;14:giaf022. doi: 10.1093/gigascience/giaf022 (PMC11945317; doi:10.1093/gigascience/giaf022)
Supplement: giaf022_Response_to_Reviewer_Comments_Revision_1 [file giaf022_response_to_reviewer_comments_revision_1.pdf]

Author's Response To Reviewer Comments

We thank the reviewers and editors for their helpful comments on our revisions. We are pleased that the reviewer enjoyed reading the paper, and felt we had adequately addressed the previous round of comments. The reviewer raises several minor comments which we have addressed in this new revision. We respond to all points below, and submit a marked up copy of the manuscript with changed highlighted in red.

Reviewer #2

1) page 2 - typo - recent progress in deep learning has led (not lead).

We have corrected this typographical error.

2) Table 7 - explain in the caption the SD and also the meaning of it better in the text.

We have added an explanation to the reconstruction results table caption, as well as in the text above.

3) Figure 6 - The presence of soil/ pot edge does not help in the results, to me it seems 3DGS is affected negatively by this, while it's not a biologically relevant distance error. Please remove for all 4 reconstruction methods.

We have recalculated the reconstruction accuracy results but with only points on the plant. This has lead to a slight change in the numerical results, in particular the accuracy of 3DGS improved above its original value. We have also adapted and extended the discussions of these results in the relevant sections of the manuscript.

4) Table 8 - 2nd column please in minutes, not seconds for readability.

We have adapted this column to minutes and added a description in the caption.

5) I propose to mention conveyor systems in controlled environment that allow transporting and scanning 1000s of plants/ 100s of genotypes within one experiment fully automated as the dot on the horizon for the excellent technological exploration described in this paper.

…

In the section Automated Dataset Capture, please add a paragraph how you can scale up towards fully automated high throughput phenotyping, and screen those 1000s of plants. I do not expect this solution will work on fields, therefore indoor phenotyping seems a logical next step. and then which challenges are then still to overcome? One of the aspects that need to be emphasized is that trait calculations are still quite challenging, and maybe different 3D modalities offer difference challenges/ and open source available solutions?

We agree that this discussion would add better helpful into the future of 3D plant phenotyping. We have added new section in the discussion that predicts what phenotyping problems 3DGS or NeRF will be used

for. We have also included a section at the end of the discussion that discusses how our setup can be upgraded into a larger scale platform for automated phenotyping.
