# Supplementary material for: High-fidelity wheat plant reconstruction using 3D Gaussian splatting and neural radiance fields
Source: Gigascience. 2025 Mar 26;14:giaf022. doi: 10.1093/gigascience/giaf022 (PMC11945317; doi:10.1093/gigascience/giaf022)
Supplement: giaf022_Response_to_Reviewer_Comments_Revision_2 [file giaf022_response_to_reviewer_comments_revision_2.pdf]

Author's Response To Reviewer Comments

Dear Editor,

Many thanks for your continued handling of our manuscript. We have updated this revised version as per your comments on the previous revision. We have also reduced the size of some of the high-resolution images to produce a more accessible file size for the manuscript.

Best wishes,

Michael Pound, Lewis Stuart
