# Supplementary material for: High-fidelity wheat plant reconstruction using 3D Gaussian splatting and neural radiance fields
Source: Gigascience. 2025 Mar 26;14:giaf022. doi: 10.1093/gigascience/giaf022 (PMC11945317; doi:10.1093/gigascience/giaf022)
Supplement: giaf022_Reviewer_1_Report_Original_Submission — Deli Zhu -- 9/7/2024 [file giaf022_reviewer_1_report_original_submission.pdf]

Reviewer Report

Title: High-fidelity Wheat Plant Reconstruction using 3D Gaussian Splatting and Neural Radiance Fields

Version: Original Submission **Date:** 9/7/2024

Reviewer name: Deli Zhu

Reviewer Comments to Author:

This research develop a camera capture system using two robotic arms combined with a turntable, controlled by a re-deployable and flexible image capture framework. They released all robot configuration and control software alongside our extensive multi-view dataset. And also all scripts necessary to train both 3DGS and NeRF, all trained models, and final 3D point representations were released. It is amazing.But there are something to revise.
(1).The research used both 3DGS and NeRF to verify the effectiveness of the method.Generally speaking, 3DGS has better reconstruction efficiency than NeRF. Why doesn't the author put effort into improving the 3DGS method to achieve better results in reconstruction? Only through the comparison of two ready-made methods, it is insufficient to reflect the progressiveness of the research.Suggest the author to conduct more methodological research to achieve convincing results.
(2)3D Gaussian Splatting is an explicit representation method that utilizes a set of Gaussian ellipsoids to model the scene, thereby achieving fast new view synthesis rendering speed. This article lacks effective analysis on the speed of various methods such as SMF, 3DGS, NERF, etc. I hope it can be supplemented.
(3).The characteristics of 3D Gaussian Splatting facilitate editing tasks such as dynamic reconstruction, geometric editing, and physical simulation. Further experiments and comparative analysis are needed to improve rendering efficiency and solve discontinuous shading problems by introducing Gaussian ellipsoids.

Methods

Are the methods appropriate to the aims of the study, are they well described, and are necessary controls included? Choose an item.

Conclusions

Are the conclusions adequately supported by the data shown? Choose an item.

Reporting Standards

Does the manuscript adhere to the journal's guidelines on minimum standards of reporting? Choose an item.

Choose an item.

Statistics

Are you able to assess all statistics in the manuscript, including the appropriateness of statistical tests used? Choose an item.

Quality of Written English

Please indicate the quality of language in the manuscript: Choose an item.

Declaration of Competing Interests

Please complete a declaration of competing interests, considering the following questions:

- Have you in the past five years received reimbursements, fees, funding, or salary from an organisation that may in any way gain or lose financially from the publication of this manuscript, either now or in the future?
- Do you hold any stocks or shares in an organisation that may in any way gain or lose financially from the publication of this manuscript, either now or in the future?
- Do you hold or are you currently applying for any patents relating to the content of the manuscript?
- Have you received reimbursements, fees, funding, or salary from an organization that holds or has applied for patents relating to the content of the manuscript?
- Do you have any other financial competing interests?
- Do you have any non-financial competing interests in relation to this paper?

If you can answer no to all of the above, write 'I declare that I have no competing interests' below. If your reply is yes to any, please give details below.

I declare that I have no competing interests.

I agree to the open peer review policy of the journal. I understand that my name will be included on my report to the authors and, if the manuscript is accepted for publication, my named report including any attachments I upload will be posted on the website along with the authors' responses. I agree for my report to be made available under an Open Access Creative Commons CC-BY license (http://creativecommons.org/licenses/by/4.0/). I understand that any comments which I do not wish to be included in my named report can be included as confidential comments to the editors, which will not be published.

Choose an item.

To further support our reviewers, we have joined with Publons, where you can gain additional credit to further highlight your hard work (see: https://publons.com/journal/530/gigascience). On publication of this paper, your review will be automatically added to Publons, you can then choose whether or not to claim your Publons credit. I understand this statement.

Yes Choose an item.
