# Supplementary material for: High-fidelity wheat plant reconstruction using 3D Gaussian splatting and neural radiance fields
Source: Gigascience. 2025 Mar 26;14:giaf022. doi: 10.1093/gigascience/giaf022 (PMC11945317; doi:10.1093/gigascience/giaf022)
Supplement: giaf022_Reviewer_2_Report_Original_Submission — Rick van de Zedde -- 10/2/2024 [file giaf022_reviewer_2_report_original_submission.pdf]

Reviewer Report

Title: High-fidelity Wheat Plant Reconstruction using 3D Gaussian Splatting and Neural Radiance Fields

Version: Original Submission **Date:** 10/2/2024

Reviewer name: Rick van de Zedde

Reviewer Comments to Author:

This paper was also co-reviewed by my colleague Bart van Marrewijk (Orcid 0000-0003-3681-8114). The comments below reflect both of our opinions.
The paper focusses on "view synthesis" and point cloud reconstructions using NeRF, 3DGS and SfM. A comparison is made with ground truth RGB images not present in the training pipeline, evaluating the accuracy of the reconstructed images. Further, the quality of the point cloud is assessed by comparing with an EinScan scanner. The results presented paper are interesting and combined with the fact the authors want to make everything open-source the paper shows added value for the plant phenotyping community. Despite, serious improvements are necessary, these improvements are listed below.
- The term Digital twin is used in the paper without a clear definition, I propose to remove it or add a definition that matches your work.
- In the paper (section 3D reconstructions method), the definition of SfM is not clear. It is mentioned that SfM extends multi-view stereo (MVS) but to our best knowledge it is the other way around. SfM produces a sparse point cloud by which can be converted to a dense point cloud by applying MVS.
- In table 7, is SfM our MVS applied?
- It is mentioned that MVS has high computational costs, and the quality reliant on the precision of the registered camera poses. Both are true, but these reason are both applicable for NeRF and 3DGS as well; this is confirmed by the results, showing that the camera poses need to be optimized to improve the results.
- according to the authors a high quality camera is used. This statement, is subjective, and to our honest opinion debatable. The realsense D435 does not create sharp RGB images due to the fixed focus and small sensor. The contrast is especially large if you compare it with industrial camera's; which are often used in phenotyping systems. We understand that depth images are necessary, but then the D405 might have been a better choose given the baseline and distance between the plants and camera.
- Figure 2 is a sparse point cloud from SfM. This should be replaced by a reconstruction with MVS.
- With masking, connected components is applied to focus on the mask. The erosion process, might cause tiny stems to disappear, which could introduce errors in the system.
- The calibration method with respect to the turntable is fully undescribed. Inaccuracies in this calibration could explain the necessity for the "refinement" of the camera poses.
- Ground truth is compared with the EinScan reporting an accuracy of 0.1mm, but this 0.1mm is likely not applicable for plants with little features. It would be good to include repeated measurements from the EinScan to validate the ground truth device.
- The results in Table 5 are a bit vague. "SfM uses a similar bundle adjustment approach to our refinement step". Similar is not the same so why was it not possible to align the approach?

- Furthermore, the setup is not designed for SfM. It is known that SfM struggles with turntables if the background is partly visible. Table 5 could therefore be extended with the masks in the alignment step. In addition, the turntable is completely white, any additional markers could have been added to improve results, and gives SfM a chance to compete with the poses from the robot.
- In table 5 the Euclidean distance could be added. How inaccurate is SfM? Maybe only a few cameras were incorrect?
- The results of the reconstruction accuracy contains many items that should be part of the methodology section.
- Are the results in Table 7 made with different camera poses? If yes, then the comparison might not be correct.
- Important details of the reconstruction evaluation method are missing. The SfM contains less points, did this influence the reconstruction accuracy. And what about points missing?
- The discussion is unfortunately not elaborate enough. Several important assumptions/details are missing;
* Discussion on influence of turn table
* Limited comparison with results from literature
* New information presented; size of the files; rendering of images
* The framework is presented as user-friendly, customable can deal with various plant sizes. However, in the first part of the paper 3 different designs were proposed that all struggled with capturing all details. Is this framework that flexible? To judge that the sizes of all plant must be mentioned somewhere, but this is to our best knowledge missing.
* It is mentioned that the systems is novel since everything is in metric units. To our opinion this is incorrect. In almost all commercial and non-commercial SfM/MVS software calibration is standardized, which could have been applied in this research as well.
* In the discussion it is mentioned that "post-processes to remove these gaussians were more effective than adapting the image capture of 3DGS training process". This is an interesting finding, but those finding cannot be concluded from the results.

Methods

Are the methods appropriate to the aims of the study, are they well described, and are necessary controls included? Choose an item.

Conclusions

Are the conclusions adequately supported by the data shown? Choose an item.

Reporting Standards

Does the manuscript adhere to the journal's guidelines on minimum standards of reporting? Choose an item.

Choose an item.

Statistics

Are you able to assess all statistics in the manuscript, including the appropriateness of statistical tests used? Choose an item.

Quality of Written English

Please indicate the quality of language in the manuscript: Choose an item.

Declaration of Competing Interests

Please complete a declaration of competing interests, considering the following questions:

- Have you in the past five years received reimbursements, fees, funding, or salary from an organisation that may in any way gain or lose financially from the publication of this manuscript, either now or in the future?
- Do you hold any stocks or shares in an organisation that may in any way gain or lose financially from the publication of this manuscript, either now or in the future?
- Do you hold or are you currently applying for any patents relating to the content of the manuscript?
- Have you received reimbursements, fees, funding, or salary from an organization that holds or has applied for patents relating to the content of the manuscript?
- Do you have any other financial competing interests?
- Do you have any non-financial competing interests in relation to this paper?

If you can answer no to all of the above, write 'I declare that I have no competing interests' below. If your reply is yes to any, please give details below.

I declare that I have no competing interests

I agree to the open peer review policy of the journal. I understand that my name will be included on my report to the authors and, if the manuscript is accepted for publication, my named report including any attachments I upload will be posted on the website along with the authors' responses. I agree for my report to be made available under an Open Access Creative Commons CC-BY license (http://creativecommons.org/licenses/by/4.0/). I understand that any comments which I do not wish to be included in my named report can be included as confidential comments to the editors, which will not be published.

Choose an item.

To further support our reviewers, we have joined with Publons, where you can gain additional credit to further highlight your hard work (see: https://publons.com/journal/530/gigascience). On publication of this paper, your review will be automatically added to Publons, you can then choose whether or not to claim your Publons credit. I understand this statement.

Yes Choose an item.
