# Supplementary material for: High-fidelity wheat plant reconstruction using 3D Gaussian splatting and neural radiance fields
Source: Gigascience. 2025 Mar 26;14:giaf022. doi: 10.1093/gigascience/giaf022 (PMC11945317; doi:10.1093/gigascience/giaf022)
Supplement: giaf022_Reviewer_2_Report_Revision_1 — Rick van de Zedde -- 1/11/2025 [file giaf022_reviewer_2_report_revision_1.pdf]

Reviewer Report

Title: High-fidelity Wheat Plant Reconstruction using 3D Gaussian Splatting and Neural Radiance Fields

Version: Revision 1 **Date:** 1/11/2025

Reviewer name: Rick van de Zedde

Reviewer Comments to Author:

I've enjoyed reading this paper, and the first and the second reviewer made excellent comments which has been sufficiently addressed by the 2nd version I received. NERF and Gaussian splatting are explored by many research groups, and this paper will become an excellent starting point for all these plant phenotyping researcher that are interested in measuring 3D plant traits with an automated system. I hope a 2nd paper will show up within a year in which this technology is integrated in an automated measuring system, appreciated and used by wheat breeders to screen their populations on numerous stresses.

BUT to trigger this, a few essential aspects (via minor changes) need to be more thoroughly explained in this paper to enable, especially, the readers with a data science, technical background to better understand the challenges ahead in large scale plant phenotyping.

In the introduction - I agree that 3D reconstruction of plants can be important in plant phenotyping pipelines, but only if the resulting integrated technology is able to scan large numbers of plants quickly, where the focus is on finding traits that are accurately measured which are relevant for the screening of - not a few plants - large numbers of genotypes, where the nr of replicates/ plants per genotype are chosen based on the statistical requirements to find differences when measuring these traits. Then 3D / shape related traits such as leaf angle (when leaves drop because of drought), internodia lengths (when plant growth is affected by lower light conditions, cold, diseases or salt or nutrient stress), etc. I propose to mention conveyor systems in controlled environment that allow transporting and scanning 1000s of plants/ 100s of genotypes within one experiment fully automated as the dot on the horizon for the excellent technological exploration described in this paper.

page 2 - type - recent progress in deep learning has led (not lead).

Table 7 - explain in the caption the SD and also the meaning of it better in the text.

Figure 6 - The presence of soil/ pot edge does not help in the results, to me it seems 3DGS is affected negatively by this, while it's not a biologically relevant distance error. Please remove for all 4 reconstruction methods.

Table 8 - 2nd column please in minutes, not seconds for readability.

In the section Automated Dataset Capture, please add a paragraph how you can scale up towards fully automated high throughput phenotyping, and screen those 1000s of plants. I do not expect this solution will work on fields, therefore indoor phenotyping seems a logical next step. and then which challenges are then still to overcome? One of the aspects that need to be emphasized is that trait calculations are still quite challenging, and maybe different 3D modalities offer difference challenges/ and open source available solutions?

Methods

Are the methods appropriate to the aims of the study, are they well described, and are necessary controls included? Choose an item.

Conclusions

Are the conclusions adequately supported by the data shown? Choose an item.

Reporting Standards

Does the manuscript adhere to the journal's guidelines on minimum standards of reporting? Choose an item.

Choose an item.

Statistics

Are you able to assess all statistics in the manuscript, including the appropriateness of statistical tests used? Choose an item.

Quality of Written English

Please indicate the quality of language in the manuscript: Choose an item.

Declaration of Competing Interests

Please complete a declaration of competing interests, considering the following questions:

- Have you in the past five years received reimbursements, fees, funding, or salary from an organisation that may in any way gain or lose financially from the publication of this manuscript, either now or in the future?
- Do you hold any stocks or shares in an organisation that may in any way gain or lose financially from the publication of this manuscript, either now or in the future?
- Do you hold or are you currently applying for any patents relating to the content of the manuscript?
- Have you received reimbursements, fees, funding, or salary from an organization that holds or has applied for patents relating to the content of the manuscript?
- Do you have any other financial competing interests?
- Do you have any non-financial competing interests in relation to this paper?

If you can answer no to all of the above, write 'I declare that I have no competing interests' below. If your reply is yes to any, please give details below.

I declare that I have no competing interests.

I agree to the open peer review policy of the journal. I understand that my name will be included on my report to the authors and, if the manuscript is accepted for publication, my named report including any attachments I upload will be posted on the website along with the authors' responses. I agree for my report to be made available under an Open Access Creative Commons CC-BY license (http://creativecommons.org/licenses/by/4.0/). I understand that any comments which I do not wish to be included in my named report can be included as confidential comments to the editors, which will not be published.

Choose an item.

To further support our reviewers, we have joined with Publons, where you can gain additional credit to further highlight your hard work (see: https://publons.com/journal/530/gigascience). On publication of this paper, your review will be automatically added to Publons, you can then choose whether or not to claim your Publons credit. I understand this statement.

Yes Choose an item.
